# Characterization of Ligand Binding in Human Serum Albumin from Atomistic Energy Transfer Simulations

**DOI:** 10.1002/smtd.202501820

**Published:** 2025-11-14

**Authors:** Özge Ergün, Andrea Bertran‐Mostazo, Elena Cubero, Carles Galdeano, Carles Curutchet

**Affiliations:** ^1^ Departament de Farmàcia i Tecnologia Farmacèutica i Fisicoquímica Facultat de Farmàcia i Ciències de l'Alimentació Universitat de Barcelona (UB) Barcelona 08028 Spain; ^2^ Institut de Química Teòrica i Computacional (IQTCUB) Universitat de Barcelona (UB) Barcelona 08028 Spain; ^3^ Institut de Biomedicina (IBUB) Universitat de Barcelona (UB) Barcelona 08028 Spain; ^4^ Gain Therapeutics Sucursal en España Barcelona Science Park Barcelona 08028 Spain

**Keywords:** energy transfer, FRET, human serum albumin, molecular dynamics, protein‐ligand binding

## Abstract

Förster resonance energy transfer (FRET) is a key biophysical method for probing nanometer‑scale distances in biomolecular systems, but its direct application to protein–ligand complexes suffers from substantial biases due to restricted chromophore orientations and the limited validity of the point‑dipole approximation. This study introduces a protocol for identifying binding sites and characterizing ligand coordination modes in situ by combining fluorescence spectroscopy with efficient atomistic simulations based on the TrESP‑MMPol model. The protocol integrates electrostatic potential‑fitted transition charges with a polarizable classical environment, thereby overcoming the orientation and dielectric‐screening assumptions inherent to Förster theory. The protocol has been applied to human serum albumin (HSA) and a library of fluorescent small molecules, including known binders of the HSA, accurately reproducing the binding sites of naproxen, carprofen, and indomethacin, and revealing novel binding scenarios for other molecules. The results show that direct comparison of experimental FRET data with atomistically simulated observables enables discrimination of plausible binding models – including the site and binding mode – and avoids systematic errors in distance estimation. The protocol is particularly attractive to examine targets with a single tryptophan, and can also be extended to other targets of interest in drug discovery via site‐labelling with unnatural amino acids.

## Graphical abstract

Förster resonance energy transfer (FRET) is used to identify binding sites and characterize ligand coordination modes by combining fluorescence spectroscopy with efficient atomistic simulations beyond Förster theory. The protocol is validated on known binders of human serum albumin and applied to study the binding of a set of fluorescent fragments, showing that atomistic simulations enable discrimination of plausible binding modes.

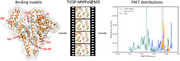

## 1 Introduction

Fluorescence is used in a variety of spectroscopic techniques to monitor structural changes in proteins^[^ ^1^, ^2^, ^3^, ^4^, ^5^, ^6^, ^7^, ^8^, ^9^ ^]^ due to the large sensitivity of tryptophan (Trp) emission on its molecular environment.^[^ ^4^, ^10^, ^11^, ^12^ ^]^ In protein‐ligand complexes, Trp deactivation by electronic energy transfer to a proximate ligand often occurs, and this information can be used to determine the Trp‐ligand separation, as usually done in the Förster resonance energy transfer (FRET) method.^[^ ^4^, ^13^, ^14^, ^15^, ^16^ ^]^ Similar analyses can be undertaken for the opposite transfer phenomena, where ligand fluorescence is quenched upon protein binding.^[^ ^17^, ^18^ ^]^ Because of its simplicity, fluorescence is indeed widely used to study the non‐covalent binding of small molecules to proteins, for example, to obtain binding affinities.^[^ ^19^ ^]^ Despite its potential, however, FRET analyses aimed at shedding light on the structure of the underlying protein‐ligand complex suffer from several complications, like inner‐filter effects or changes in excited state decay channels beyond FRET.^[^ ^19^ ^]^

A more fundamental problem hampering the application of FRET to protein‐ligand binding is related to the validity of Förster dipole approximation, leading to the *R* ^−6^ distance‐dependence of FRET.^[^ ^16^, ^20^ ^]^ In common FRET constructs, fluorophores are covalently attached to proteins through flexible linkers, giving them considerable rotational freedom. This allows a direct link between efficiency and donor/acceptor separation, assuming an isotropic dipole‐dipole orientation factor, κ^2^ = 2/3.^[^ ^21^ ^]^ In a protein‐ligand complex, however, the relative orientation of Trp residues and ligands is typically limited.^[^ ^18^ ^]^ The restricted rotational freedom of fluorophores attached to ligands is fundamental, for example, in fluorescence polarization binding assays. In addition, the dipole approximation breaks down at close Trp‐ligand separations, especially when the rotational freedom is restricted,^[^ ^22^ ^]^ and dielectric screening effects due to the polarizability of the environment introduce deviations with respect to the simple 1/*n* ^2^ factor adopted in Förster theory, which depends on the refractive index of the medium.^[^ ^18^, ^23^, ^24^ ^]^

A route to avoid Förster assumptions consists of simulating FRET data from structures obtained from molecular dynamics (MD) simulations.^[^ ^21^, ^25^, ^26^, ^27^, ^28^, ^29^, ^30^, ^31^, ^32^, ^33^, ^34^, ^35^, ^36^, ^37^, ^38^ ^]^ Donor–acceptor couplings can then be computed along the trajectories beyond Förster dipole approximation, and the validity of binding models (binding site and mode) generated in silico can be directly assessed by comparison to experimental data. We applied this strategy to rationalize the enantioselective fluorescence quenching observed in the complex between flurbiprofen and human serum albumin (HSA) using polarizable quantum/molecular mechanics (QM/MMPol) calculations, where FRET electronic couplings were estimated from QM transition densities with explicit inclusion of screening effects exerted by the atomistic environment.^[^ ^16^, ^18^, ^39^ ^]^ Routine application of this strategy to screen a variety of binding sites is, however, computationally expensive, and requires a careful identification of the participating excited states in the multiple QM/MMPol calculations performed along MD trajectories.

**Figure 1:**
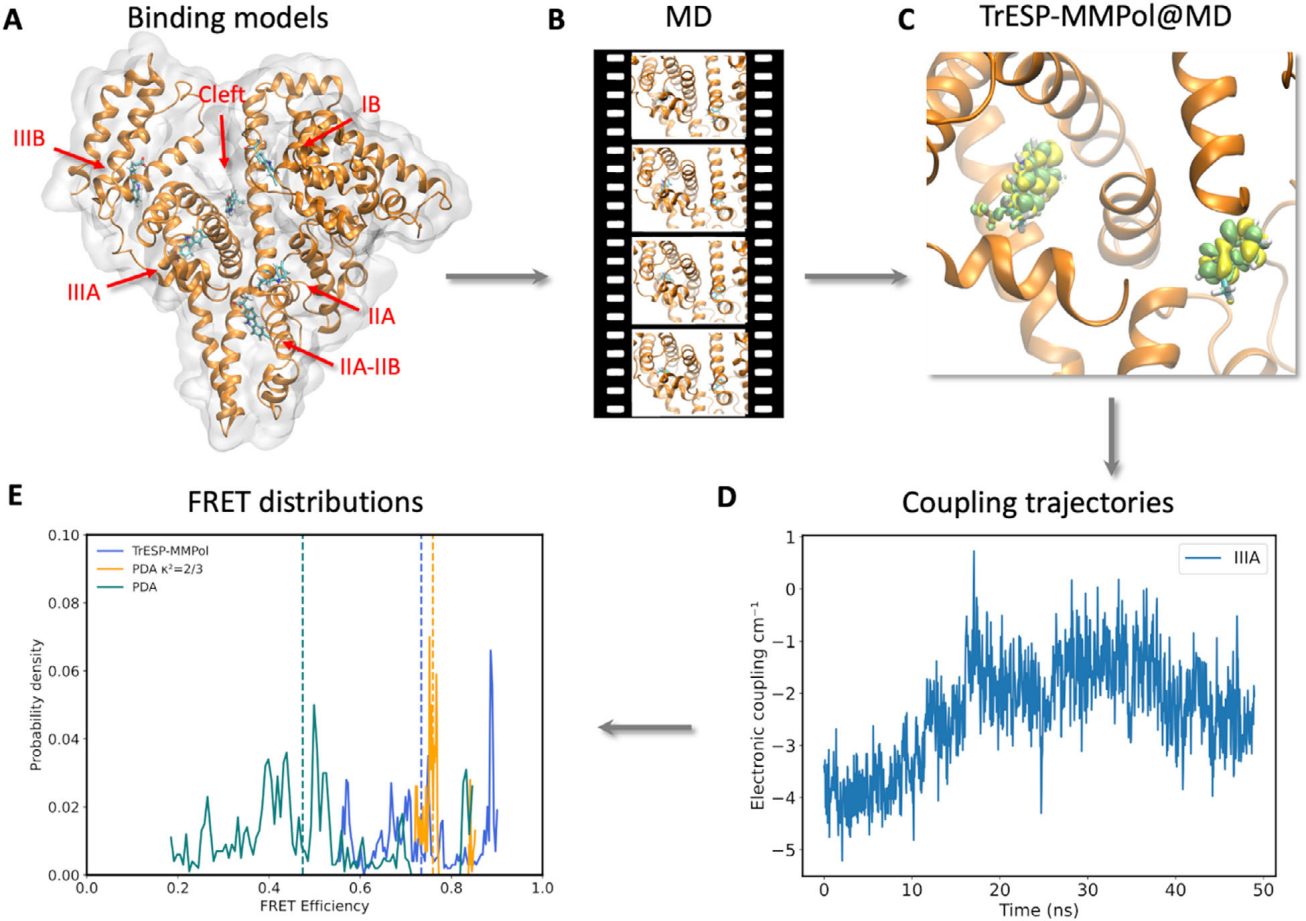
Protocol for the simulation of FRET distributions for binding modes of *(S)‐*carprofen in the binding sites of human serum albumin based on MD simulations and TrESP/MMPol calculations of electronic couplings. A) Molecular docking was used to generate plausible binding modes of the ligand in the six binding sites of HSA. Site IIA and IIIA correspond to Sudlow's site I (warfarin‐binding site) and II (benzodiazepine‐binding site), respectively. B) Classical MD simulations were performed to relax and sample the binding models of the ligand. C) The trajectory was postprocessed, performing TrESP/MMPol calculations every 50 ps of the electronic coupling that mediates FRET based on transition charges precomputed from TD‐DFT transition densities. The image illustrates transition densities of carprofen and tryptophan excitations. D) Fluctuations of electronic couplings were computed along MD trajectories. E) Distributions of FRET efficiencies were computed in an intermediate averaging regime to account for both dynamic and static fluctuations.^[^ ^16^ ^]^

Here, we have developed a protocol, shown in **Figure** **1**, that overcomes these limitations using the TrESP‐MMPol model,^[^ ^24^, ^40^ ^]^ which adopts electrostatic potential‐fitted transition charges coupled to an atomistic polarizable classical environment to estimate couplings in an efficient yet accurate way, thus avoiding the need to perform multiple QM calculations.^[^ ^24^ ^]^ We have applied this protocol, combined with fluorescence spectroscopy, to identify ligand binding sites and binding modes in HSA. HSA is the major transport plasma protein, which modulates the pharmacodynamic and pharmacokinetic profiles of drugs, and presents attractive features to investigate the limits of the protocol, namely the presence of multiple binding sites and a single Trp residue in position 214.^[^ ^41^, ^42^, ^43^, ^44^, ^45^ ^]^ This allows to investigate if our models are able to resolve FRET differences among binding sites spanning a range of Trp‐ligand distances from ≈5 up to ≈40 Å. We have applied the protocol to a library of fluorescent small molecules, including known binders of the HSA (naproxen, carprofen, and indomethacin) and other fragments. Crucially, we have detected binding through changes in the protein intrinsic fluorescence, whereas FRET has been used to obtain spatial resolution and identify the binding site involved and characterize the ligand binding pose. For molecules with positive binding, we have thus generated in silico binding models with specific poses in each binding site that are validated by comparison of measured and simulated FRET efficiencies.^[^ ^24^ ^]^

Overall, our results warn against the estimation of Trp‐ligand distances directly from FRET experiments and demonstrate the potential of atomistic simulation protocols for a more faithful interpretation of FRET data. Indeed, examination of FRET data compiled for a variety of ligands in HSA has been shown to lead to biased distributions characterized by rather large donor‐acceptor distances, inconsistent with the notion that both Sudlow's site I (IIA) and II (IIIA) are the major ligand binding sites.^[^ ^19^ ^]^ The protocol thus paves the way for extracting the rich structural data underlying FRET processes in common fluorescence‐based assays of protein‐ligand binding. The protocol can complement structure‐based drug design tools and techniques like structure‐based NMR, cryo‐EM, or X‐ray crystallography, and can be of special relevance in fragment‐based campaigns and drug screenings where allosteric pockets are aimed, where the target binding site is sometimes not elucidated. In this case, the protocol could allow a fast discrimination of the spatial regions of the target involved. In this context, additional fragment screenings can also be performed with larger collections of fluorescent molecules beyond the initial library adopted here, taking advantage of the large compilations of optical properties for small molecules currently available.^[^ ^46^ ^]^ Otherwise, chimeric fragment libraries can be prepared by attaching a known fluorophore to different fragments, and this has the advantage that all fragments share the same FRET properties, which reduces uncertainties in both the theoretical and experimental processing of results.^[^ ^44^ ^]^

We base our protocol on the intrinsic protein fluorescence, originated from Trp or other aromatic residues. This can be problematic if several Trp residues are present in a target, or if the Trp fluorescence is sensitive to structural changes upon ligand binding, which can lead to fluorescence quenching unrelated to FRET. In those cases, we envision improved protocols based on unnatural fluorescent amino acids,^[^ ^47^ ^]^ which enable site‐specific labeling of proteins through genetic engineering and avoid the complications of Trp emission. As an alternative, a dye can be attached to a given residue using, for example, maleimide chemistry, common to label Cys residues in dye‐labelled FRET constructs. However, linker flexibility in the latter case could diminish the capability of simulations to resolve binding models characterized by different ligand‐dye orientations. Nevertheless, site‐labelling with unnatural amino acids or extrinsic dyes in protein‐ligand complexes has the advantage of better fluorescent properties and the possibility to place the FRET “radar” in the region of interest in the biomolecule. Our atomistic protocol could be applied to such different situations and to characterize macromolecule recognition events linked to protein‐protein and protein‐DNA interactions. Alternatively, the protocol can also be applied to targets with multiple Trp residues, but this would require additional modelling of energy absorption and redistribution among the multiple chromophores, which presumably would lower the accuracy.^[^ ^16^ ^]^

## 2 Results and Discussion

### 2.1 Generation of Plausible Protein‐Ligand Binding Modes

**Figure 2:**
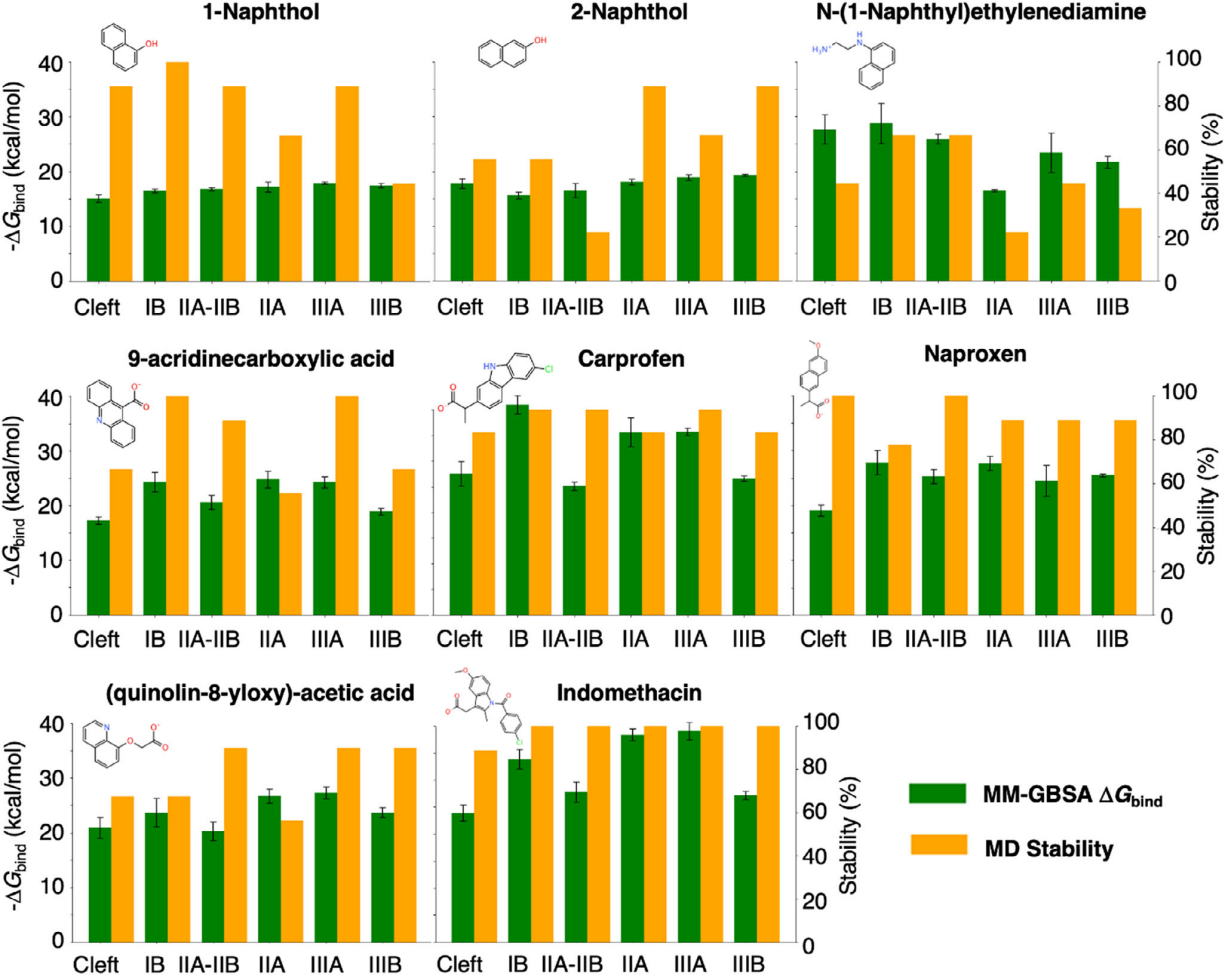
MM‐GBSA binding free energies and percent of stable replicas obtained from MD simulations started from the three best docking poses of the ligands in the six binding sites of HSA.

HSA is a protein that contains three helical domains (I‐III), each one involving two subdomains (A and B). The protein has two main hydrophobic binding sites for aromatic and heterocyclic molecules, sites IIA and IIIA, which correspond to Sudlow's site I (warfarin‐binding site) and II (benzodiazepine‐binding site), respectively, and four additional ligand binding sites have been described (Cleft, IB, IIA‐IIB, and IIIB), as shown in Figure 1.^[^ ^42^ ^]^ We thus performed molecular docking simulations to generate plausible binding models of all the ligands in the six binding sites, including the known HSA binders (*S*)‐carprofen, (*S*)‐naproxen and indomethacin, as well as the fluorescent fragments 1‐naphthol, 2‐naphthol, N‐(1‐naphthyl)ethylenediamine (NNE), 9‐acridinecarboxylic acid (ACA) and (quinoline‐8‐yloxy)‐acetic acid (QAA). In several cases, we found different orientations of the ligand in a protein binding site. For each site, we thus selected the three poses with the best docking scores and investigated the stability of these poses by MD simulations. In some cases, the ligand left the initial binding pocket. We thus estimated the stability of the ligand in each site by calculating the percentage of stable trajectories out of the nine total replicas started for the ligands in each pocket. The results are shown in **Figure** **2**. The drugs (*S*)‐carprofen, (*S*)‐naproxen, and indomethacin displayed the largest stability, with values >80% for almost all six binding sites. For the other ligands, we found stabilities >80% for ≈2–4 sites and ≈20–60% for the other, except for NNE, which was somewhat less stable. Binding to sites IIA‐IIB and IIIA was found to be slightly more robust than to the other sites.

We then performed an energetic analysis of the trajectories by computing MM‐GBSA binding free energies to shed more light on the relative stability of the ligands in each pocket. Again, (*S*)‐carprofen, (*S*)‐naproxen, and indomethacin showed the larger binding energies, in agreement with the stability discussed above. The energy differences for a given ligand between sites were however moderate, and the accuracy of MM‐GBSA does not allow to draw solid conclusions on the identification of the most favorable binding sites for each ligand. For cases like indomethacin, the binding sites IIA and IIIA displayed the largest binding affinities, followed by site IB, in reasonable agreement with the known binding of indomethacin to IB and IIA.^[^ ^42^ ^]^ For (*S*)‐carprofen and (*S*)‐naproxen, which are known to bind preferentially to site IIIA, binding affinities for that site were similar to those found for other ones. For ACA, simulations performed on the less stable zwitterionic protonated form (see Figure S1, Supporting Information) led to slightly lower stabilities and binding affinities compared to the deprotonated form, supporting the expectation that the latter form is mainly involved in HSA binding.

### 2.2 Energy Transfer Observables for Specific Ligand Binding Modes

The binding modes generated from docking and MD were then used to simulate the FRET observables expected for ligands bound in each site of HSA. Our aim was two‐fold. First, to investigate the performance of the TrESP‐MMPol@MD protocol compared to the dipole approximation to validate binding modes by comparison with experimental FRET data. In addition, we aimed to investigate the potential pitfalls involved in the common simple conversion of FRET measured efficiencies to structural data (Trp‐ligand distances), assuming Förster theory. We thus generated electronic coupling trajectories for the ensemble of stable MD replicas obtained for a given ligand in each binding site. We investigated three levels of accuracy: i) PDA@MD, based on the point dipole approximation (PDA) assuming a random isotropic distribution of Trp‐ligand orientations (κ^2^ = 2/3), ii) PDA@MD incorporating the κ^2^ values computed from the relative Trp‐ligand orientations sampled along MD trajectories, and iii) TrESP‐MMPol@MD with full account of the shapes of the ligands and the details of the environment using distributed sets of transition charges coupled to a polarizable force field description of the surroundings.

**Figure 3:**
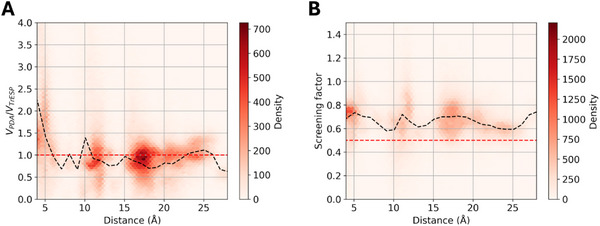
Deviations of Förster point dipole approximation with respect to atomistic TrESP‐MMPol electronic couplings computed for all ligand‐HSA complexes. A) Density distribution of ratios *V_PDA_*/*V_TrESP_* between (unscreened) PDA and TrESP coulombic coupling contributions as a function of D/A separation, i.e., *V_Coul_* in Equation (6). Black dashed curve: average over 1 Å distance bins; red dashed line: ideal *V_PDA_*/*V_TrESP_* ratio = 1. B) Density distribution of TrESP‐MMPol screening factors as a function of D/A separation. Black dashed curve: average over 1 Å distance bins; red dashed line: Förster factor s = 1/n^2^.

In **Figure** **3**, we show the significant deviations found for Förster dipole‐dipole and screening model to describe the interactions mediating FRET in the protein‐ligand complexes. The average values of the ratio *V_PDA_*/*V_TrESP_* between unscreened coulombic coupling terms (black curve in Figure 3A) tend to 1 at large donor/acceptor (D/A) distances, showing as expected larger deviations at close D/A separations. Nevertheless, significant deviations were still found at all distance ranges studied, as apparent from the density of data points departing from the ideal red line. Previous studies have shown that PDA deviations can persist at separations larger than the molecules dimensions when the rotational freedom is restricted.^[^ ^22^ ^]^ In Figure S2 (Supporting Information), we provide the plots for each ligand, which show that the ratios clearly tended to one, especially for smaller ligands like 1‐naphthol, 2‐naphthol, or NNE. However, for some large ligands like ACA or (*S*)‐naproxen, significant deviations were still found beyond 25 Å.

Whereas the PDA mostly breaks down at close D/A separations, Förster screening factor *s* = 1/*n* ^2^ can lead to important deviations at all D/A distance ranges. This is in line with other reports, which show that Förster factor leads to a systematic underestimation of screening and neglects its dependence on mutual D/A arrangement or differences in the local environment, for example, due to varying degrees of solvent exposure.^[^ ^16^, ^24^ ^]^ Here, we found significant deviations for protein‐ligand complexes. The atomistic TrESP‐MMPol screening factors shown in Figure 3B indicate that screening effects are significantly attenuated (larger *s* values) compared to Förster factor *s* = 1/*n* ^2^, indicated by the red line. In Figure S3 (Supporting Information), we show the individual distributions found for each ligand. Interestingly, the actual degree of screening depends on the specific details of the binding site, due to differences in amino acid composition or the degree of ligand solvent exposure, as one can identify different regions of points characterized by different D/A distances. In turn, screening effects do not markedly depend on the nature of the ligand. For example, for almost all ligands, at short distances ≈5 Å, we found *s* values ≈0.8, which are linked to binding in site IIA, Sudlow's site I, in contact with Trp214. The average MD distances sampled for the ligands on each site are provided in Table S3 (Supporting Information).

When D/A distances data are commonly extracted from experiments through Equation (3), based on the Förster critical radius *R_o_* of the dye pair, one further assumes an isotropic ensemble of D/A orientations. We also explored this assumption by computing the actual distribution of κ^2^ dipole‐dipole orientation factors, which we then compared to the ideal isotropic distribution in Tables S4–S12 (Supporting Information). Somewhat unexpectedly, and despite the limited conformational freedom of the donor Trp214, we obtained quite extended distributions of orientation factors. This is especially true for the smaller ligands 1‐naphthol, 2‐naphthol, which have a larger rotational freedom and displayed distributions close to the random isotropic assumption. For other ligands, however, the distributions were still far from this, and the average κ^2^ values deviate significantly from the isotropic limit, κ^2^ = 2/3.

**Figure 4:**
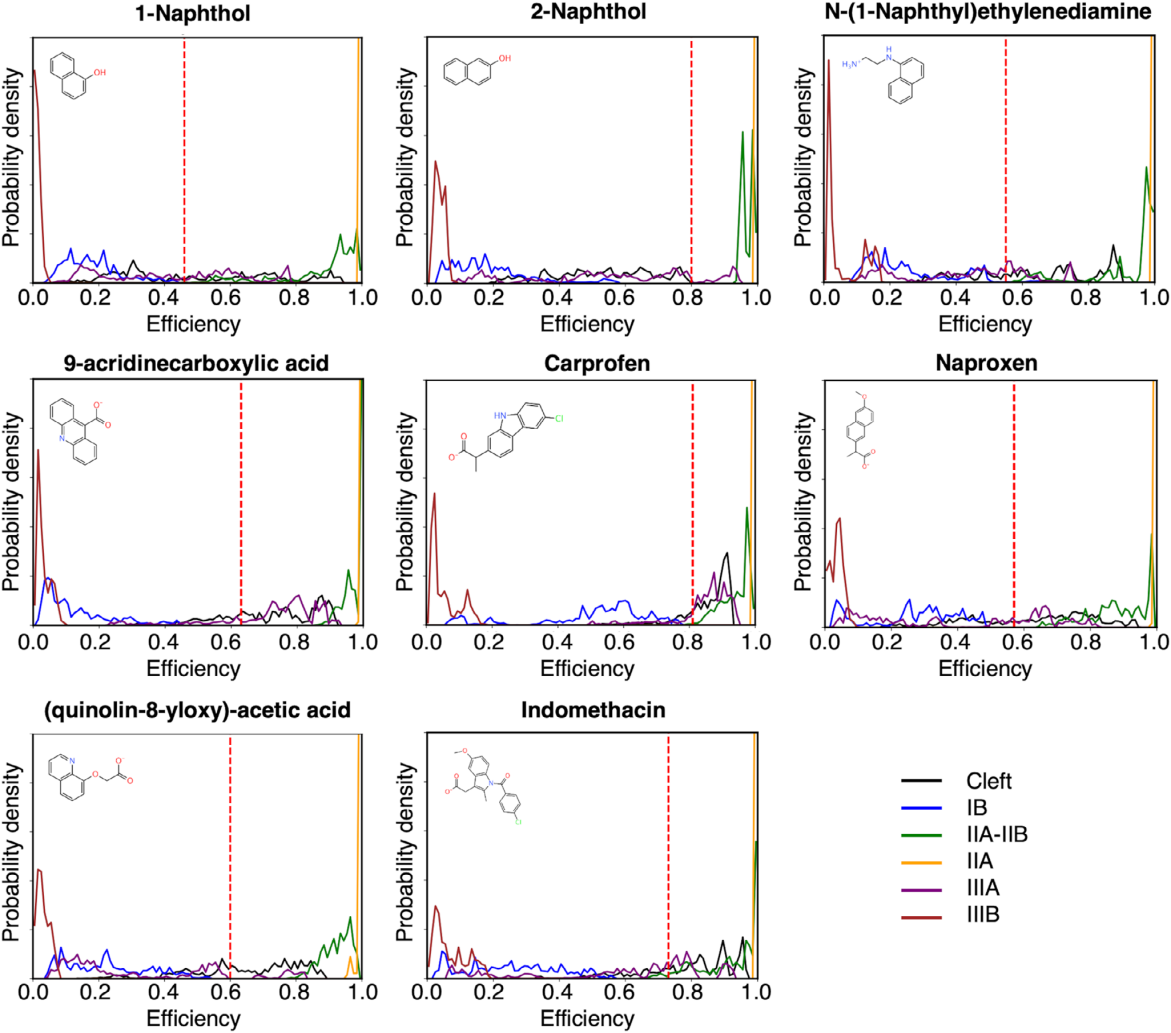
Distribution of FRET efficiencies for the ligands bound in the six binding sites of HSA: Cleft, IB, IIA–IIB, IIA, IIIA, and IIIB. Efficiencies were obtained from coupling trajectories using the TrESP‐MMPol@MD computational protocol—atomistic transition charge couplings in a polarizable environment. Vertical red lines indicate experimental FRET values derived from fluorescence ligand titrations.

We then calculated FRET efficiencies expected from each site, considering the orientational dynamics of the dyes using the expression in Equation (5), which allows to incorporate static and dynamic disorder by separating slow and fast fluctuations in instantaneous transfer rates, a timescale separation dictated by the fluorescence lifetime of HSA. In **Figure** **4**, we report the resulting distributions of FRET efficiencies computed using the rigorous TrESP‐MMPol@MD protocol, with experimental FRET values derived from fluorescence ligand titrations indicated by the vertical red lines. In Figures S13–S15 (Supporting Information), we compared these distributions with those obtained by PDA@MD protocols, considering specific MD orientations or assuming the isotropic limit. It can be observed how the overall TrESP‐MMPol@MD distributions deviated from Förster PDA models, leading to more extended ranges of efficiencies characterized by higher values, as it is apparent, for example, for the distributions arising from ligand binding in site IB. The distributions also showed that there is significant static disorder, and the ensemble efficiency values cannot reflect the richness of the underlying ensemble of conformations, which strongly impact the resulting FRET properties. In Table S5 (Supporting Information), we also report the errors in average efficiencies obtained using the PDA@MD protocols, which show that using the PDA on specific MD orientations leads to mean unsigned errors (MUE) up to 0.08 in the resulting efficiencies.

**Figure 5:**
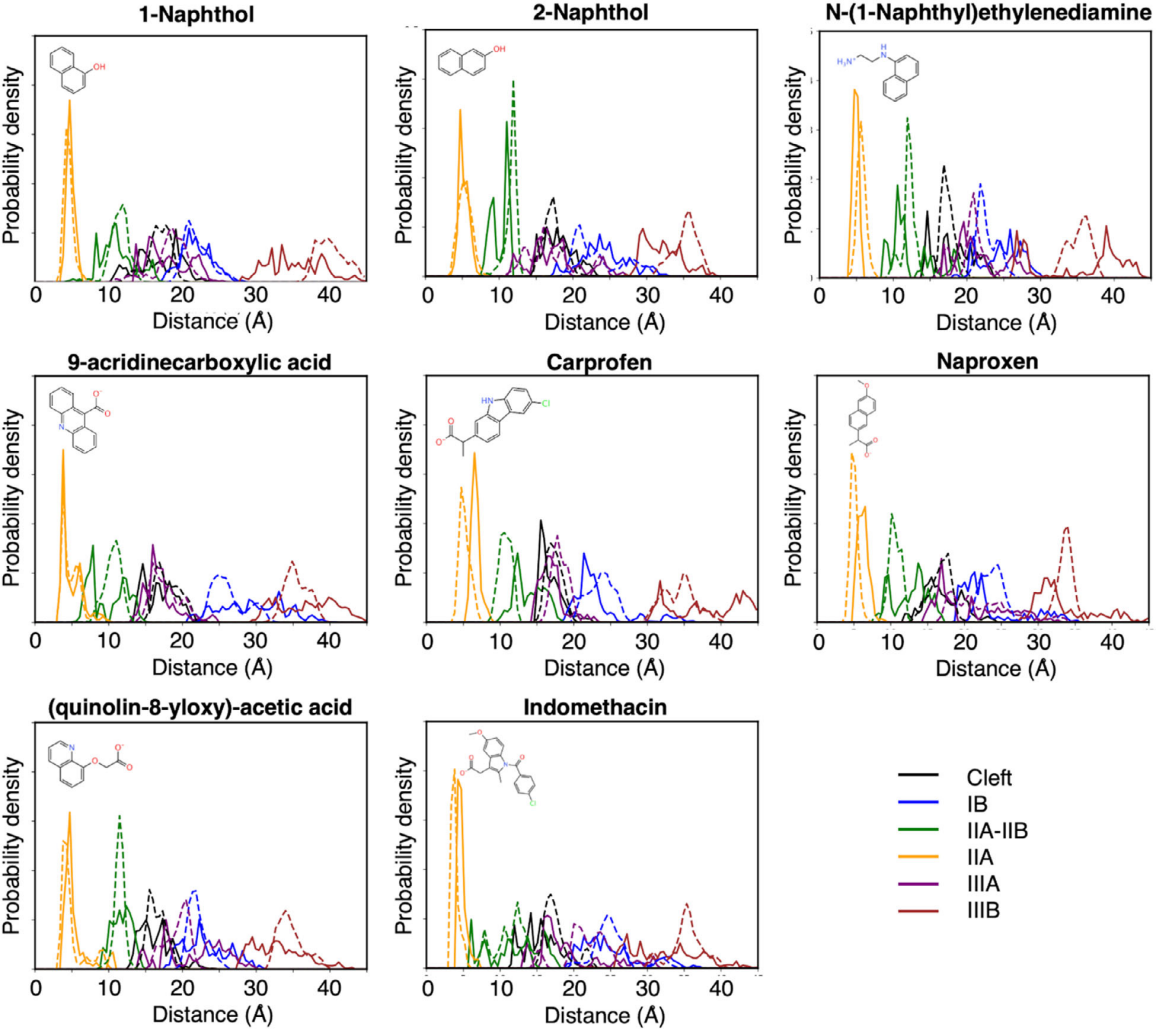
Comparison of donor–acceptor (D/A) distance distributions obtained directly from MD trajectories (dashed curves) with those derived from TrESP‐MMPol@MD FRET efficiency distributions transformed into distances using the Förster model and Equation (3) (bold curves) for the ligands in the six binding sites of human serum albumin (HSA): Cleft, IB, IIA–IIB, IIA, IIIA, and IIIB. Deviations between the two sets highlight the impact of the isotropic orientation assumption and the point dipole approximation, including dielectric‐screening effects, in standard Förster theory.

We then examined how Förster assumptions encapsulated in Equation (3), which relates efficiencies with distances, bias the experimental determination of D/A separations. To this end, we simulated an experimental efficiency distribution that corresponds exactly to our MD ensemble by using the TrESP‐MMPol@MD distributions shown in Figure 4. Then, we used Equation (3) to transform these efficiencies to D/A distances, which can be compared to the actual exact distances sampled along the MD trajectories. This analysis, reported in **Figure** **5**, showed that, in some cases, cancellation of errors in Förster model leads to pretty good distance distributions, for example, that found for the ligands in site IIA. At large separations, the errors can be very significant, as those found for ligands in site IIIB, the one located further from Trp214 at ≈35–40 Å, in which distances can be biased by >5 Å. In Table S3 (Supporting Information), we report the corresponding average distances for the ligands in each binding site, whereas in Table S4 (Supporting Information), we report a summary of the statistical errors in distances extracted from the efficiencies compared to the MD values. The results show that MUE errors amount to 1.4 Å for all systems considered, but increase up to 1.7 and 2.7 Å for the ligands in sites IB or IIIB, e.g., we find the largest deviation 7.0 Å, for protonated ACA in site IB. Notably, these results demonstrate that employing the TrESP‑MMPol@MD protocol, instead of the standard Förster model, reduces the average error in calculated Trp–ligand distances by ≈1.4 Å, but errors can be significantly larger for specific cases, especially at larger separations. This significant improvement underscores the necessity of atomistic simulations to avoid the systematic distance deviations inherent to Förster's approximation. More importantly, the extended distribution of FRET efficiencies is interpreted by Förster model as being originated from remarkable fluctuations in D/A separation, whereas MD narrower distributions showed they actually arise due to fluctuations in D/A orientation rather than separation. Estimated distances also tend to shift to lower D/A separations compared to MD data, an effect linked to the overestimation of screening effects in Förster theory, as shown in Figure 3.

### 2.3 Validation of Ligand Binding Modes from Fluorescence Spectroscopy

**Figure 6:**
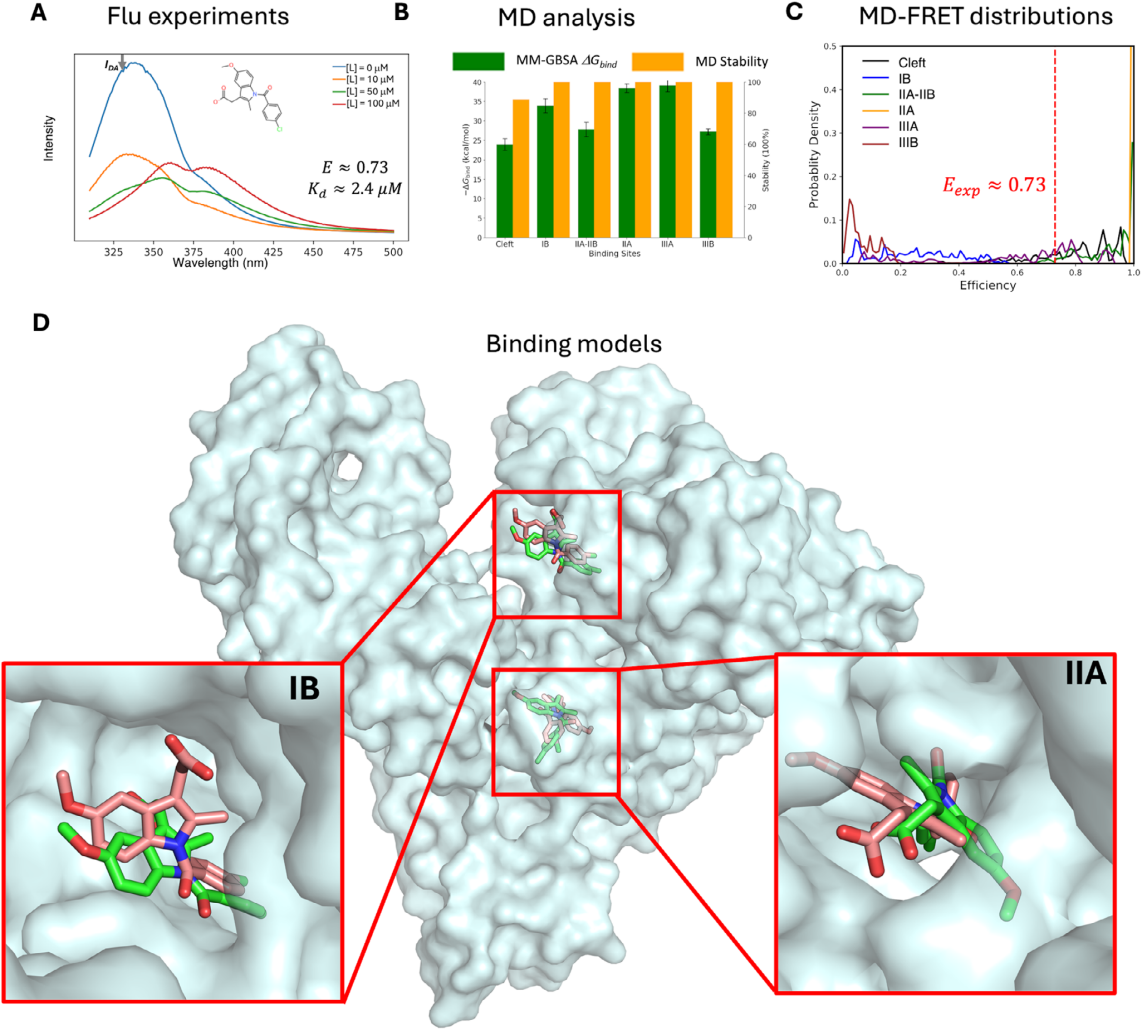
Binding modes determined for indomethacin using the TrESP‐MMPol@MD protocol. A) Fluorescence emission spectrum at 295 nm excitation of the HSA–ligand complex ([P] = 5 µm HSA in PBS 1×, 25 °C) at increasing ligand concentrations ([L] = 10, 50, 100 µm), with fitted binding affinity (*K_d_*) and experimental FRET efficiency (*E_exp_*) indicated. B) MM‐GBSA binding free energies (Δ*G_bind_*) and percentage of stable MD replicas from the three best docking poses in each binding site. C) Distributions of FRET efficiencies estimated from TrESP‐MMPol@MD couplings for the ligand in all six HSA sites; vertical red line = *E_exp_*. D) Binding modes in sites IB and IIA (centroids of the most populated MD cluster, green) compared to the known binding modes in PDB ID 2BXM (pink).^[^ ^42^ ^]^

We studied biophysically the interaction of HSA with the ligands in our library by monitoring changes in the intrinsic protein fluorescence upon ligand titration. The results are shown in **Figure** **6** for indomethacin, and in Figures S17–S24 (Supporting Information) for the rest of the fluorescent molecules. In addition, in Table S6 (Supporting Information), we provide a comparison of the results using 280 and 295 nm excitation wavelengths, as well as the corresponding values obtained without applying inner‐filter effect corrections. FRET efficiencies and dissociation constants derived at 295 nm are expected to be more accurate than those at 280 nm excitation, as they allow to minimize contributions from the absorption of Tyr residues in HSA, although in general we find quite consistent results. On the other hand, for most ligands we work with absorbances up to ≈0.1–0.3, whereas for carprofen we reach values ≈0.7 at 295 nm. Whereas for several ligands the resulting IFE corrections on efficiencies are moderate, we find the larger impact on 1‐naphthol, naproxen, and carprofen. Indeed, for carprofen, the very large absorbances up to ≈0.7 at 295 nm lead to inconsistent fits with a negative *n* value and large differences compared to the 280 nm results, in stark contrast with the other ligands. In this case, thus, the results at 280 nm, where absorption is halved and thus inner‐filter effects are much lower, appear more accurate, so for this ligand we take this value as our reference.

In all cases, we observed significant quenching of the protein emission band centered at 334 nm. We then calculated the dissociation binding constants (*K_d_*) and the solute binding parameter *n* for each ligand: 1‐naphthol (*K_d_* = 137 µM, n = 0.73), 2‐naphthol (*K_d_* = 6.6 µM, n = 0.71), NNE (*K_d_* = 3.5 µM, n = 0.33), ACA (*K_d_* = 15.5 µM, n = 0.67), (*S*)‐carprofen (*K_d_* = 1.8 µM, n = 0.48), (*S*)‐naproxen (*K_d_* = 24 µM, n = 0.68), QAA (*K_d_* = 48 µM, n = 0.76) and indomethacin (*K_d_* = 2.4 µM, n = 0.45). The *K_d_* value obtained for (*S*)‐naproxen 24 µM was larger than values ≈0.6–1.1 µm previously reported for its binding to the primary site of HSA,^[^ ^48^, ^49^ ^]^ whereas those found for (*S*)‐carprofen 1.8 µm were similar to previous reports ≈0.2–0.9 µm.^[^ ^50^, ^51^ ^]^ For indomethacin, we also obtained a *K_d_* value 2.4 µm similar than a previously reported value (6.1 µm).^[^ ^52^ ^]^

Next, the FRET efficiencies were determined from the quenching of the protein fluorescence and corrected by the fraction of ligand‐bound protein according to the dissociation constants. We find efficiencies ranging from 0.57 to 1.00: 1‐naphthol (*E* = 0.46), 2‐naphthol (*E* = 0.80), NNE (*E* = 0.55), ACA (*E* = 0.63), (*S*)‐carprofen (*E* = 0.81), (*S*)‐naproxen (*E* = 0.57), QAA (*E* = 0.60) and indomethacin (*E* = 0.73). These values were then compared to the average FRET values predicted for the binding modes in each site using the TrESP‐MMPol@MD protocol developed here, reported in Table S3 (Supporting Information). In that table, we also report the deviation between the experimental efficiency and that predicted by the simulations for the ligands in each binding site of HSA, which is our metric to rank the compatibility of the binding models with experimental data. We note here, however, that this metric will be most accurate when the ligand binds a single site, whereas secondary binding to another site can lead to deviations, which reflect the fact that the experimental value has contributions to more than one binding site.

(*S*)‐naproxen and (*S*)‐carprofen are known to bind with the highest affinity to site IIIA (Sudlow's site II) and often are considered to bind with lower affinity to site IIA (Sudlow's site I). Crystallographic data on ibuprofen have also confirmed IIIA binding but indicate secondary binding on site IIA‐IIB.^[^ ^42^ ^]^ We showed in a previous work that this is also the case for flurbiprofen, leading to an enantioselective fluorescence quenching observed for this ligand bound to HSA.^[^ ^17^, ^18^ ^]^ Our measured FRET efficiency for (*S*)‐naproxen 57% is nicely consistent with the known major binding to site IIIA, leading to a 41% efficiency, leading to a somewhat larger value which could arise from minor binding to site IIA‐IIB (88% predicted efficiency). Our experimental value, however, is also close to that predicted for Cleft, 63%. This reflects the limits of our technique, which can filter the binding models consistent with observed FRET data, but may require additional techniques in order to resolve cases in which several models are compatible with experiments. It is also interesting to recall that, for this case, secondary binding leads to a measured value 57% that deviates by 16% from the value predicted for IIIA. This suggests that deviations larger than ≈20–30% between experimental and theoretical values can be safely assumed to rule out major binding models for a given ligand.

If we focus on (*S*)‐carprofen, for which we measure an affinity in excellent agreement with previous reports, our estimated FRET efficiency (85%) for site IIIA, the main binding site expected for this ligand, is again in excellent agreement with the value (81%) measured from fluorescence data, whereas the experiment is also compatible with a minor population bound to site IIA‐IIB, for which an expected efficiency of 94% was predicted.

Finally, the 73% FRET efficiency measured for indomethacin, for which we also measure an accurate affinity consistent with previous reports, we found again an excellent agreement with the efficiencies estimated for the known binding to sites IIA (100%) and IB (25%). Indeed, indomethacin crystallographic data indicate binding to both IB and IIA sites,^[^ ^42^ ^]^ with the main site, however, expected to be IIA, Sudlow's site I, which explains why the observed value is closer to that of site IIA.

In Figure 6 we illustrate the results obtained for indomethacin, which, together with those found for (*S*)‐naproxen and (*S*)‐carprofen, shown in Figures S22 and S23 (Supporting Information), validate the potential of the TrESP‐MMPol@MD protocol to identify ligand binding sites. The binding modes obtained from MD simulations present some conformational diversity. We thus display the centroids of the most populated clusters from a clustering analysis of the stable MD trajectories in each binding site, whereas in Figure S26 (Supporting Information), we illustrate the underlying diversity by reporting the centroids of four other clusters. The results indicate a good agreement with crystallographic data of ibuprofen for (*S*)‐carprofen and (*S*)‐naproxen bound to sites IIIA and IIA‐IIB, with a very similar orientation of the carboxylic acid group. For indomethacin, we also found binding poses close to those found in the crystal structure for sites IB and IIA. Our protocol thus allows thus to recover the binding site and find reasonable binding modes of these ligands.

Once validated the approach, we also analyzed the main binding sites for the other fragments in our library. For 1‐naphthol, our results strongly support major binding to sites Cleft or IIIA, we predicted efficiencies 51% and 42% close to the experimental 46%. For 2‐naphthol, we found a higher experimental efficiency 80%, which is compatible with binding to both sites IIA or IIA‐IIB. In this case, however, the ligand stability in IIA‐IIB was only ≈20%, the lowest among all sites, whereas binding to IIA was robust in ≈80% of them. This suggests that in this case, the preferred site for this fragment is IIA.

For ACA, we expected a major population of the deprotonated state compared to its zwitterionic form, as discussed in the Methods.^[^ ^53^ ^]^ The FRET data further supported binding of the deprotonated state, with an experimental efficiency 63% compatible with binding either to sites Cleft (72%) and IIIA (73%). We found, however, a much larger MD stability for the IIIA site compared to Cleft, thus indicating IIIA as the most plausible binding site. For a protonated ACA, the experimental data would only be compatible with binding to Cleft, but we found a very low stability for binding to the Cleft site, also for protonated ACA, with only ≈20% of the MD replicas keeping the ligand in the pocket.

In the case of NNE, we measured an efficiency 55%, which is compatible with binding to sites Cleft (64%) and IIIA (45%), but our MD simulations in both sites indicated similar stabilities, thus further experiments or simulations would be needed to assign the major binding site. Finally, for QAA, we measured an efficiency 60%, which suggests major binding to site Cleft, as we predicted an expected FRET value of 65%.

## 3 Conclusion

We have developed and validated an integrated protocol that combines experimental FRET data with atomistic simulations based on the TrESP‑MMPol model to identify binding sites and characterize binding modes in protein–ligand complexes and resolve their structural features with nanometer accuracy. The approach overcomes limitations of the point‑dipole approximation and isotropic orientation assumptions in Förster theory, efficiently incorporates dielectric screening and restricted chromophore orientation effects in protein environments, and provides a direct metric for validating in silico structural models through simulated FRET observables. In terms of computational cost, this is mostly determined by the need to sample properly the protein‐ligand complexes with MD simulations, whereas the cost associated to TrESP‑MMPol calculations is comparatively low.

Applied to HSA, the approach has faithfully reproduced known binding modes and affinities for reference ligands, while also predicting plausible binding modes for less well‑characterized ligands. The protocol can be readily transferred to study ligand binding in other proteins or biological macromolecules of interest characterized by a single Trp residue. The protocol could also be extended to targets with multiple Trp residues, although this would require additional modelling of the absorption and energy transfers among Trp molecules, which is expected to be less accurate and complicates the analysis.^[^ ^16^ ^]^ Alternatively, in these cases, targets can be tagged with a single site‐specific unnatural fluorescent amino acid^[^ ^47^ ^]^ or an extrinsic dye, although linker flexibility in the latter case could diminish the capability to resolve binding models characterized by different ligand‐dye orientations.^[^ ^46^ ^]^ Our protocol also benefits from major binding to a single location, as secondary binding can lead to deviations between the experiment and the FRET efficiency expected for the major site. In this regard, our data suggests that deviations larger than ≈20–30% between experimental and theoretical values can be safely assumed to rule out major binding models for a given ligand.

Overall, the protocol can be of particular relevance in fragment‐based drug design campaigns where the target binding site is not known, or when the campaign aims at identifying allosteric pockets different from orthosteric sites. Moreover, fragment screenings campaigns can be performed with larger collections compared to the initial library adopted here, taking advantage of the large compilations of optical properties for organic compounds currently available,^[^ ^46^ ^]^ or using libraries of fragments covalently linked to a common fluorophore, which allows a simpler processing of theoretical and experimental results.^[^ ^44^ ^]^

## 4 Experimental Section

### Förster Energy Transfer Theory

Energy transfer between a donor (D) and an acceptor (A) can be described in the weak‐coupling limit using Förster theory:^[^ ^16^ ^]^

(1) $$k_{FRET}=\frac{2\pi }{\hbar }V^{2}J\approx \frac{2\pi }{\hbar }{\left(\frac{1}{n^{2}}\frac{\kappa \mu_{D}\mu_{A}}{R^{3}}\right)}^{2}J$$

where the D/A electronic coupling *V* is described using a PDA, *J* is the spectral overlap between area‐normalized D emission and A absorption spectra, µ_*D*_ and µ_*A*_ the D/A transition dipole moments, κ the dipole orientation factor, *R* the D/A center‐to‐center distance, and *n* the refractive index of the medium. The PDA allows to express the rate from spectroscopic data measured for the non‐interacting dyes:

(2) $$k_{FRET}=k_{D}{\left(\frac{R_{o}}{R}\right)}^{6}=\frac{1}{\tau_{D}}{\left(\frac{R_{o}}{R}\right)}^{6}$$

where *k_D_* = 1/τ_*D*_ indicates the decay rate of the excited *D* in the absence of *A* based on its lifetime τ_*D*_, and *R_o_* the critical quenching radius or Förster radius, which corresponds to the distance with 50% efficiency. The transfer efficiency can then be expressed as:

(3) $$E_{FRET}=\frac{k_{FRET}}{k_{D}+k_{FRET}}=\frac{1}{1+{\left(R/R_{0}\right)}^{6}}$$

In the multiscale protocol, instantaneous FRET rates are computed from couplings computed using the TrESP‐MMPol and PDA models at times *t* of an MD trajectory using the following expression, derived from Equations (1) and (2):^[^ ^24^ ^]^

(4) $$k_{theo}\left(t\right)=V{\left(t\right)}^{2}\frac{3n^{4}R_{0}^{6}}{2\tau_{D}\mu_{D}^{2}\mu_{A}^{2}}$$

FRET efficiencies are then estimated considering static and dynamic disorder by separating slow and fast fluctuations in instantaneous transfer rates:^[^ ^54^ ^]^

(5) $$E_{theo}=\langle \frac{1}{1+\frac{1}{\tau_{D}{\langle k_{theo}\left(t\right)\rangle }_{fast}}}\rangle slow$$

### Docking, Molecular Dynamics, and Binding Free Energy Simulations

The protein‐ligand complexes were built starting from the crystal structure of HSA solved at 2.50 Å resolution (PDB ID 2BXM).^[^ ^42^ ^]^ All ligands and waters were removed, and missing atoms and amino acids were added manually and using pdb4amber in Amber22.^[^ ^55^ ^]^ Protonation states of amino acids were assigned with Propka3,^[^ ^56^ ^]^ indicating protonated His67 and His247.

A small library of eight fluorescent ligands with appropriate spectroscopic properties to quench Trp through energy transfer was then docked to HSA. The library contained 1‐naphthol, 2‐naphthol, NNE, ACA and QAA, suggested for Trp‐ligand FRET studies by Zhang and co‐workers,^[^ ^4^, ^57^ ^]^ as well as (*S*)‐carprofen, (*S*)‐naproxen and indomethacin, which were chosen for validation based on available knowledge of their binding properties to HSA.^[^ ^42^ ^]^ Docking simulations were carried out with rDock,^[^ ^58^ ^]^ and the docking volume was defined using the reference ligand method based on the poses of known ligands, with the resulting cavity mapping regions being increased by a radius of 6 Å. The study used the reference ligands iodipamide (Cleft site, PDB ID 2BXN), indomethacin (IB and IIA sites, PDB ID 2BXM), ibuprofen (IIA‐IIB and IIIA sites, PDB ID 2BXG), and oxyphenbutazone (IIIB site, PDB ID 2BXO).^[^ ^42^ ^]^ Protonation states of the ligands were assigned at pH 7 with Corina.^[^ ^59^ ^]^ 9‐acridinecarboxylic acid was found to be 61% in deprotonated state and 39% in protonated state, which agrees with a value of 6.2 for the pKa estimated from pH‐dependent spectroscopic measurements.^[^ ^53^ ^]^ Nevertheless, both possibilities were considered due to the small difference in stability, and results for the protonated form are provided in the Supporting Information. The scoring function SF3 in rDock was used.

The three docking poses with the highest scores were then selected, and their stability was investigated performing MD simulations based on the Amber ff19SB, OPC, and gaff2 force fields for the protein, water, and ligands, respectively. RESP charges for the ligands were computed from B3LYP/6‐31G(d) calculations on optimized geometries using Gaussian 16.^[^ ^60^ ^]^ All systems were neutralized with Na^+^ ions, solvated in an octahedral box (buffer zone 10 Å), minimized with the protein restrained at its initial structure, and then thermalized from 0 K to 300 K in 5 NVT steps of 50 ps each, followed by a 250 ps NPT equilibration. Then 3 × 100 ns production runs were carried out starting from each docking pose, resulting in a total of 3 × 3 replicas for each ligand in each site. Simulations were performed using Amber22^[^ ^55^ ^]^ using periodic boundary conditions, the SHAKE algorithm, particle‐mesh Ewald, and a nonbonded cutoff of 10 Å using the Hydrogen Mass Repartitioning scheme, which allowed an integration time step of 4 fs.^[^ ^61^ ^]^ The stability of the binding modes was examined by computing the root mean square deviation (RMSD), mass‐weighted radius of gyration R_g_, and root mean square fluctuations (RMSF) of the protein (Figures S27–S34, Supporting Information) and the distance between the center of mass of the ligand compared to the initial docking pose, and replicas where the ligand left the binding site, displaying distances >7 Å were discarded. MM‐GBSA calculations of protein‐ligand binding free energies were performed for the last 50 ns using the Gbneck2 (igb = 8) model for implicit solvent,^[^ ^62^ ^]^ without additional ionic strength and performing a residue pairwise energy decomposition analysis. Clustering analysis was performed to characterize the conformations of the binding modes of the ligands for the last 50 ns of the trajectories using the K‐means clustering algorithm implemented in AmberTools. The study used as metric the RMSD of the ligand and the 4 surrounding residues with the highest contribution to the MM‐GBSA binding free energy, and the number of clusters was set to 5.

### Electronic Coupling Calculations and Energy Transfer Rates

Electronic couplings required by Equation (4) were computed both using the PDA and the TrESP‐MMPol model.^[^ ^24^, ^40^ ^]^ The latter describes D/A molecules using sets of atomic transition charges derived from a fit of the electrostatic potential (ESP) computed from QM‐derived transition densities,^[^ ^63^ ^]^ whereas the environment is described using a polarizable force field based on the induced dipole model. The total coupling includes the Coulomb interaction between TrESP charges and an explicit environment‐mediated term:

(6) $$V=V_{Coul}+V_{env}$$

(7) $$V_{Coul,TrESP}=\sum_{i,j}\frac{q_{D,i}^{T}q_{A,j}^{T}}{\left|r_{i}-r_{j}\right|}$$

(8) $$V_{env,TrESP}=-\sum_{i,l}\frac{q_{D,i}^{T}\left(r_{i}-r_{l}\right)\cdot \mu_{l}^{MMPol}\left(\left\{q_{A}^{T}\right\}\right)}{{\left|r_{i}-r_{l}\right|}^{3}}$$

where $q_{D,i}^{T}$ and $q_{A,j}^{T}$ indicate transition charges on the D/A atoms *I* and *j*, respectively, and $\mu_{l}^{\mathrm{MMPol}}$ induced dipoles on MM atoms *l*. From these terms, one can then define a screening factor *s* that can be compared to the factor in the PDA expression *s* = 1/*n* ^2^:

(9) $$s=\frac{V_{Coul}+V_{env}}{V_{Coul}}$$

TrESP charges were fitted to reproduce the potential obtained from QM transition densities of the relevant π→π* states of each ligand and for the Trp L_a_ state using the TraDA tool.^[^ ^64^ ^]^ Excited states were obtained at the TD‐B3LYP/6‐31G(d) level of theory on B3LYP/6‐31G(d) optimized geometries of the ligands and 3‐methyl‐indole, respectively. For ligands ACA, (*S*)‐carprofen, (*S*)‐naproxen, QAA, and indomethacin, the state of interest presented some mixing with another state, or artificial low‐energy states were observed, so TrESP charges were computed from additional TD‐CAM‐B3LYP/6‐31G(d) calculations. The MMPol environment was described using the Amber pol12 AL polarizable force field.^[^ ^65^, ^66^ ^]^ TrESP‐MMPol calculations were performed adopting a MMPol cutoff radius equal to 15 Å^[^ ^24^ ^]^ every 50 ps on the stable MD trajectories obtained for each system using the Trespcoup software.^[^ ^67^ ^]^ PDA calculations were performed using the electric transition dipole moments obtained in the corresponding TD‐DFT calculations, and adopting a value *n* ^2^ equal to 2.^[^ ^23^ ^]^

### Steady‐State Spectroscopy

Ligands 1‐naphthol, 2‐naphthol, ACA, QAA, NNE, (*S*)‐carprofen, (*S*)‐naproxen, indomethacin, and the protein HSA were purchased from Sigma Aldrich in pure powdered form. For absorbance and fluorescence, solutions of the ligand, protein, and protein‐ligand complexes at different concentrations were prepared using PBS 1x buffer. Absorbance measurements were conducted using a Perkin Elmer Lambda 950 spectrophotometer using a 2.5 µM concentration for HSA and 5 to 100 µm for the ligands, depending on each case. For protein‐ligand complexes, fluorescence titrations were carried out at fixed 5 µm HSA concentration and ligand concentrations equal to 0, 10, 50, and 100 µm using an Aminco Bowman Series 2(AB2) spectrometer, using excitation wavelengths 280 and 295 nm. The fluorescence intensities of protein‐ligand complexes were corrected for inner‐filter effects based on the absorbance of the ligands at the excitation and emission wavelengths ($I_{DA,cor}=I_{DA}10^{(Abs_{ex}+Abs_{em})/2}$).^[^ ^19^ ^]^ The fluorescence of the ligands in the absence of HSA was also recorded at 100 µm concentration. Spectral overlaps used to compute FRET rates were computed from the measured area‐normalized D emission and A absorption spectra and are reported in the Supporting Information. Protein and ligand solutions were freshly prepared, and titrations were carried out in the cuvette right before the measurement. The average of three measurements for the complexes at each ligand concentration was used to plot the spectra and compute FRET efficiencies following this formula:

(10) $$E=1-\frac{I_{DA}}{I_{D}}$$

where *I_DA_* is the intensity of the complex and *I_D_* the intensity of HSA in the absence of ligand. The dissociation/association binding constants (*K_d_* = 1/*K_b_*) and the Hill coefficient (*n*) were also estimated using the following binding equation using the fluorescence intensities at different ligand concentrations [*L*]:

(11) $$\log\left(\frac{I_{D}-I_{DA}}{I_{DA}}\right)=\log\left(K_{b}\right)+n\log\left(\left[L\right]\right)$$

In the measurements, a value for the parameter *n* < 1 is typically obtained, suggesting heterogeneous binding. For each ligand, measurements were done at a selected emission wavelength with high protein and minimal ligand emission: 1‐naphthol 320 nm; 2‐naphthol 322 nm; NNE 338 nm; ACA 334 nm; QAA 330 nm; (*S*)‐carprofen 334 nm; (*S*)‐naproxen 315 nm; indomethacin 337 nm. Efficiencies were corrected based on the percentage of protein‐ligand complex formed in each solution, based on the measured *K_d_* values, then final efficiencies were averaged over the three concentrations.

## Conflict of Interest

E.C. is an employee of Gain Therapeutics Sucursal en España. Part of this work was carried out during a secondment of doctoral researcher OE at Gain Therapeutics. The company had no additional role in study design, data analysis, decision to publish, or preparation of the manuscript. C.G. is co‐founder and shareholder of Oniria Therapeutics. C.G. is also drug discovery consultant of Oniria Therapeutics. All other authors declare no competing interests.

## Supporting information



Supporting Information

## Data Availability

The Python scripts used to process fluorescence experimental data to generate dissociation constants and FRET efficiencies and electronic coupling trajectories to generate theoretical FRET efficiencies can be found in Github at https://github.com/CompPhotoLab‐bcn/FRET_tools.
