# Supplementary material for: Characterization of Ligand Binding in Human Serum Albumin from Atomistic Energy Transfer Simulations
Source: Small Methods. 2025 Nov 14;9(12):e01820. doi: 10.1002/smtd.202501820 (PMC12716216; doi:10.1002/smtd.202501820)
Supplement: Supplementary file 1 — Supporting Information [file SMTD-9-e01820-s001.pdf]

Supporting Information for
Characterization of Ligand Binding in Human Serum Albumin from Atomistic Energy Transfer Simulations

Özge Ergün,[1,2] Andrea Bertran-Mostazo,[1,3] Elena Cubero,[4] Carles Galdeano[1,3] and Carles Curutchet[1,2*]

[1]Departament de Farmàcia i Tecnologia Farmacèutica, i Fisicoquímica, Facultat de Farmàcia i Ciències de l'Alimentació, Universitat de Barcelona (UB), Barcelona, Spain

[2]Institut de Química Teòrica i Computacional (IQTCUB), Universitat de Barcelona (UB), Barcelona, Spain

[3]Institut de Biomedicina (IBUB), Universitat de Barcelona (UB), Barcelona, Spain

[4]Gain Therapeutics Sucursal en España, Barcelona Science Park, Barcelona, Spain

Preamble

This Supporting Information provides additional figures and tables referenced in the main text, including FRET efficiency distributions and statistical error analysis, donor–acceptor distance error analysis, MM-GBSA binding free energies, RMSD, R_g and RMSF plots, dipole–dipole orientation factor distributions, deviations of dipole-dipole and screening contributions to electronic couplings, protein-ligand binding models, and fluorescence and absorption spectra. The SI is intended to make all numerical values, intermediate analyses, and binding-mode visualisations available for reproducibility and further comparison.

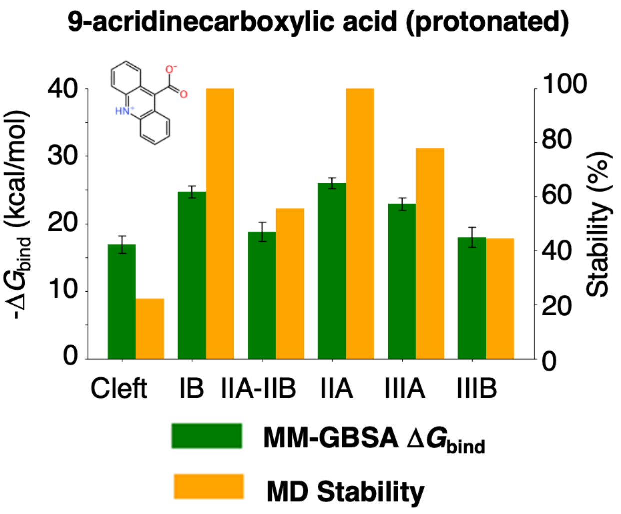

Figure S1. MM-GBSA binding free energies and percentage of stable replicas obtained from MD simulations started from the three best docking poses of protonated 9-acridinecarboxylic acid in the six binding sites of HSA.

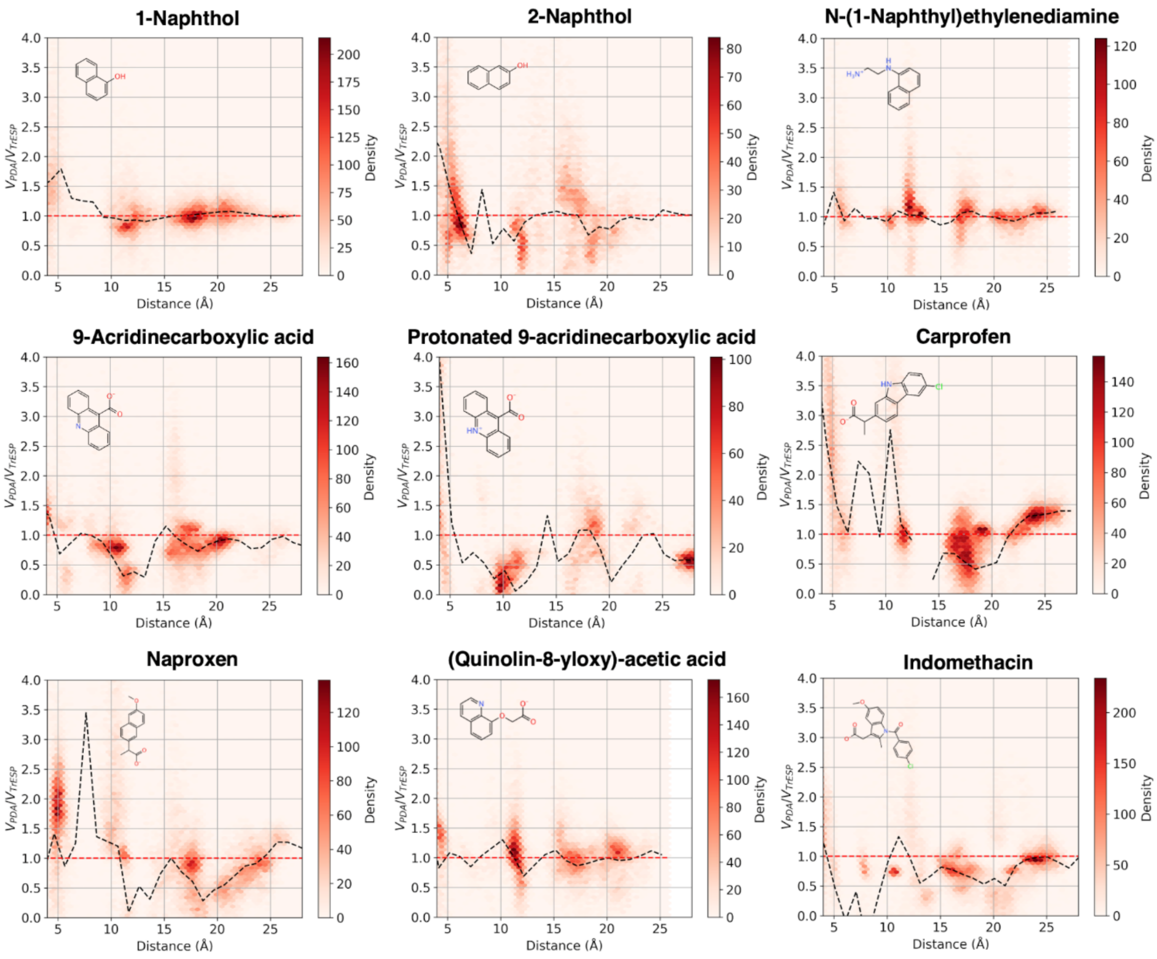

Figure S2. Density distribution of ratio between point dipole approximation (PDA) and TrESP coulombic coupling contributions for each ligand–HSA complex, as a function of donor–acceptor (D/A) separation. Black dashed curve: average over 1 Å distance bins; red dashed line: ideal PDA/TrESP ratio = 1.

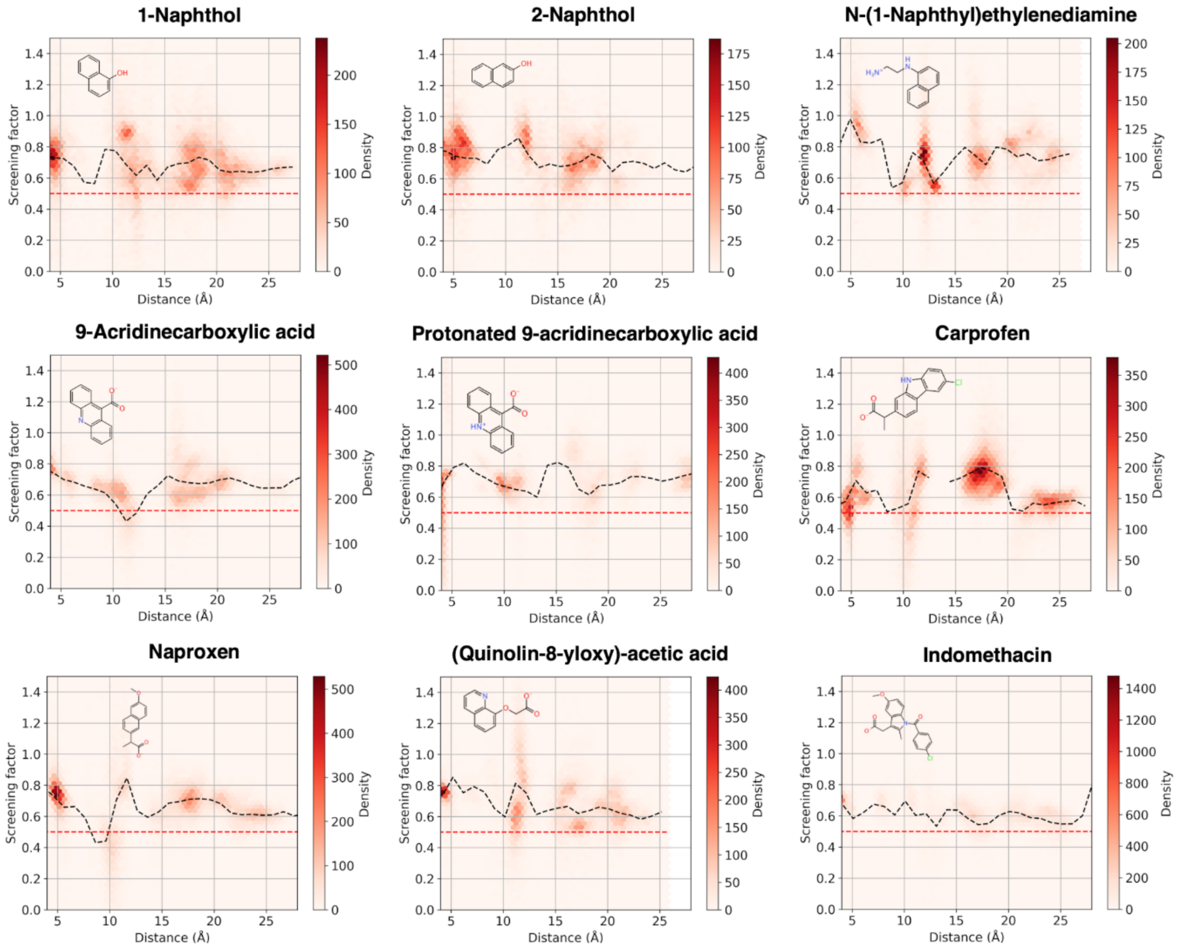

Figure S3. Density distribution of TrESP-MMPol screening factors for each ligand–HSA complex as a function of D/A separation. Black dashed curve: average over 1 Å distance bins; red dashed line: Förster factor $s = 1/n^2$.

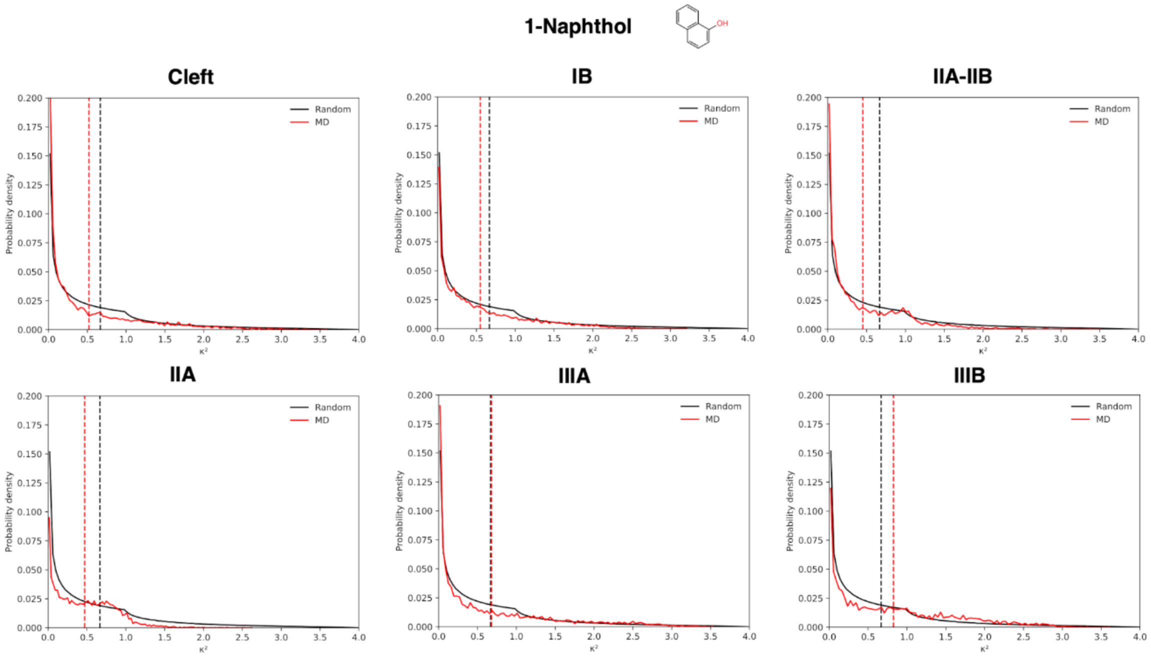

Figure S4. Probability distribution of dipole-dipole orientation factors (κ^2) derived from molecular dynamics (MD) simulations of 1-naphthol bound in the six binding sites of human serum albumin (HSA): Cleft, IB, IIA–IIB, IIA, IIIA, and IIIB. The red curve shows the κ^2 distribution obtained from each set of MD trajectories; the red dashed vertical line indicates the average κ^2 value from MD. For comparison, the black curve shows the theoretical isotropic κ^2 distribution and the black dashed vertical line marks the isotropic average $\kappa^2 = 2/3$ assumed in Förster theory.

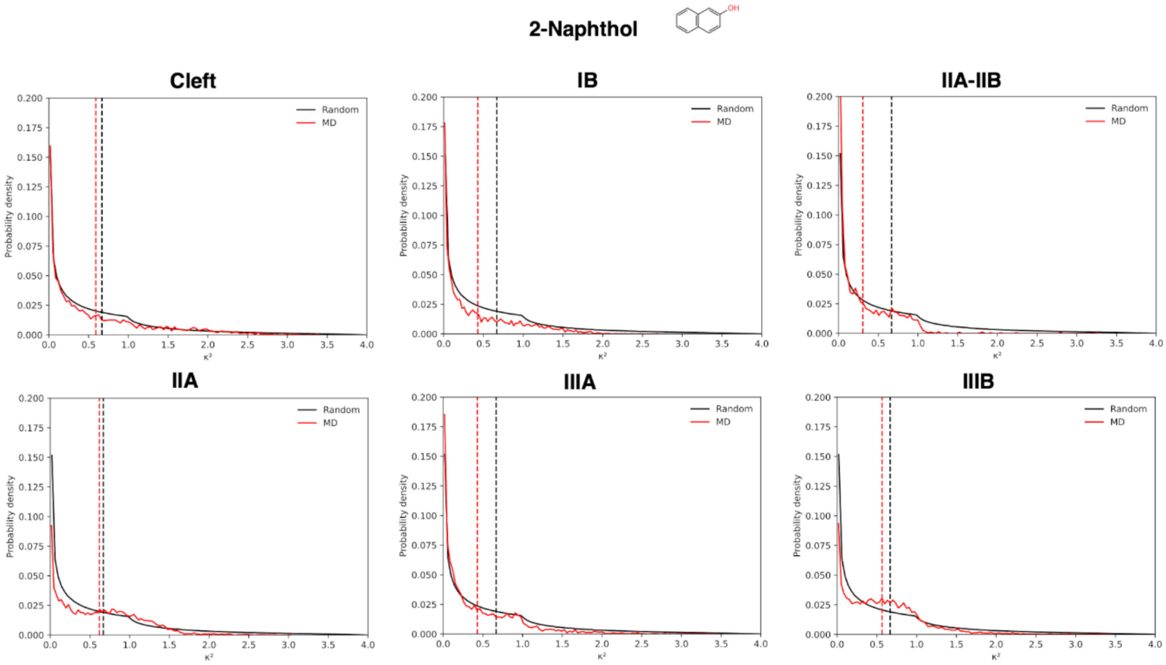

Figure S5. Probability distribution of dipole-dipole orientation factors (κ^2) derived from molecular dynamics (MD) simulations of 2-naphthol bound in the six binding sites of human serum albumin (HSA): Cleft, IB, IIA–IIB, IIA, IIIA, and IIIB. Red curve = MD distribution; vertical red dashed line = MD average κ^2. Black curve = isotropic distribution; vertical black dashed line = isotropic average $\kappa^2 = 2/3$.

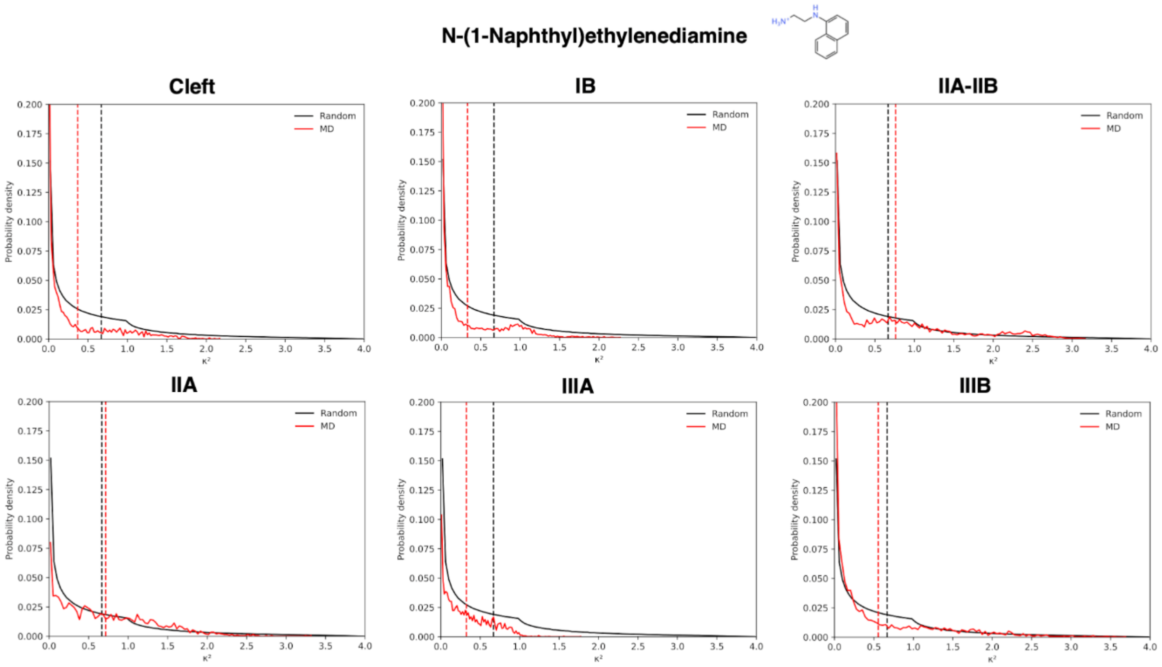

Figure S6. Probability distribution of dipole-dipole orientation factors (κ^2) derived from MD simulations of N-(1-naphthyl)ethylenediamine bound in the six binding sites of HSA: Cleft, IB, IIA–IIB, IIA, IIIA, and IIIB. Red curve = MD distribution; vertical red dashed line = MD average κ^2. Black curve = isotropic distribution; vertical black dashed line = isotropic average $\kappa^2 = 2/3$.

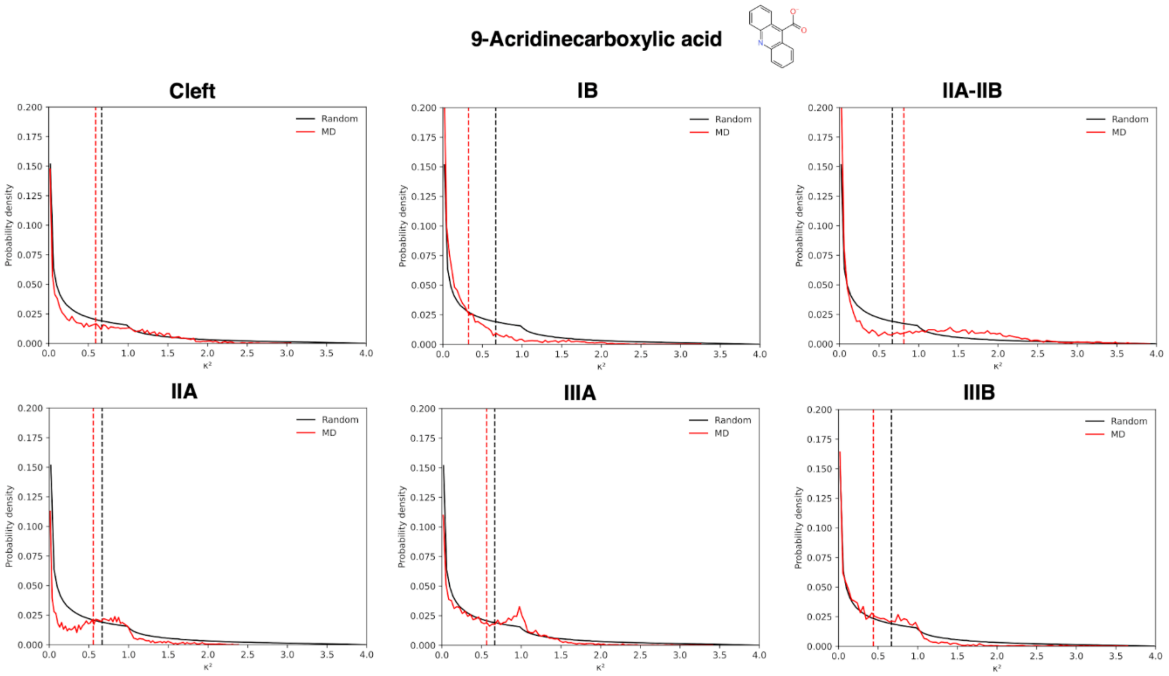

Figure S7. Probability distribution of dipole-dipole orientation factors (κ^2) derived from MD simulations of 9-acridinecarboxylic acid (deprotonated form) bound in the six binding sites of HSA: Cleft, IB, IIA–IIB, IIA, IIIA, and IIIB. Red curve = MD distribution; vertical red dashed line = MD average κ^2. Black curve = isotropic distribution; vertical black dashed line = isotropic average $\kappa^2 = 2/3$.

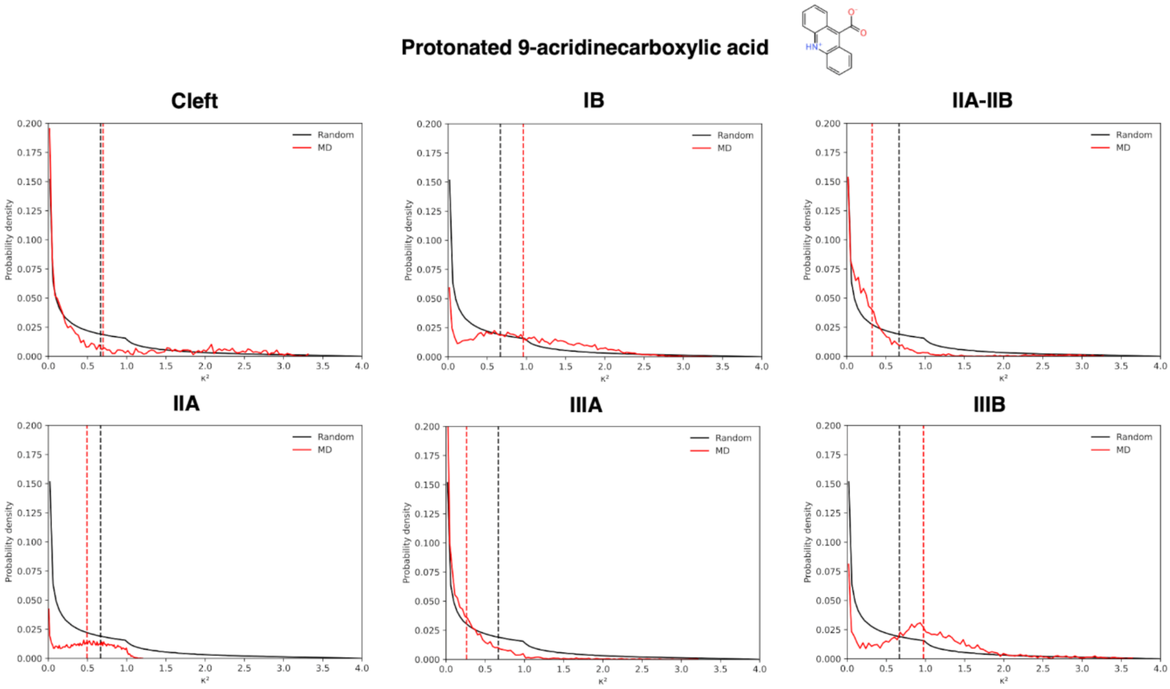

Figure S8. Probability distribution of dipole-dipole orientation factors (κ^2) from MD simulations of protonated 9-acridinecarboxylic acid bound in the six binding sites of HSA: Cleft, IB, IIA–IIB, IIA, IIIA, and IIIB. Red curve = MD distribution; vertical red dashed line = MD average κ^2. Black curve = isotropic distribution; vertical black dashed line = isotropic average $\kappa^2 = 2/3$.

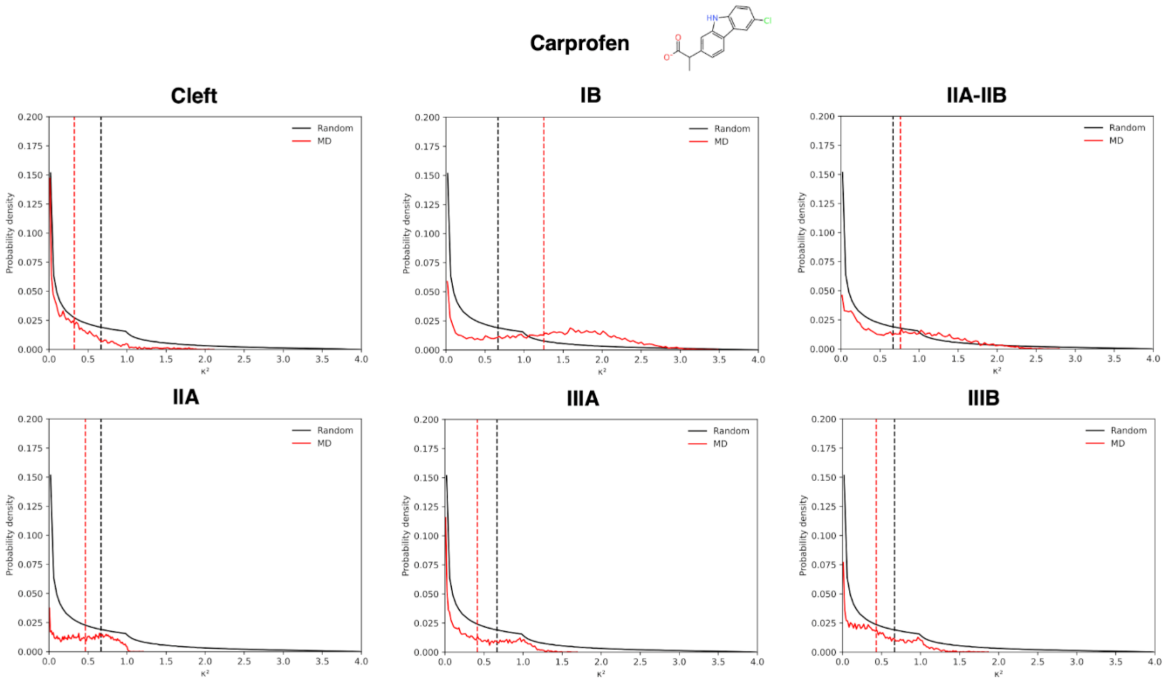

Figure S9. Probability distribution of dipole-dipole orientation factors (κ²) from MD simulations of (*S*)-carprofen bound in the six binding sites of HSA: Cleft, IB, IIA–IIB, IIA, IIIA, and IIIB. Red curve = MD distribution; vertical red dashed line = MD average κ². Black curve = isotropic distribution; vertical black dashed line = isotropic average κ² = 2/3.

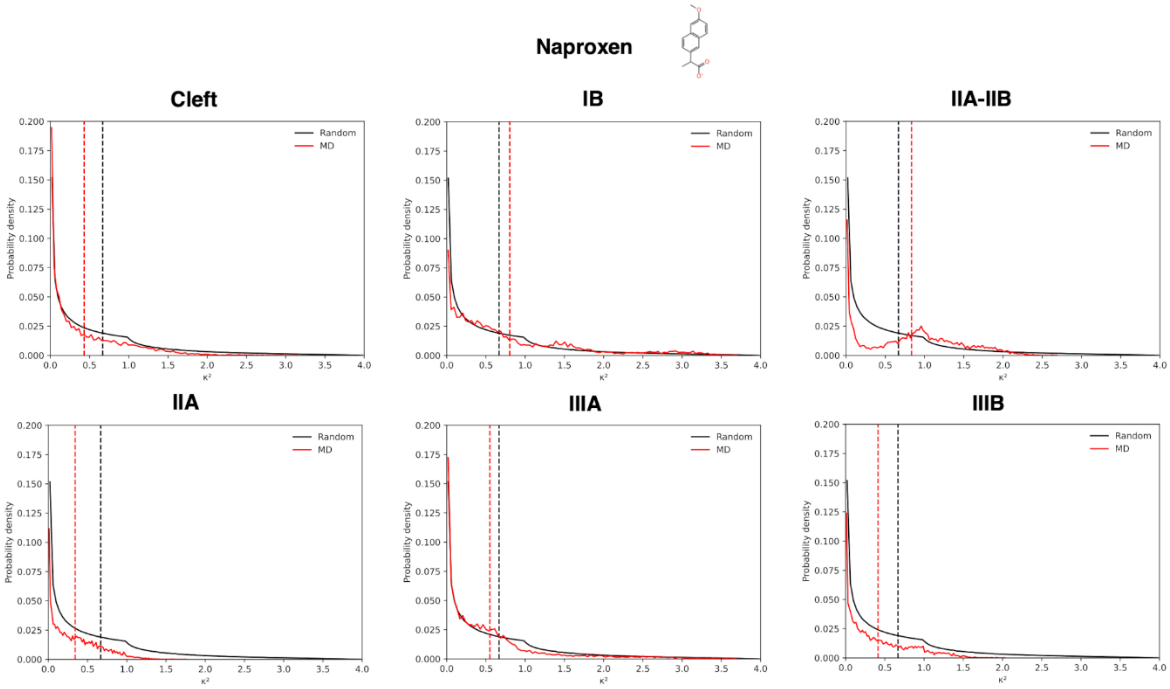

Figure S10. Probability distribution of dipole-dipole orientation factors (κ^2) from MD simulations of (*S*)-naproxen bound in the six binding sites of HSA: Cleft, IB, IIA–IIB, IIA, IIIA, and IIIB. Red curve = MD distribution; vertical red dashed line = MD average κ^2. Black curve = isotropic distribution; vertical black dashed line = isotropic average $\kappa^2 = 2/3$.

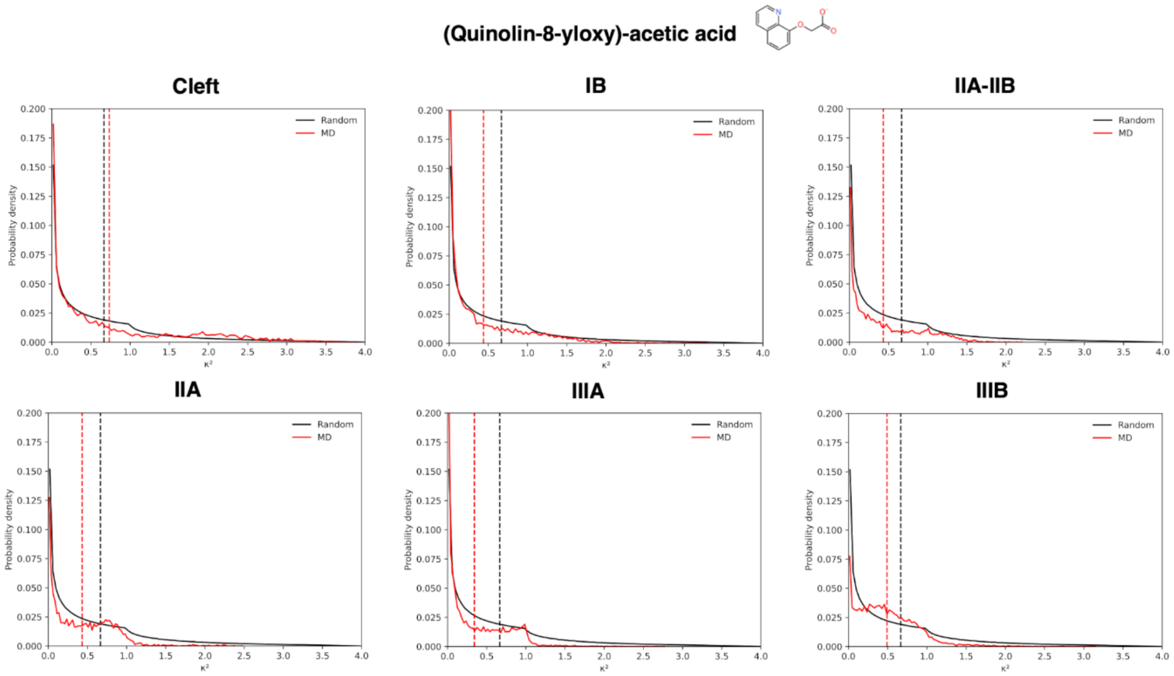

Figure S11. Probability distribution of dipole-dipole orientation factors (κ^2) from MD simulations of (quinolin-8-yloxy)-acetic acid bound in the six binding sites of HSA: Cleft, IB, IIA–IIB, IIA, IIIA, and IIIB. Red curve = MD distribution; vertical red dashed line = MD average κ^2. Black curve = isotropic distribution; vertical black dashed line = isotropic average $\kappa^2 = 2/3$.

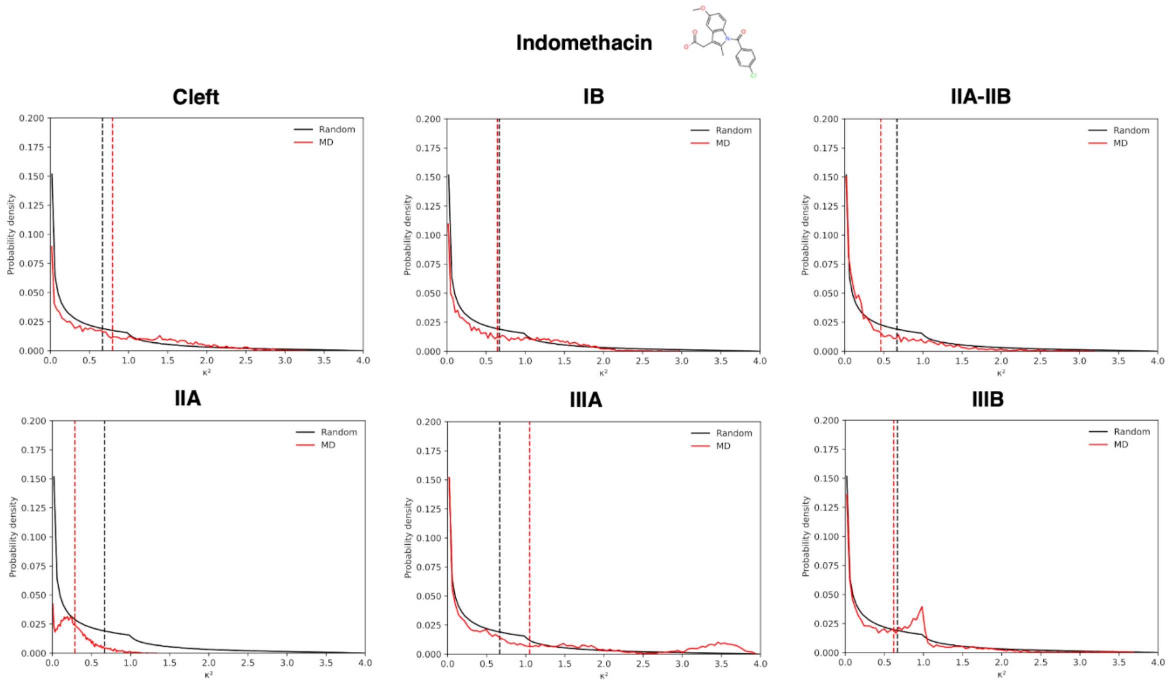

Figure S12. Probability distribution of dipole-dipole orientation factors (κ^2) from MD simulations of indomethacin bound in the six binding sites of HSA: Cleft, IB, IIA–IIB, IIA, IIIA, and IIIB. Red curve = MD distribution; vertical red dashed line = MD average κ^2. Black curve = isotropic distribution; vertical black dashed line = isotropic average $\kappa^2 = 2/3$.

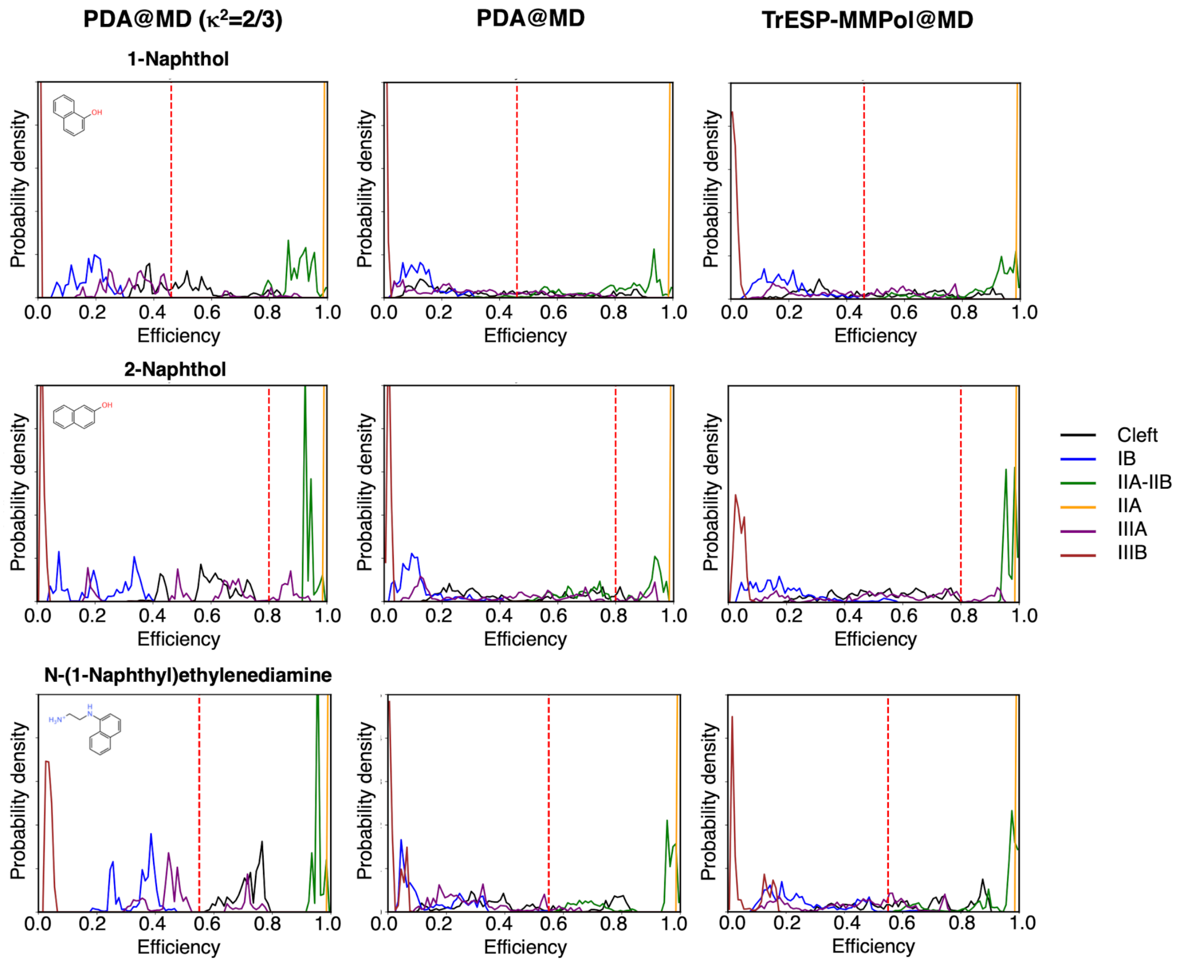

Figure S13. Distribution of Förster resonance energy transfer (FRET) efficiencies for 1-naphthol, 2-naphthol, and N-(1-naphthyl)ethylenediamine bound in the six binding sites of human serum albumin (HSA): Cleft, IB, IIA–IIB, IIA, IIIA, and IIIB. Efficiencies were obtained from coupling trajectories using three computational protocols: PDA@MD ($\kappa^2 = 2/3$) — point-dipole approximation with isotropic orientation factor; PDA@MD — point-dipole approximation with κ^2 values calculated from MD trajectories; and TrESP-MMPol@MD — atomistic transition-charge couplings in a polarizable environment. Probability densities are shown on the y-axis, efficiency values on the x-axis. Vertical red lines indicate experimental FRET values derived from fluorescence ligand titrations.

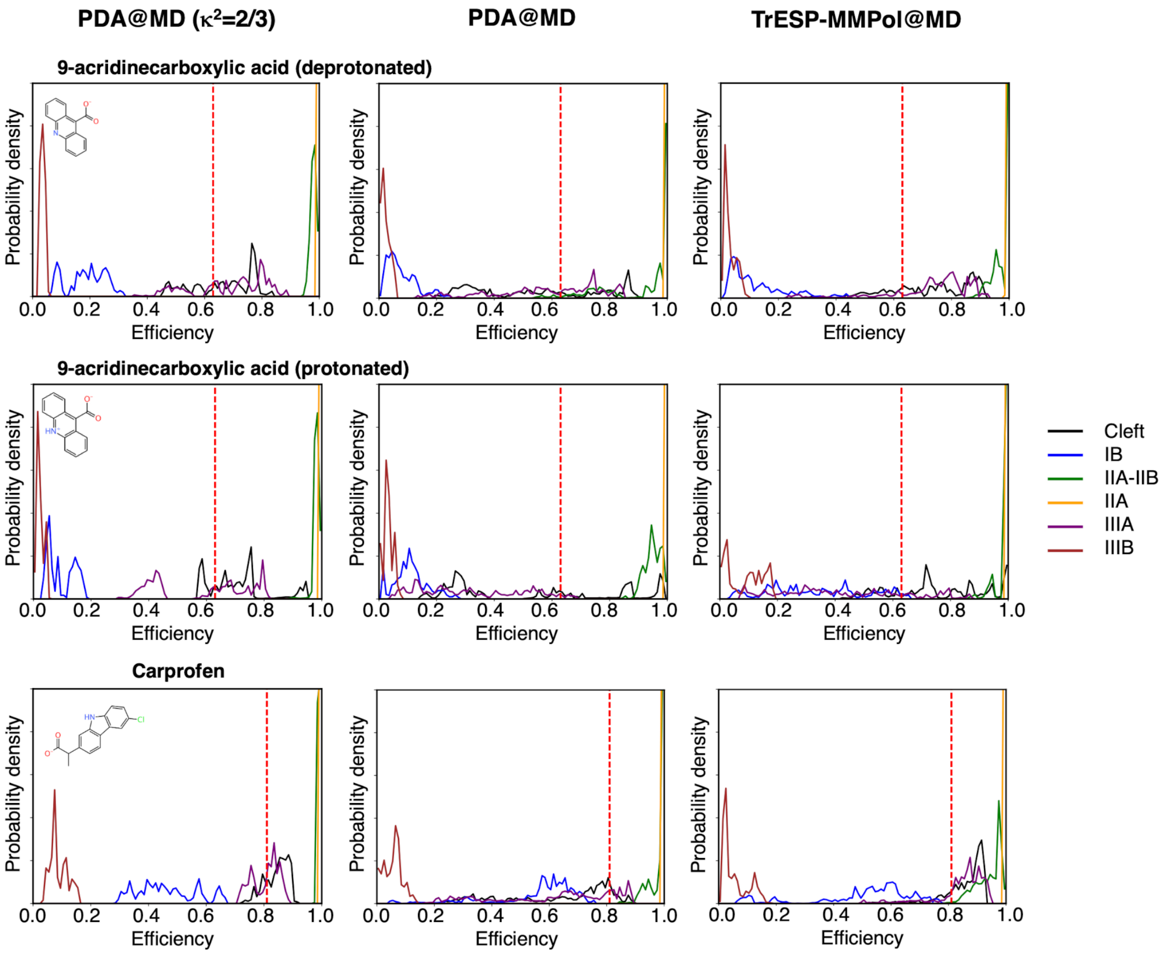

Figure S14. Distribution of FRET efficiencies for deprotonated 9-acridinecarboxylic acid, protonated 9-acridinecarboxylic acid, and (*S*)-carprofen bound in the six binding sites of HSA: Cleft, IB, IIA–IIB, IIA, IIIA, and IIIB. Efficiencies are estimated from coupling trajectories using PDA@MD ($\kappa^2 = 2/3$), PDA@MD with κ^2 from MD, and TrESP-MMPol@MD with atomistic polarizable environment. Vertical red lines indicate experimental FRET values derived from fluorescence ligand titrations.

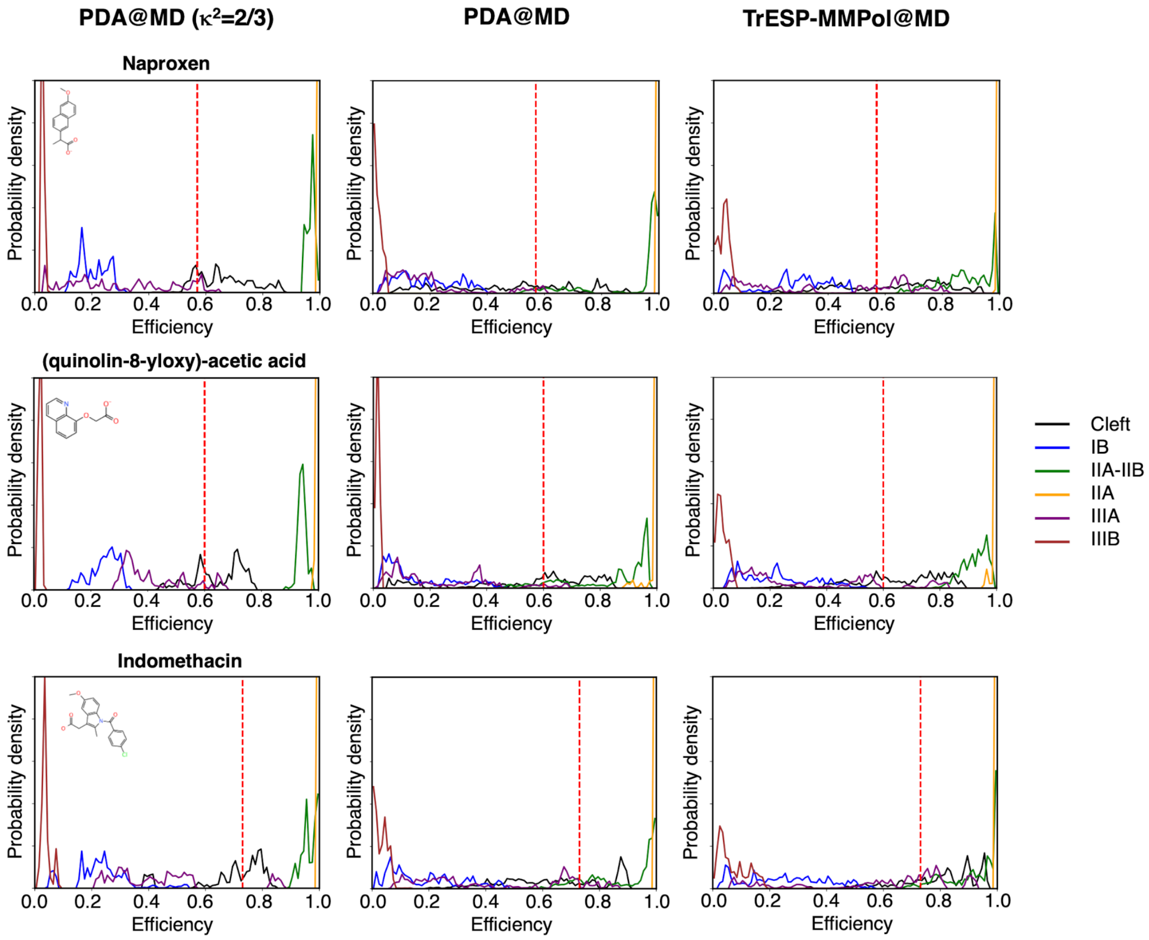

Figure S15. Distribution of FRET efficiencies for (*S*)-naproxen, (quinolin-8-yloxy)-acetic acid, and indomethacin bound in the six binding sites of HSA: Cleft, IB, IIA–IIB, IIA, IIIA, and IIIB. Efficiencies are estimated from coupling trajectories using PDA@MD ($\kappa^2 = 2/3$), PDA@MD with κ^2 from MD, and TrESP-MMPol@MD with atomistic polarizable environment. Vertical red lines indicate experimental FRET values derived from fluorescence ligand titrations.

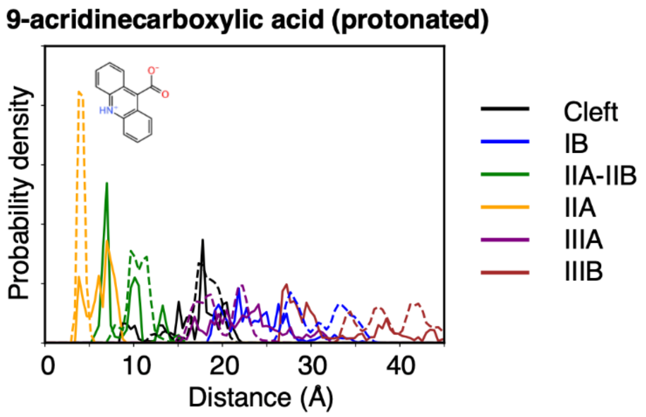

Figure S16. Comparison of donor–acceptor (D/A) distance distributions obtained directly from molecular dynamics (MD) trajectories (dashed curves) with those derived from TrESP-MMPol@MD FRET efficiency distributions transformed into distances using the Förster model and Eq. 3 of the main text (bold curves) for protonated 9-acridinecarboxylic acid in the six binding sites of human serum albumin (HSA): Cleft, IB, IIA–IIB, IIA, IIIA, and IIIB. Deviations between the two sets highlight the impact of the isotropic orientation assumption and the point dipole approximation, including dielectric-screening effects, in standard Förster theory.

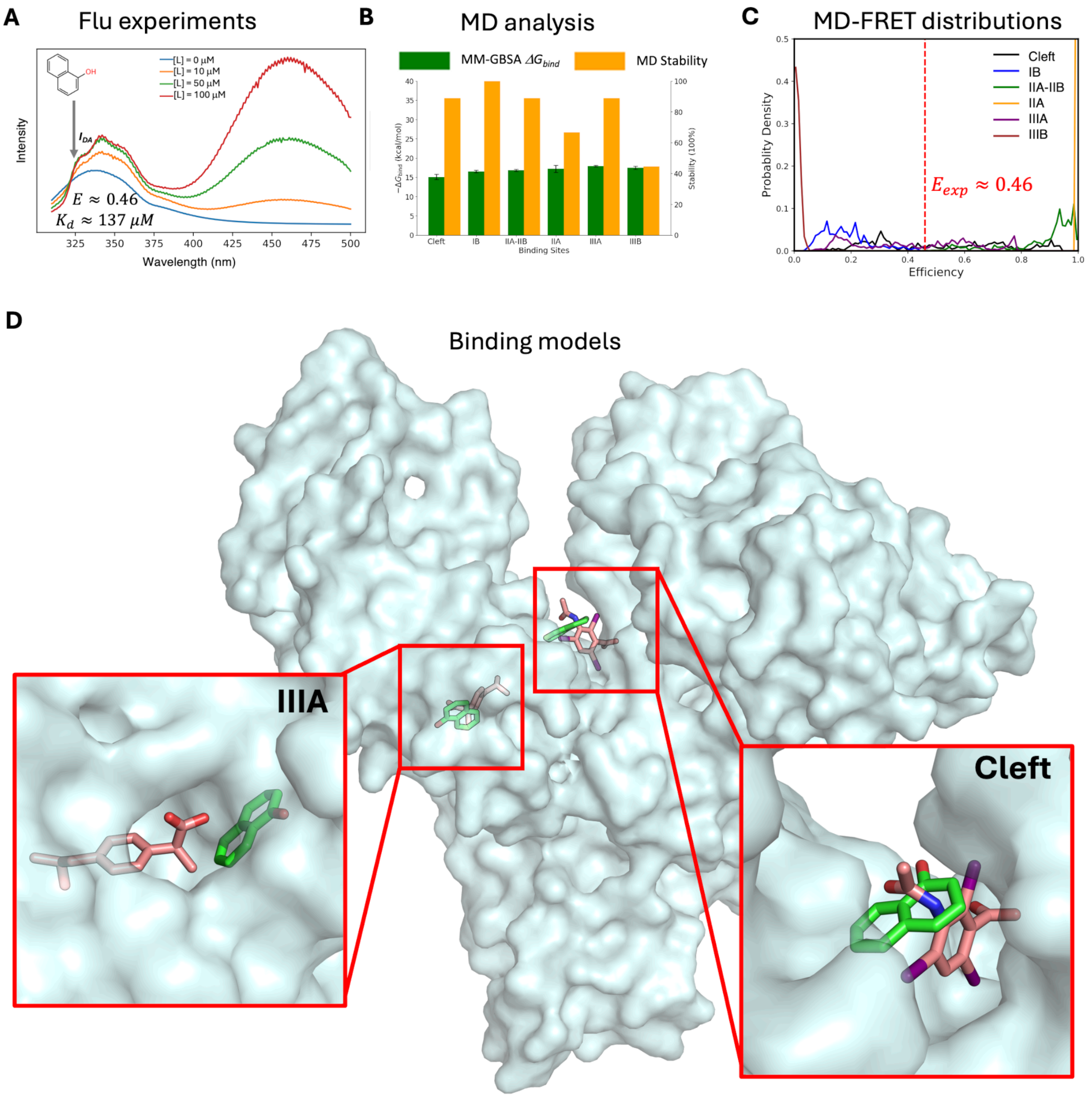

Figure S17. Binding modes determined for 1-naphthol using the TrESP-MMPol@MD protocol. **A** Fluorescence emission spectra at 295 nm excitation of the HSA–ligand complex ([P] = 5 μM HSA in PBS 1×, 25 °C) at increasing ligand concentrations ([L] = 10, 50, 100 μM), with fitted binding affinity (K_d) and experimental FRET efficiency (E_{exp}) indicated. **B** MM-GBSA binding free energies (ΔG_{bind}) and percentage of stable MD replicas from the three best docking poses in each binding site. **C** Distributions of FRET efficiencies estimated from TrESP-MMPol@MD couplings for the ligand in all six HSA sites; vertical red line = E_{exp}. **D** Binding mode in sites IIIA and Cleft (centroid of the most populated MD cluster; green) compared with ibuprofen for site IIIA in PDB ID 2BXG (pink) and iodipamide for site Cleft in PDB ID 2BXN (pink).[1]

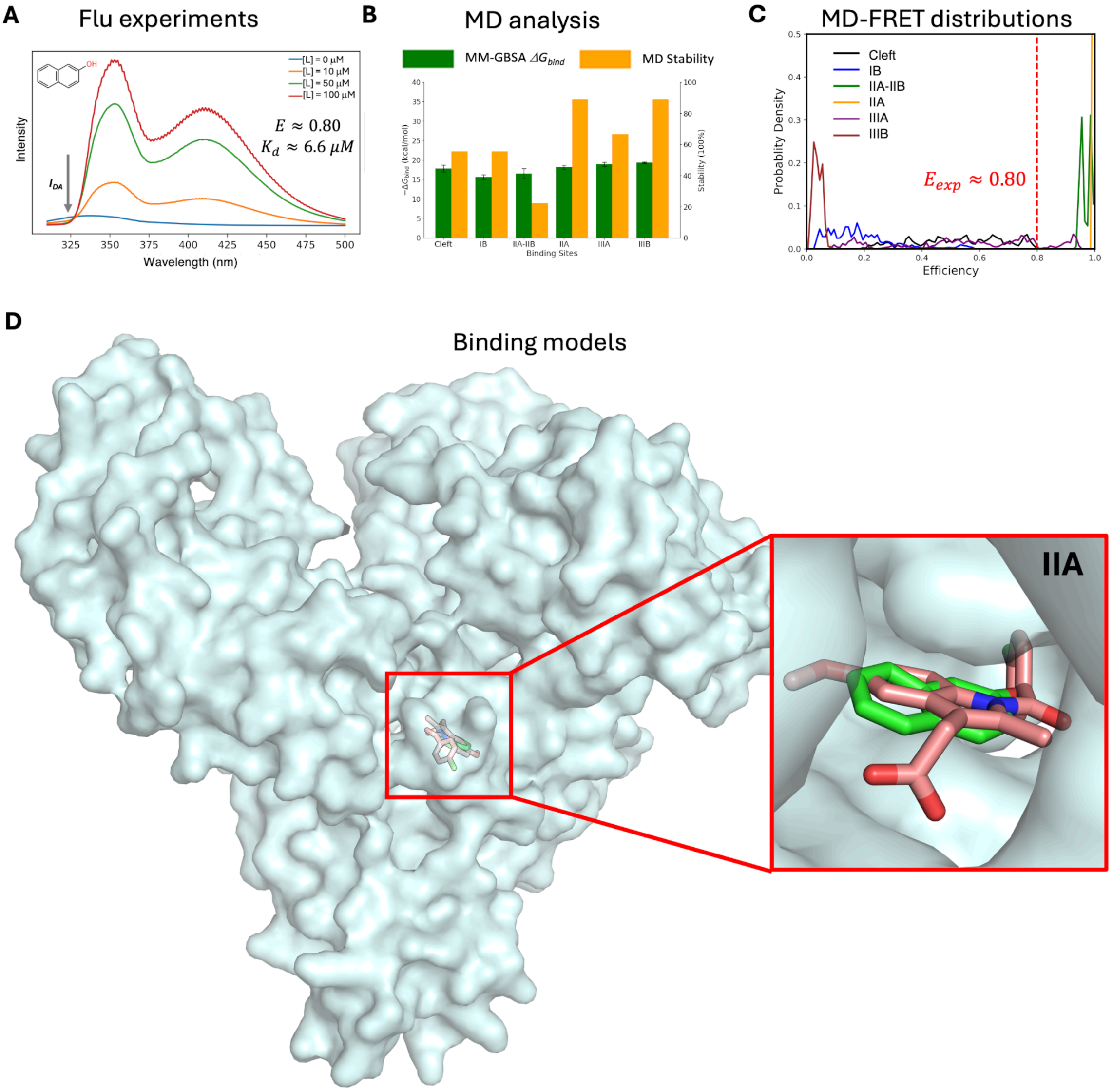

Figure S18. Binding modes determined for 2-naphthol using the TrESP-MMPol@MD protocol. **A** Fluorescence emission spectra at 295 nm excitation of the HSA–ligand complex ([P] = 5 µM HSA in PBS 1×, 25 °C) at increasing ligand concentrations ([L] = 10, 50, 100 µM), with fitted binding affinity (K_d) and experimental FRET efficiency (E_{exp}) indicated. **B** MM-GBSA binding free energies (ΔG_{bind}) and percentage of stable MD replicas from the three best docking poses in each binding site. **C** Distributions of FRET efficiencies estimated from TrESP-MMPol@MD couplings for the ligand in all six HSA sites; vertical red line = E_{exp}. **D** Binding mode in site IIA (centroid of the most populated MD cluster; green) compared with indomethacin in PDB ID 2BXM (pink).[1]

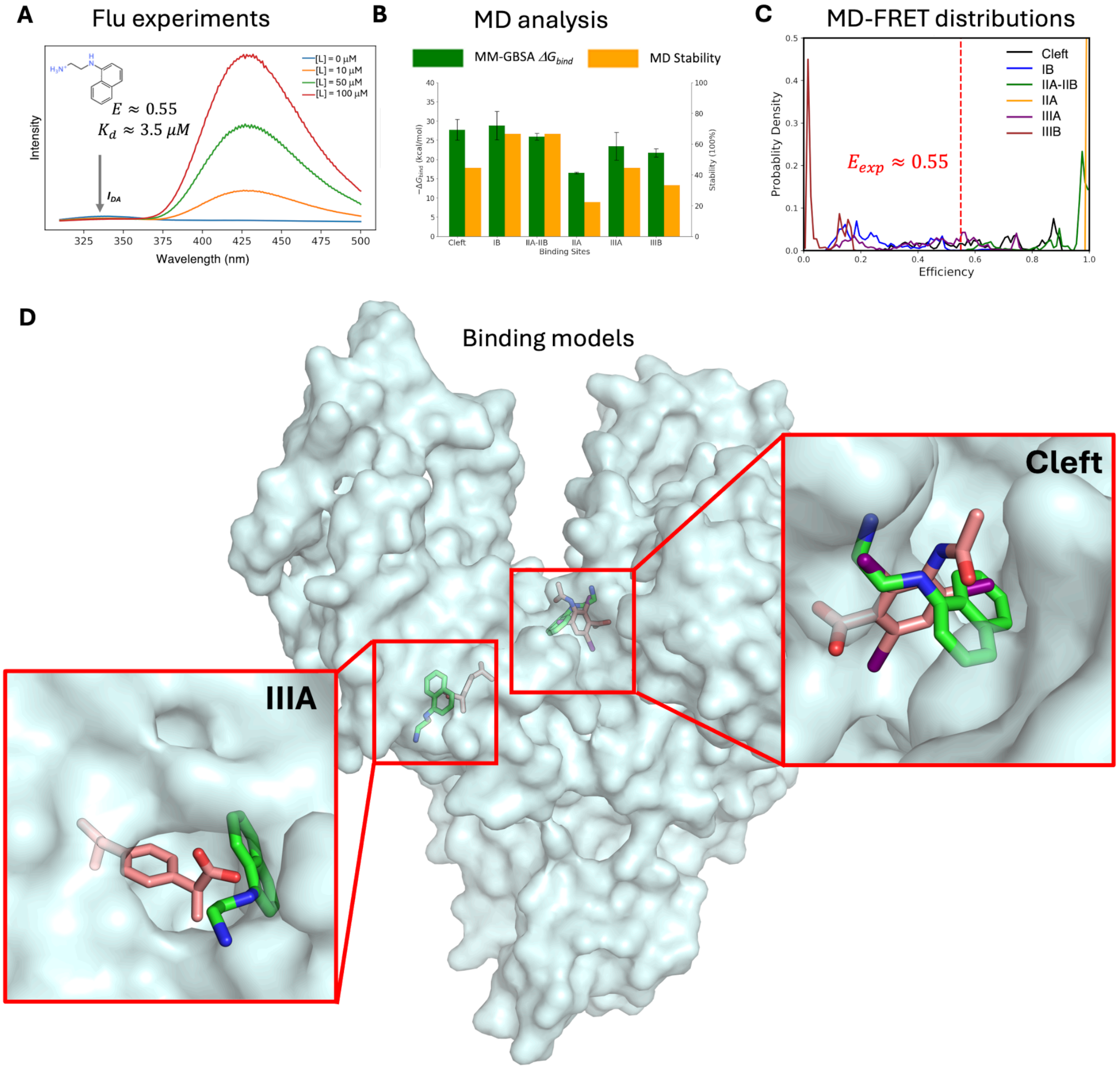

Figure S19. Binding modes determined for N-(1-naphthyl)ethylenediamine using the TrESP-MMPol@MD protocol. **A** Fluorescence emission spectra at 295 nm excitation of the HSA–ligand complex ([P] = 5 µM HSA in PBS 1×, 25 °C) at increasing ligand concentrations ([L] = 10, 50, 100 µM), with fitted binding affinity (K_d) and experimental FRET efficiency (E_{exp}) indicated. **B** MM GBSA binding free energies (ΔG_{bind}) and percentage of stable MD replicas from the three best docking poses in each binding site. **C** Distributions of FRET efficiencies estimated from TrESP MMPol@MD couplings for the ligand in all six HSA sites; vertical red line = E_{exp}. **D** Binding mode in sites IIIA and Cleft (centroid of the most populated MD cluster; green) compared with ibuprofen for site IIIA in PDB ID 2BXG (pink) and iodipamide for site Cleft in PDB ID 2BXN (pink).[1]

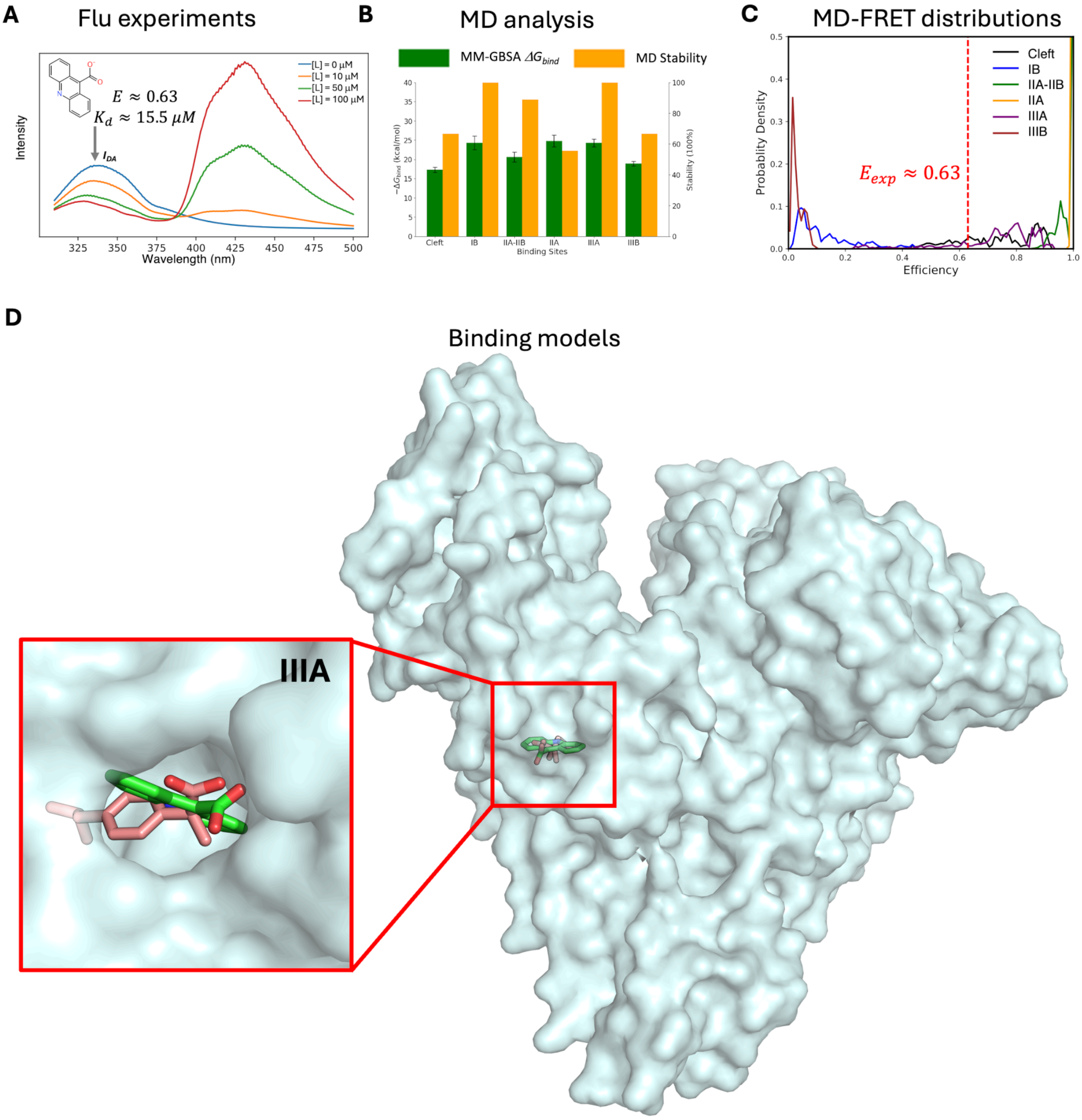

Figure S20. Binding modes determined for 9-acridinecarboxylic acid using the TrESP-MMPol@MD protocol. **A** Fluorescence emission spectra at 295 nm excitation of the HSA–ligand complex ([P] = 5 μM HSA in PBS 1×, 25 °C) at increasing ligand concentrations ([L] = 10, 50, 100 μM), with fitted binding affinity (K_d) and experimental FRET efficiency (E_{exp}) indicated. **B** MM GBSA binding free energies (ΔG_{bind}) and percentage of stable MD replicas from the three best docking poses in each binding site. **C** Distributions of FRET efficiencies estimated from TrESP MMPol@MD couplings for the ligand in all six HSA sites; vertical red line = E_{exp}. **D** Binding mode in site IIIA (centroid of the most populated MD cluster; green) compared with ibuprofen in PDB ID 2BXG (pink).[1]

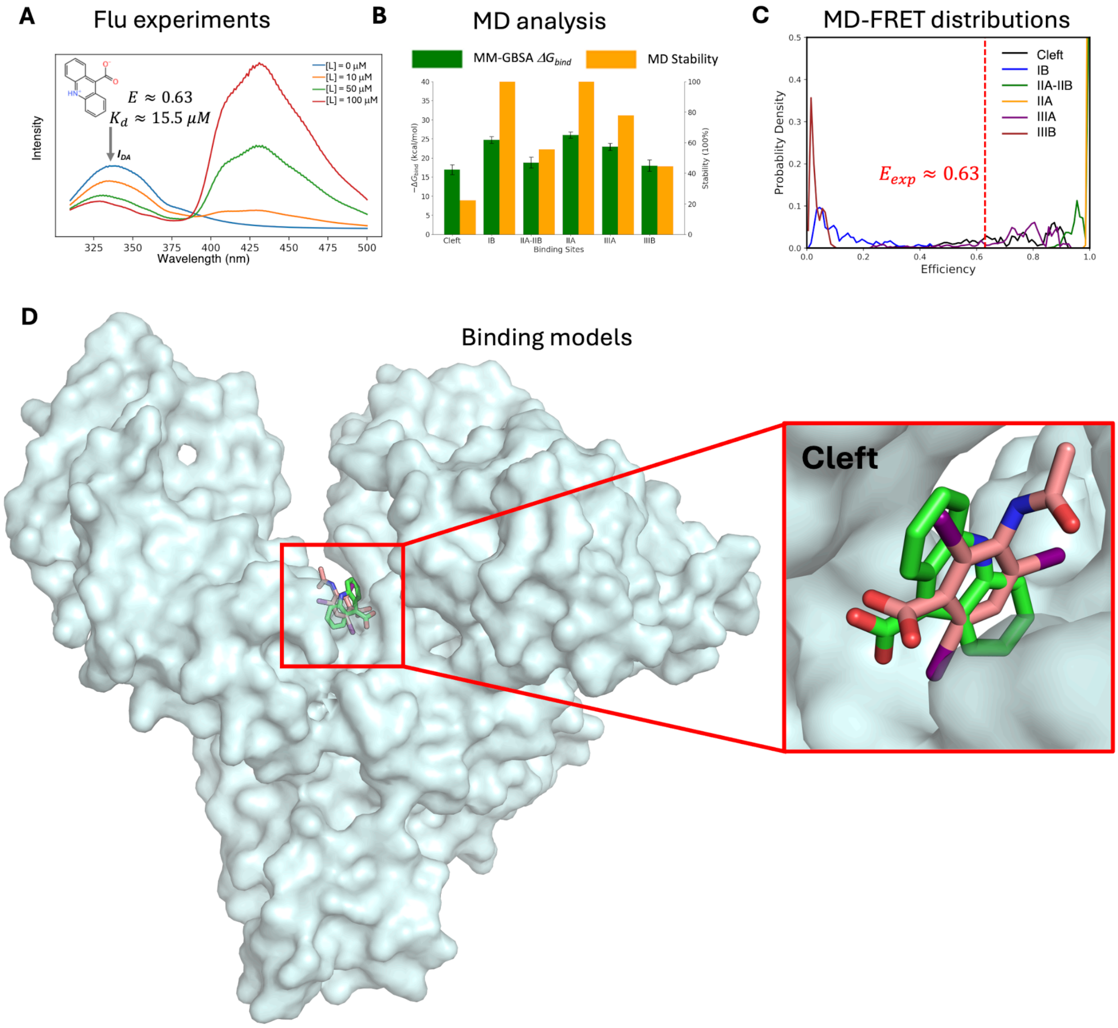

Figure S21. Binding modes determined for protonated 9-acridinecarboxylic acid using the TrESP-MMPol@MD protocol. **A** Fluorescence emission spectra at 295 nm excitation of the HSA–ligand complex ([P] = 5 µM HSA in PBS 1×, 25 °C) at increasing ligand concentrations ([L] = 10, 50, 100 µM), with fitted binding affinity (K_d) and experimental FRET efficiency (E_{exp}) indicated. **B** MM GBSA binding free energies (ΔG_{bind}) and percentage of stable MD replicas from the three best docking poses in each binding site. **C** Distributions of FRET efficiencies estimated from TrESP MMPol@MD couplings for the ligand in all six HSA sites; vertical red line = E_{exp}. **D** Binding mode in Cleft site (centroid of the most populated MD cluster; green) compared to iodipamide in PDB ID 2BXN (pink).[1]

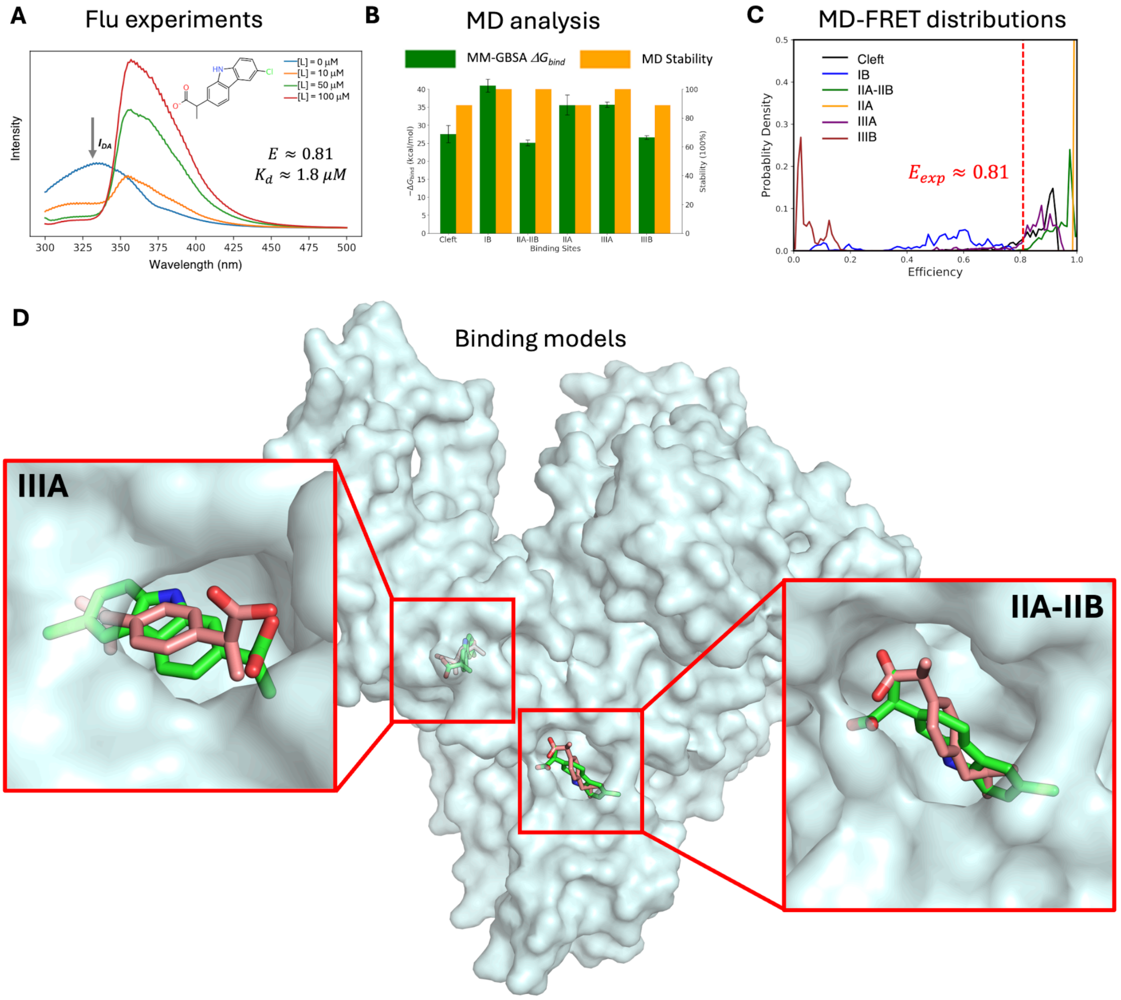

Figure S22. Binding modes determined for (*S*)-carprofen using the TrESP-MMPol@MD protocol. **A** Fluorescence emission spectrum at 280 nm excitation of the HSA–ligand complex ([P] = 5 μM HSA in PBS 1×, 25 °C) at increasing ligand concentrations ([L] = 10, 50, 100 μM), with fitted binding affinity (K_d) and experimental FRET efficiency (E_{exp}) indicated. **B** MM-GBSA binding free energies (ΔG_{bind}) and percentage of stable MD replicas from the three best docking poses in each binding site. **C** Distributions of FRET efficiencies estimated from TrESP-MMPol@MD couplings for the ligand in all six HSA sites; vertical red line = E_{exp}. **D** Binding modes in sites IIIA and IIA-IIB (centroids of the most populated MD cluster, green) compared to the binding modes of ibuprofen in PDB ID 2BXG (pink).[1]

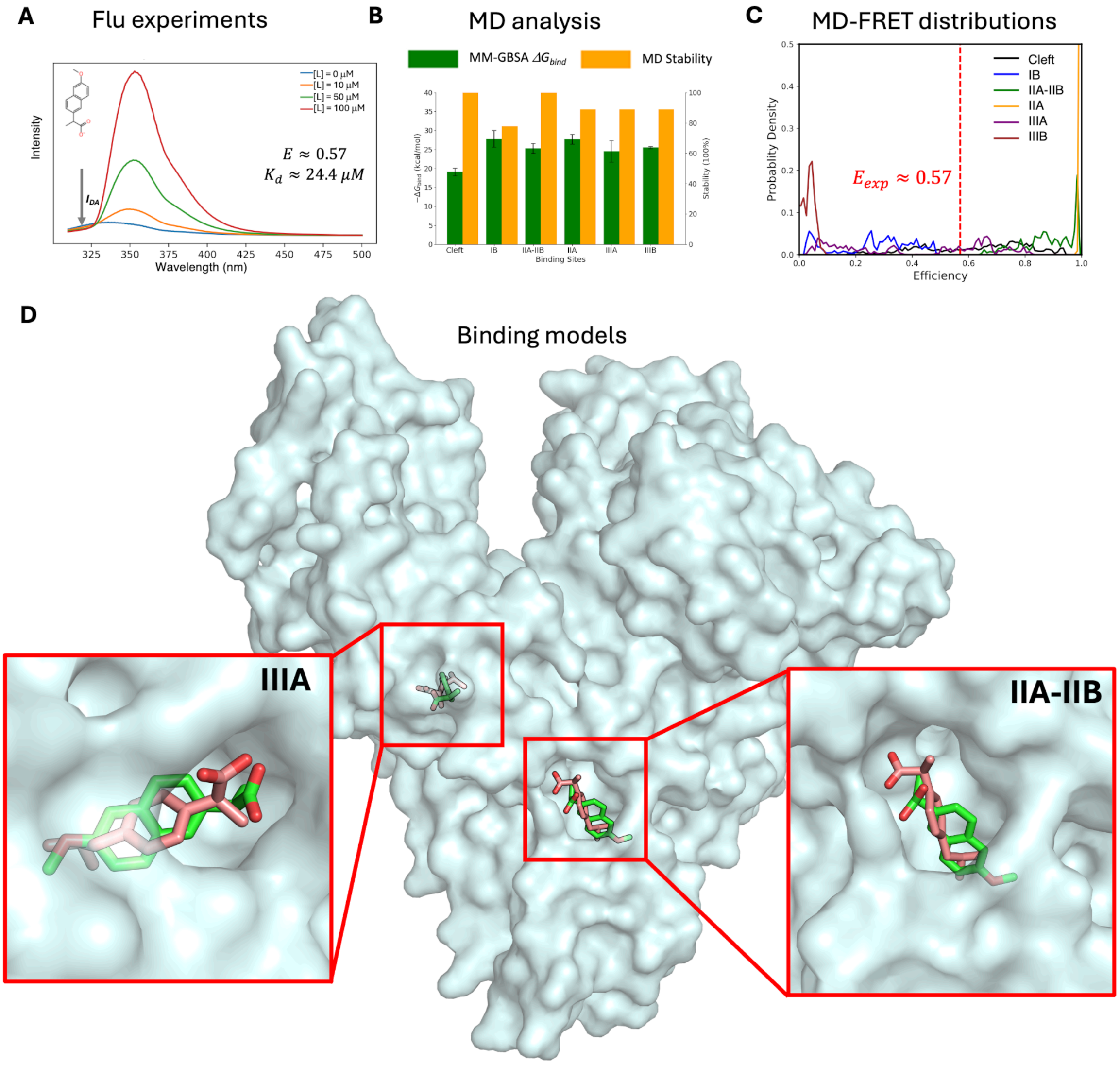

Figure S23. Binding modes determined for (*S*)-naproxen using the TrESP-MMPol@MD protocol. **A** Fluorescence emission spectrum at 295 nm excitation of the HSA–ligand complex ([P] = 5 μM HSA in PBS 1×, 25 °C) at increasing ligand concentrations ([L] = 10, 50, 100 μM), with fitted binding affinity (K_d) and experimental FRET efficiency (E_{exp}) indicated. **B** MM-GBSA binding free energies (ΔG_{bind}) and percentage of stable MD replicas from the three best docking poses in each binding site. **C** Distributions of FRET efficiencies estimated from TrESP-MMPol@MD couplings for the ligand in all six HSA sites; vertical red line = E_{exp}. **D** Binding modes in sites IIIA and IIA-IIB (centroids of the most populated MD cluster, green) compared to the binding modes of ibuprofen in PDB ID 2BXG (pink).[1]

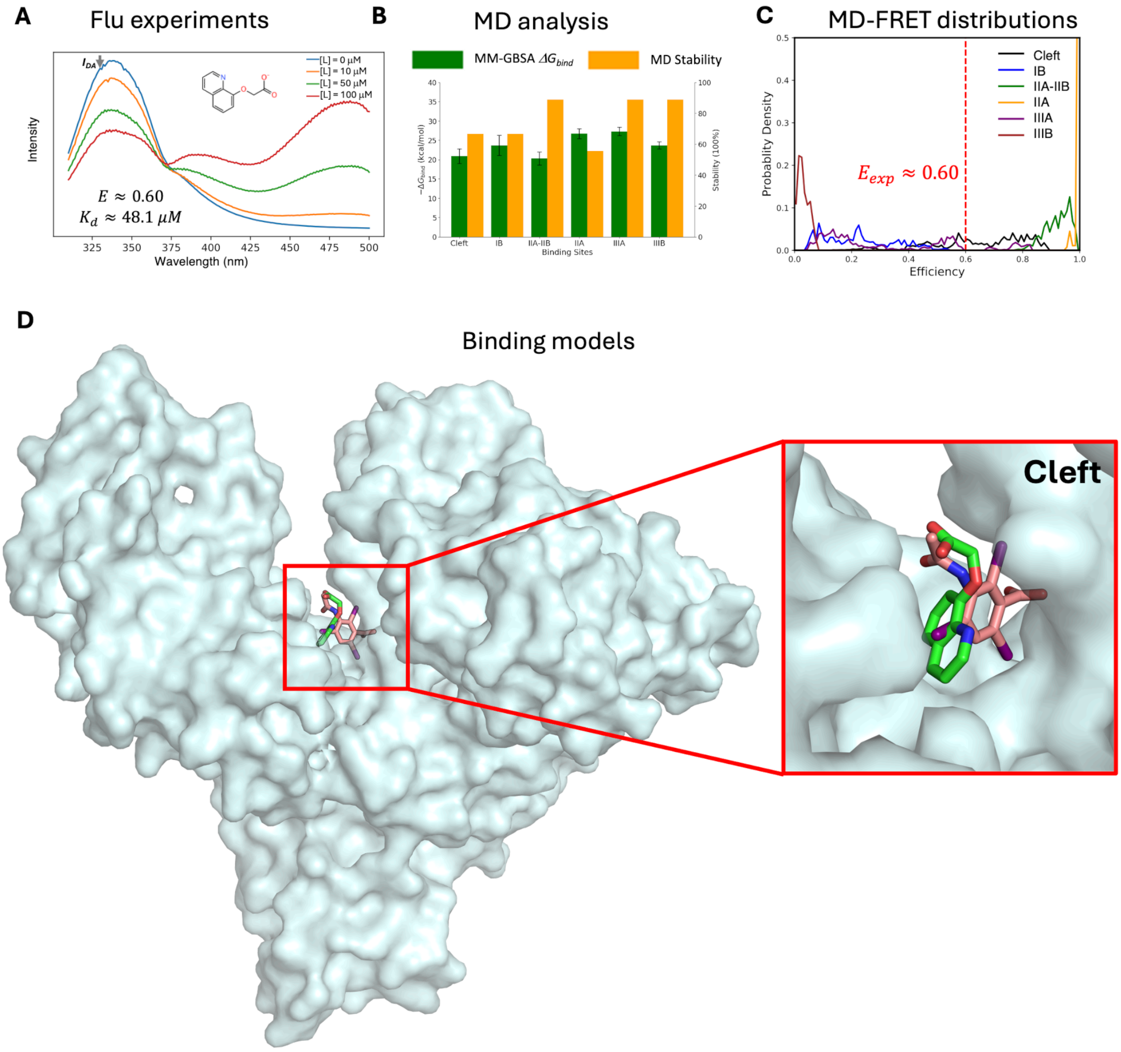

Figure S24. Binding modes determined for (quinolin-8-yloxy)-acetic acid using the TrESP-MMPol@MD protocol. **A** Fluorescence emission spectra at 295 nm excitation of the HSA–ligand complex ([P] = 5 µM HSA in PBS 1×, 25 °C) at increasing ligand concentrations ([L] = 10, 50, 100 µM), with fitted binding affinity (K_d) and experimental FRET efficiency (E_{exp}) indicated. **B** MM GBSA binding free energies (ΔG_{bind}) and percentage of stable MD replicas from the three best docking poses in each binding site. **C** Distributions of FRET efficiencies estimated from TrESP MMPol@MD couplings for the ligand in all six HSA sites; vertical red line = E_{exp}. **D** Binding mode in Cleft site (centroid of the most populated MD cluster; green) compared to iodipamide in PDB ID 2BXN (pink).[1]

1-Naphthol

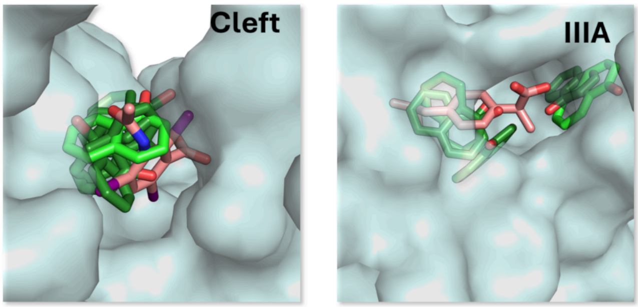

Cluster	Population (%)	
	Cleft	IIIA
1	43	41
2	24	29
3	21	21
4	11	7
5	1	2

2-Naphthol

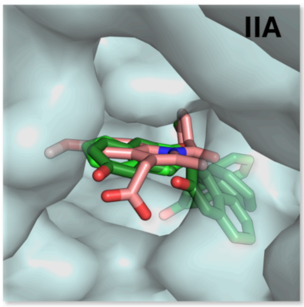

Cluster	Population IIA (%)
1	40
2	19
3	19
4	12
5	10

N-(1-Naphthyl)ethylenediamine

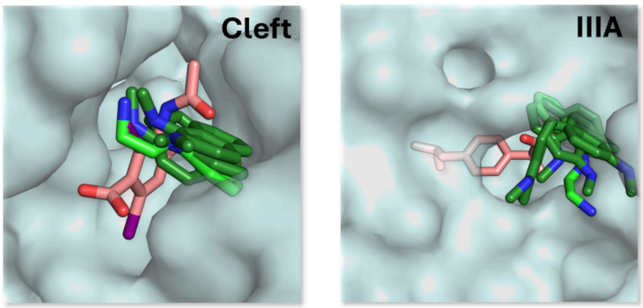

Cluster	Population (%)	
	Cleft	IIIA
1	49	34
2	15	33
3	14	25
4	14	7
5	8	1

9-acridinecarboxylic acid

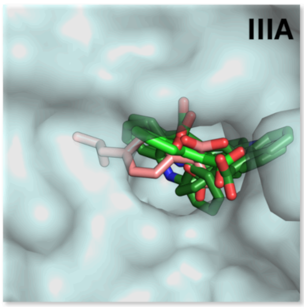

Cluster	Population IIIA (%)
1	46
2	20
3	14
4	11
5	9

Figure S25. Binding modes determined for 1-naphthol, 2-naphthol, N-(1-naphthyl)ethylenediamine and 9-acridinecarboxylic acid using the TrESP-MMPol@MD protocol (centroid of the most populated MD clusters 1 in light green, other clusters in dark green) compared with iodipamide for site Cleft in PDB ID 2BXN (pink), indomethacin for site IIA in PDB ID 2BXM (pink), and ibuprofen for site IIIA in PDB ID 2BXG (pink).[1]

Carprofen

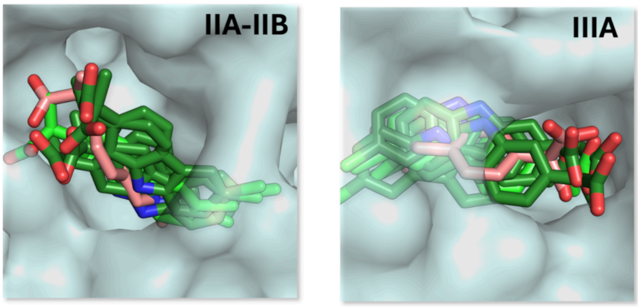

Cluster	Population (%)	
	IIA-IIB	IIIA
1	42	33
2	29	27
3	12	17
4	11	17
5	6	6

Naproxen

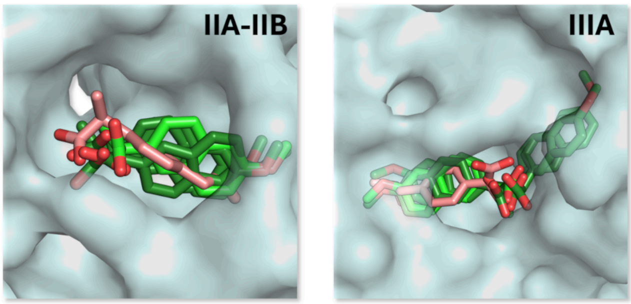

Cluster	Population (%)	
	IIA-IIB	IIIA
1	42	28
2	24	20
3	17	20
4	16	16
5	1	16

(quinolin-8-yloxy)-acetic acid

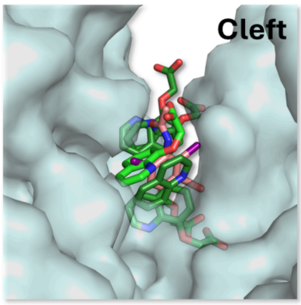

Cluster	Population Cleft (%)
1	33
2	17
3	17
4	17
5	16

Indomethacin

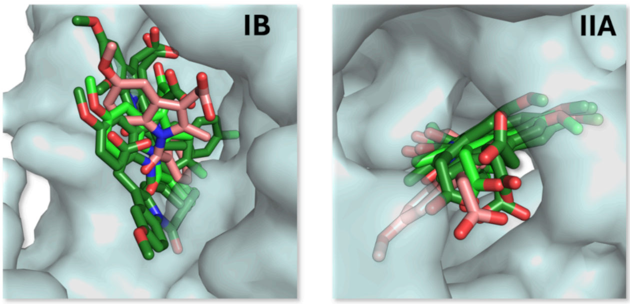

Cluster	Population (%)	
	IB	IIA
1	32	30
2	27	27
3	18	25
4	16	17
5	7	1

Figure S26. Binding modes determined for (*S*)-carprofen, (*S*)-naproxen, (quinolin-8-yloxy)-acetic acid and indomethacin using the TrESP-MMPol@MD protocol (centroid of the most populated MD clusters 1 in light green, other clusters in dark green) compared with the known binding modes of indomethacin for sites IB and IIA in PDB ID 2BXM (pink), iodipamide for site Cleft in PDB ID 2BXN (pink) and ibuprofen for sites IIA-IIB and IIIA in PDB ID 2BXG (pink).[1]

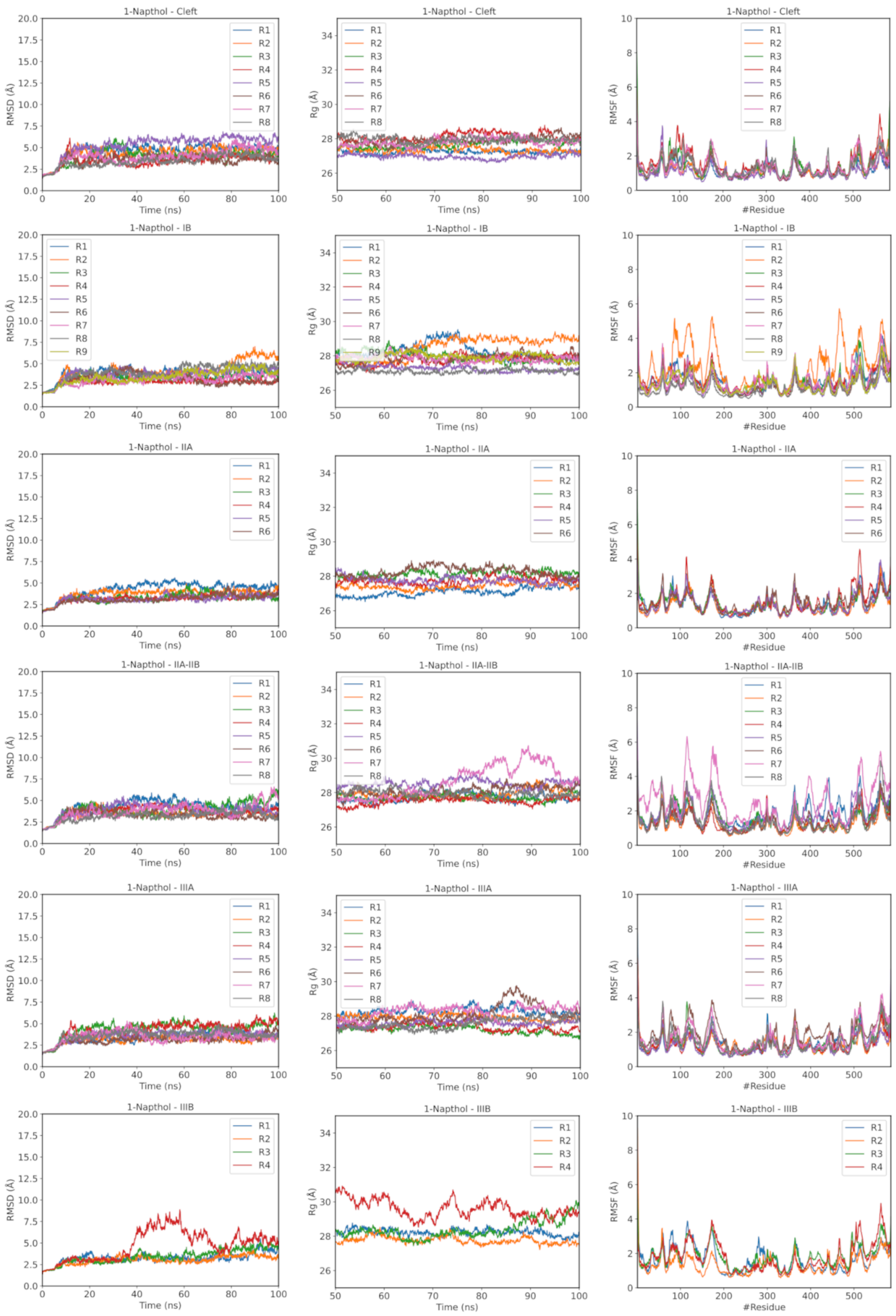

Figure S27. Protein root mean square deviation (RMSD), mass-weighted radius of gyration R_g and root mean square fluctuations (RMSF) derived from MD replicas of 1-naphthol bound to the six binding sites of HSA used to simulate FRET observables. RMSF and R_g computed for the last 50 ns of the 100 ns trajectories.

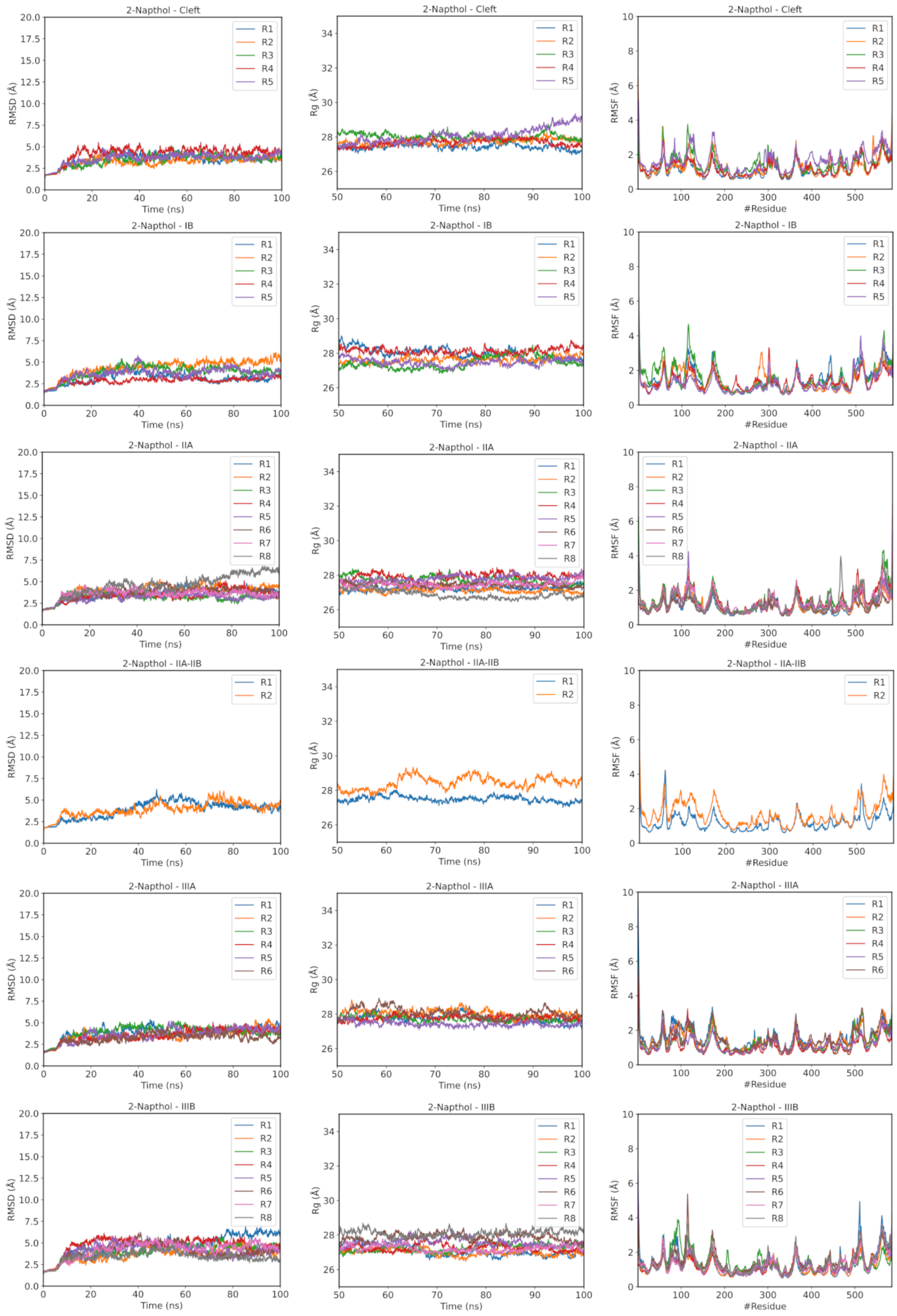

Figure S28. Protein root mean square deviation (RMSD), mass-weighted radius of gyration R_g and root mean square fluctuations (RMSF) derived from MD replicas of 2-naphthol bound to the six binding sites of HSA used to simulate FRET observables. RMSF and R_g computed for the last 50 ns of the 100 ns trajectories.

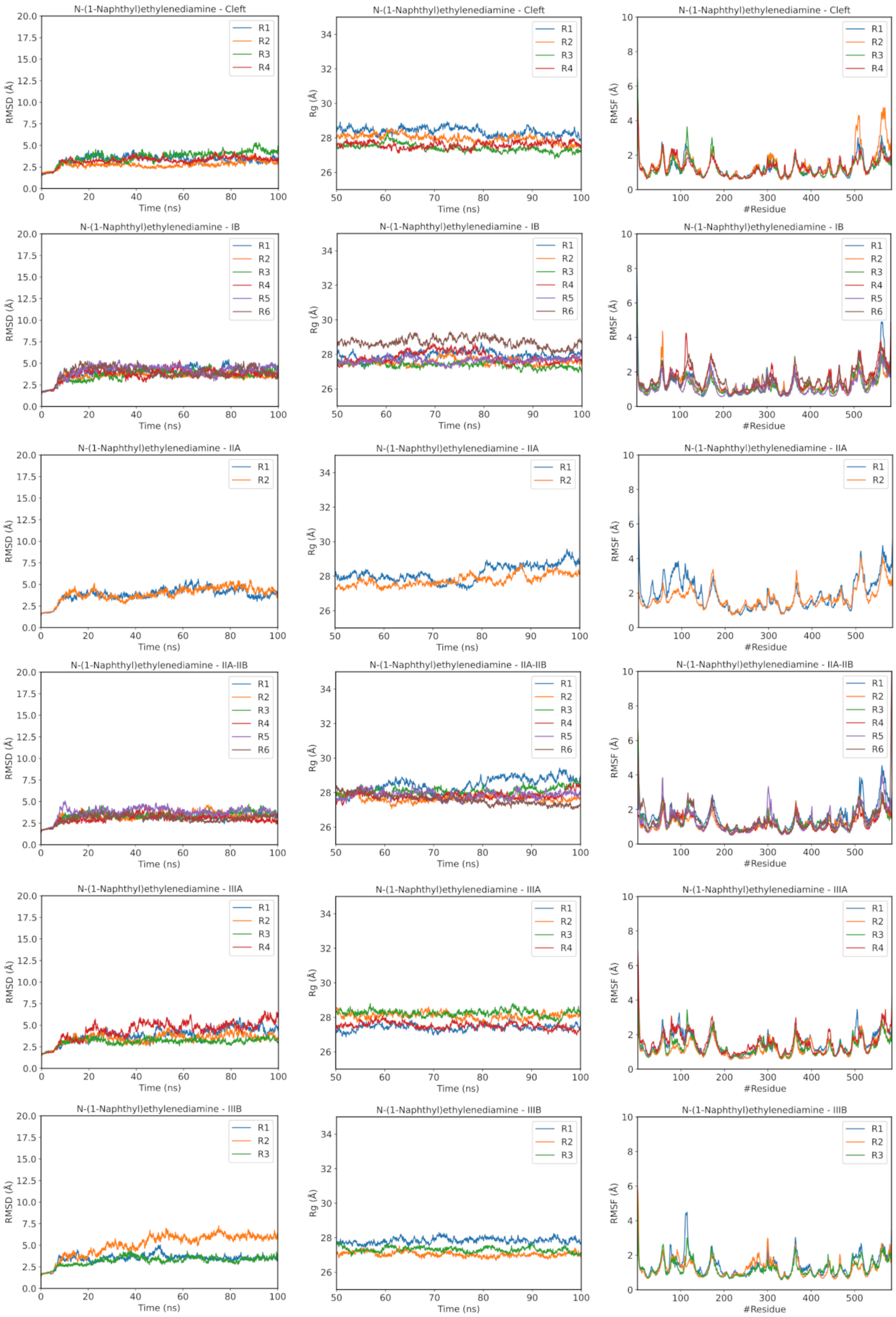

Figure S29. Protein root mean square deviation (RMSD), mass-weighted radius of gyration R_g and root mean square fluctuations (RMSF) derived from MD replicas of N-(1-naphthyl)ethylenediamine bound to the six binding sites of HSA used to simulate FRET observables. RMSF and R_g computed for the last 50 ns of the 100 ns trajectories.

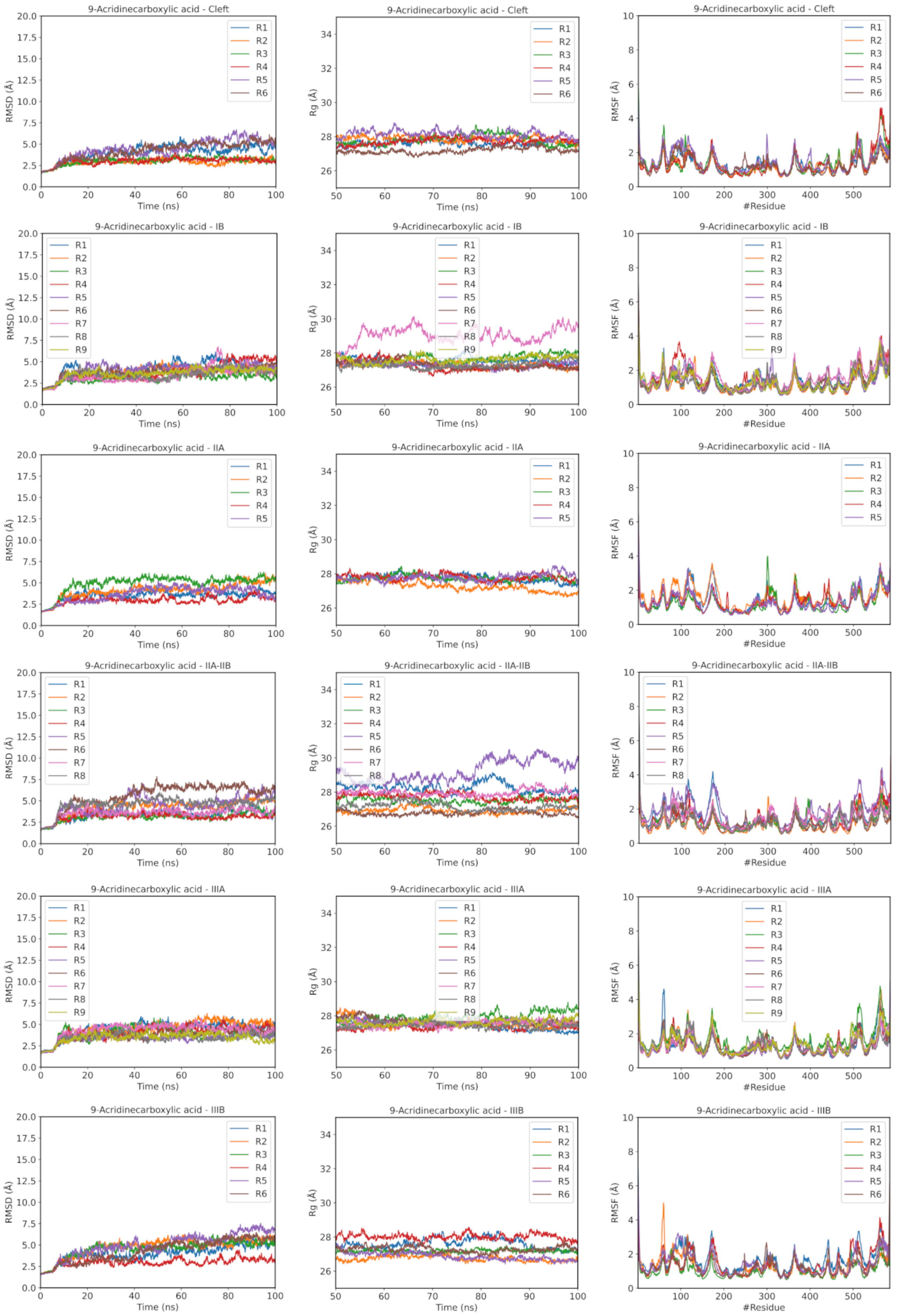

Figure S30. Protein root mean square deviation (RMSD), mass-weighted radius of gyration R_g and root mean square fluctuations (RMSF) derived from MD replicas of 9-acridinecarboxylic acid bound to the six binding sites of HSA used to simulate FRET observables. RMSF and R_g computed for the last 50 ns of the 100 ns trajectories.

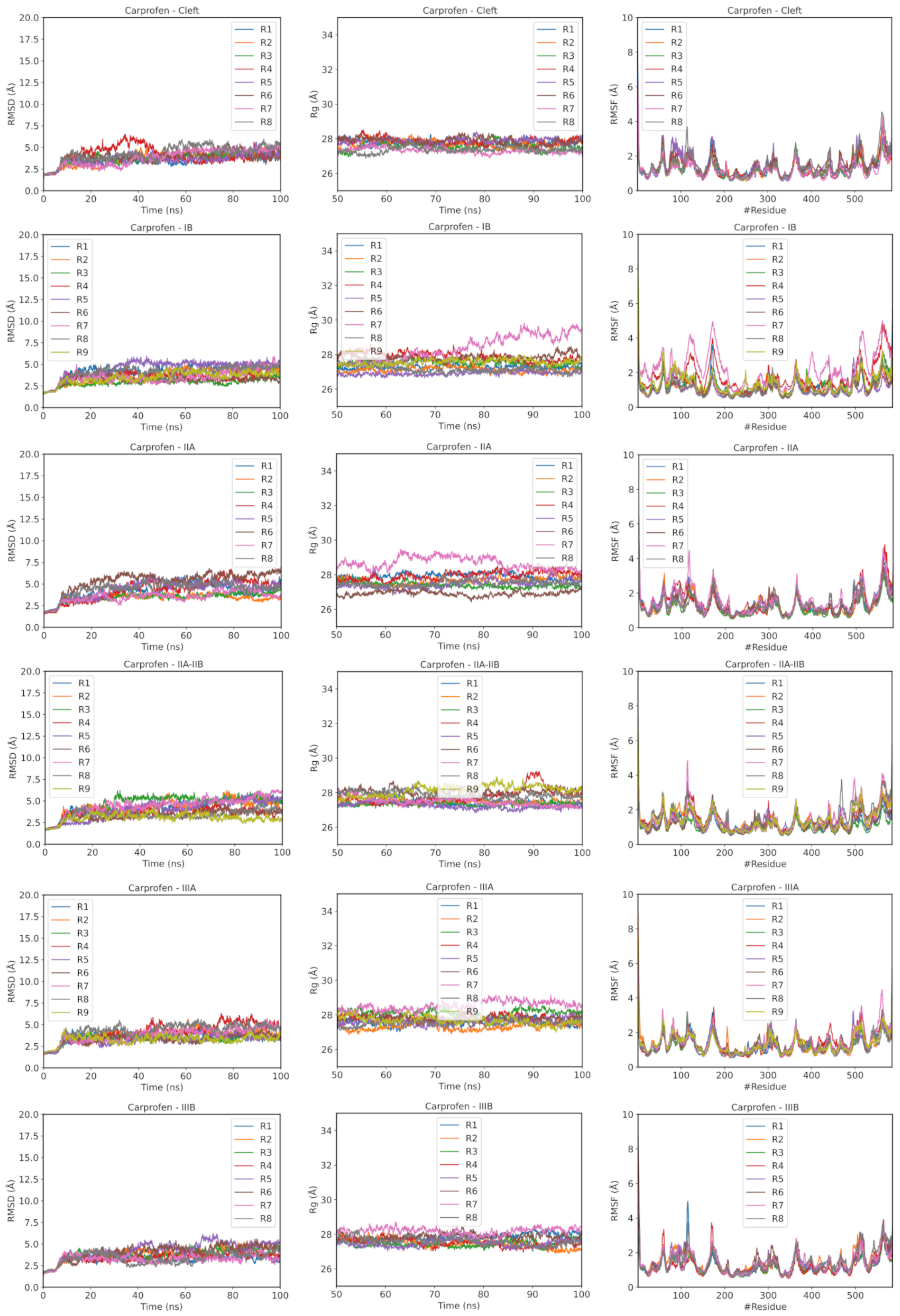

Figure S31. Protein root mean square deviation (RMSD), mass-weighted radius of gyration R_g and root mean square fluctuations (RMSF) derived from MD replicas of (*S*)-carprofen bound to the six binding sites of HSA used to simulate FRET observables. RMSF and R_g computed for the last 50 ns of the 100 ns trajectories.

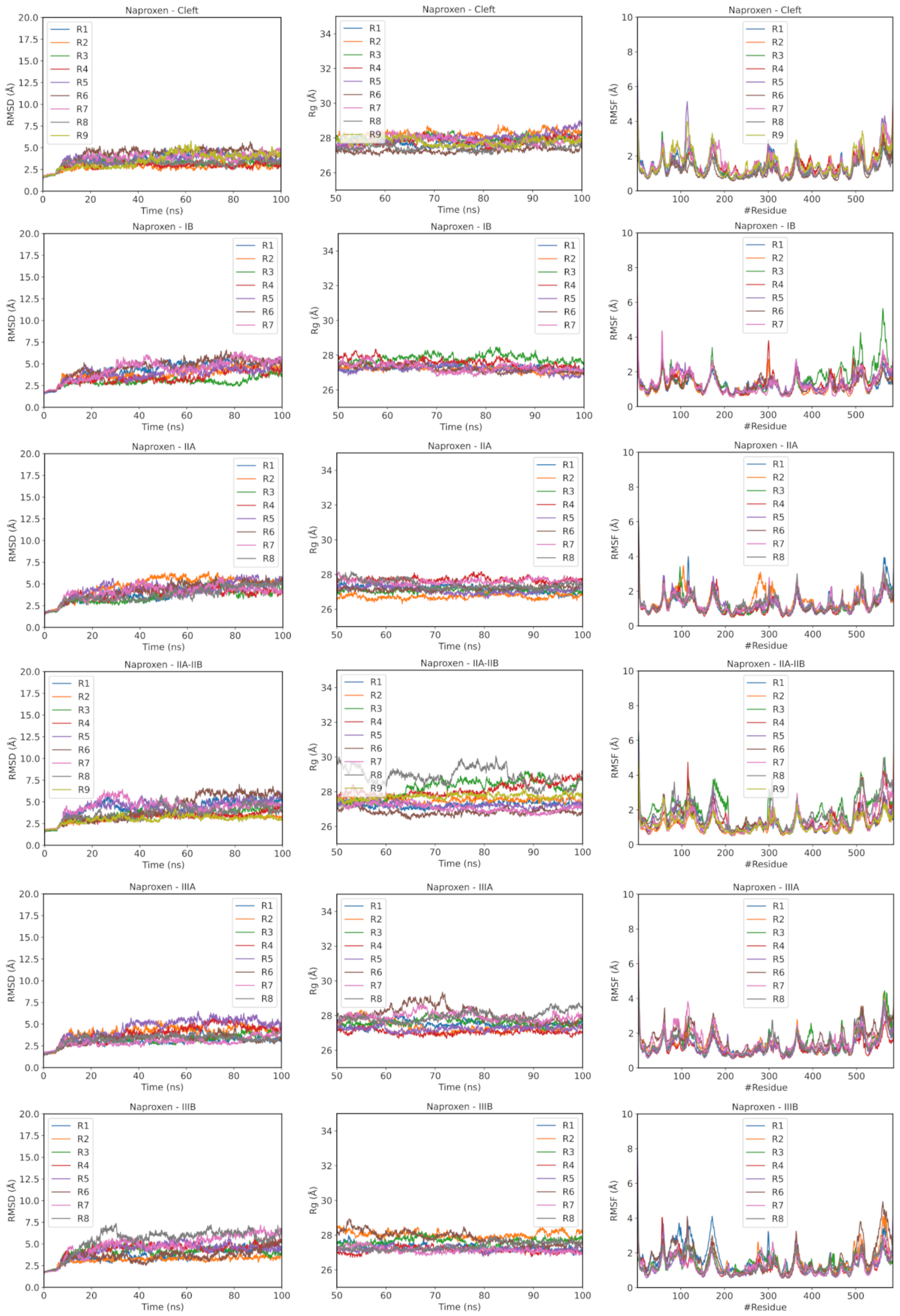

Figure S32. Protein root mean square deviation (RMSD), mass-weighted radius of gyration R_g and root mean square fluctuations (RMSF) derived from MD replicas of (*S*)-naproxen bound to the six binding sites of HSA used to simulate FRET observables. RMSF and R_g computed for the last 50 ns of the 100 ns trajectories.

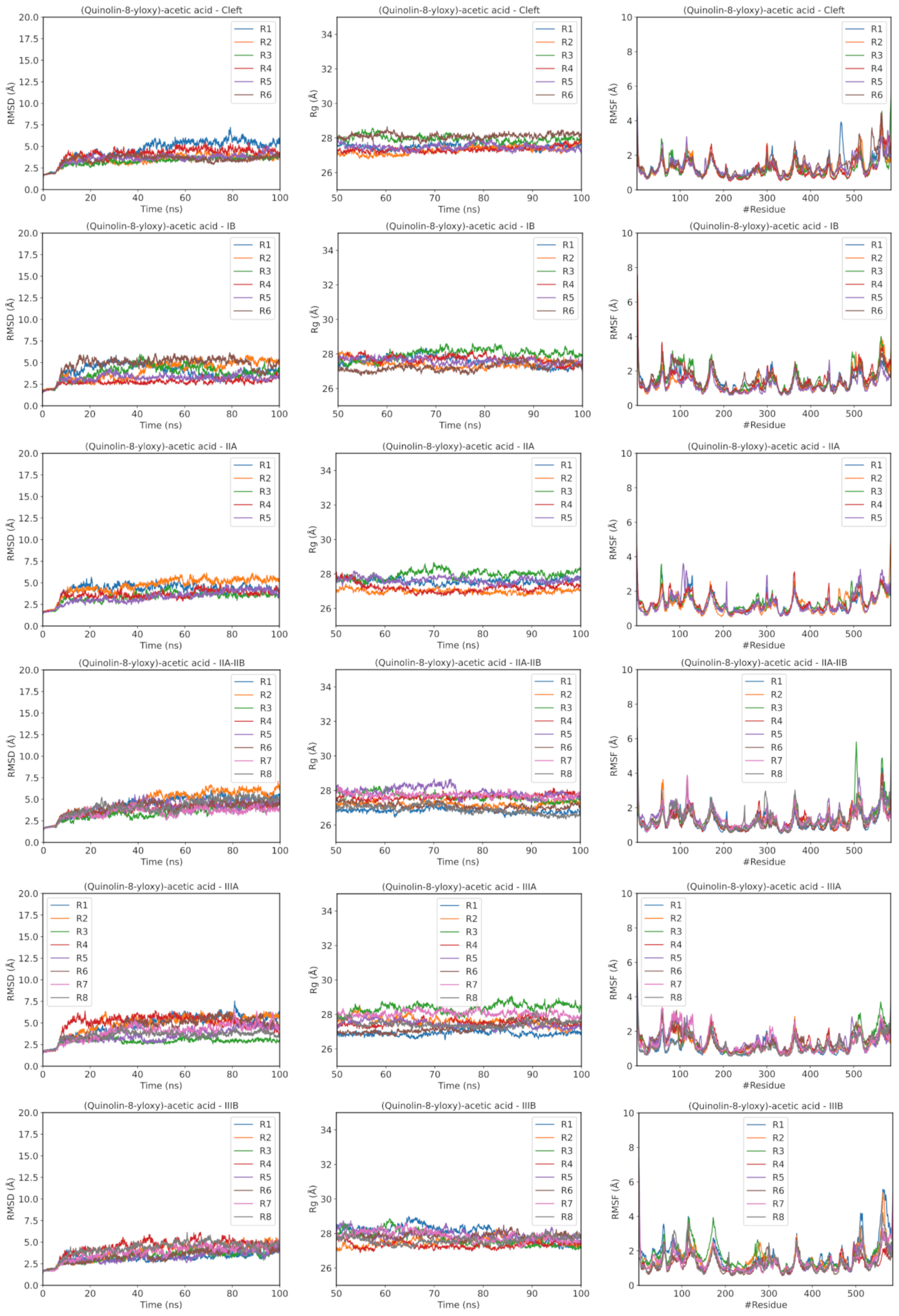

Figure S33. Protein root mean square deviation (RMSD), mass-weighted radius of gyration R_g and root mean square fluctuations (RMSF) derived from MD replicas of (quinolin-8-yloxy)-acetic bound to the six binding sites of HSA used to simulate FRET observables. RMSF and R_g computed for the last 50 ns of the 100 ns trajectories.

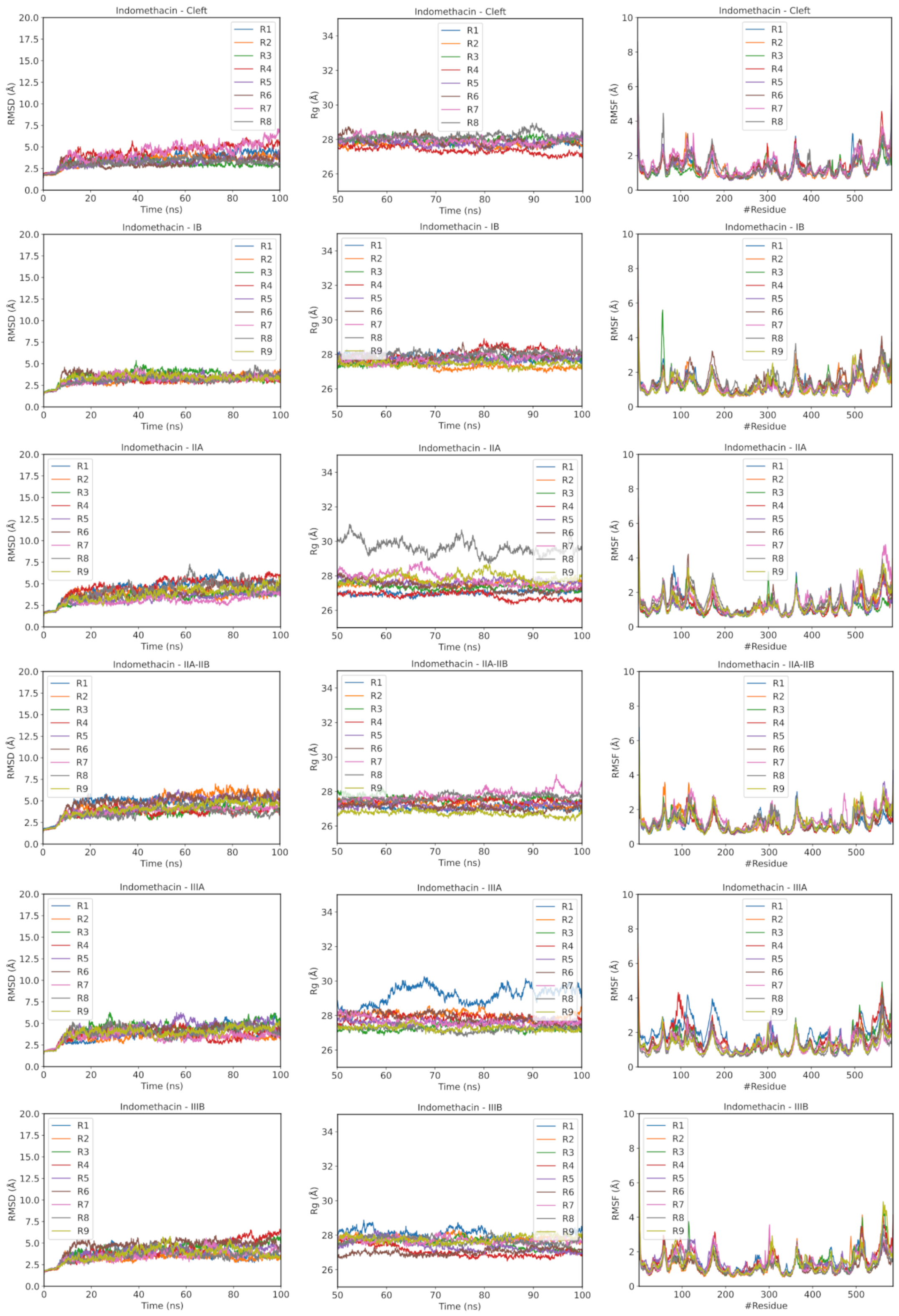

Figure S34. Protein root mean square deviation (RMSD), mass-weighted radius of gyration R_g and root mean square fluctuations (RMSF) derived from MD replicas of indomethacin bound to the six binding sites of HSA used to simulate FRET observables. RMSF and R_g computed for the last 50 ns of the 100 ns trajectories.

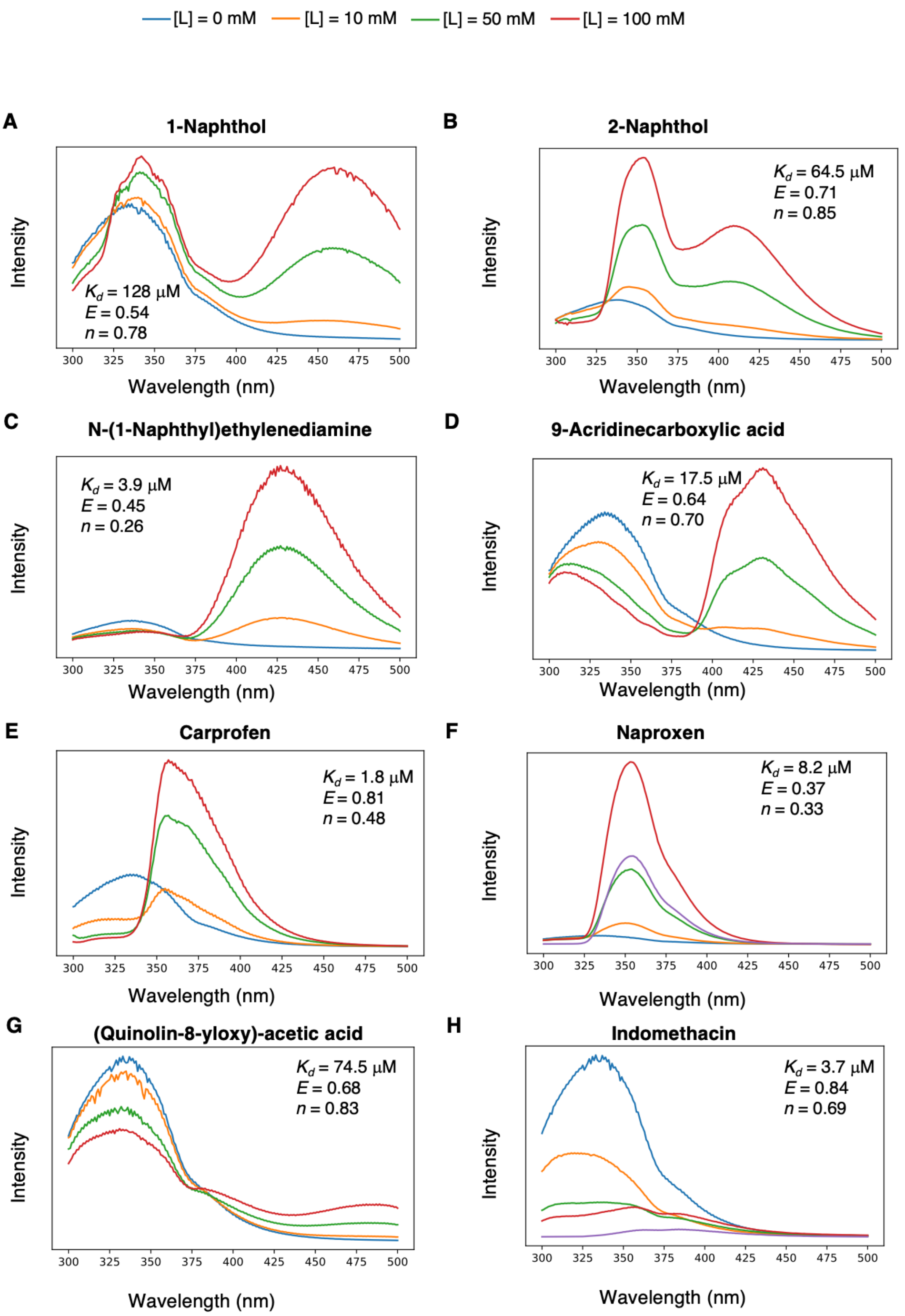

Figure S35. Fluorescence emission spectra of HSA–ligand complexes recorded at [P] = 5 μM human serum albumin (HSA) in PBS 1× buffer, 25 °C, with increasing ligand concentrations ([L] = 0, 10, 50, and 100 μM). For each ligand, the fitted dissociation constant (K_d), experimental FRET efficiency (E_{exp}), and Hill coefficient (n) are given in the inset. Ligands: 1-naphthol, 2-naphthol, N-(1-naphthyl)ethylenediamine, 9-acridinecarboxylic acid, (*S*)-carprofen, (*S*)-naproxen, (quinolin-8-yloxy)-acetic acid and indomethacin. Emission range: 300–500 nm upon excitation at 280 nm.

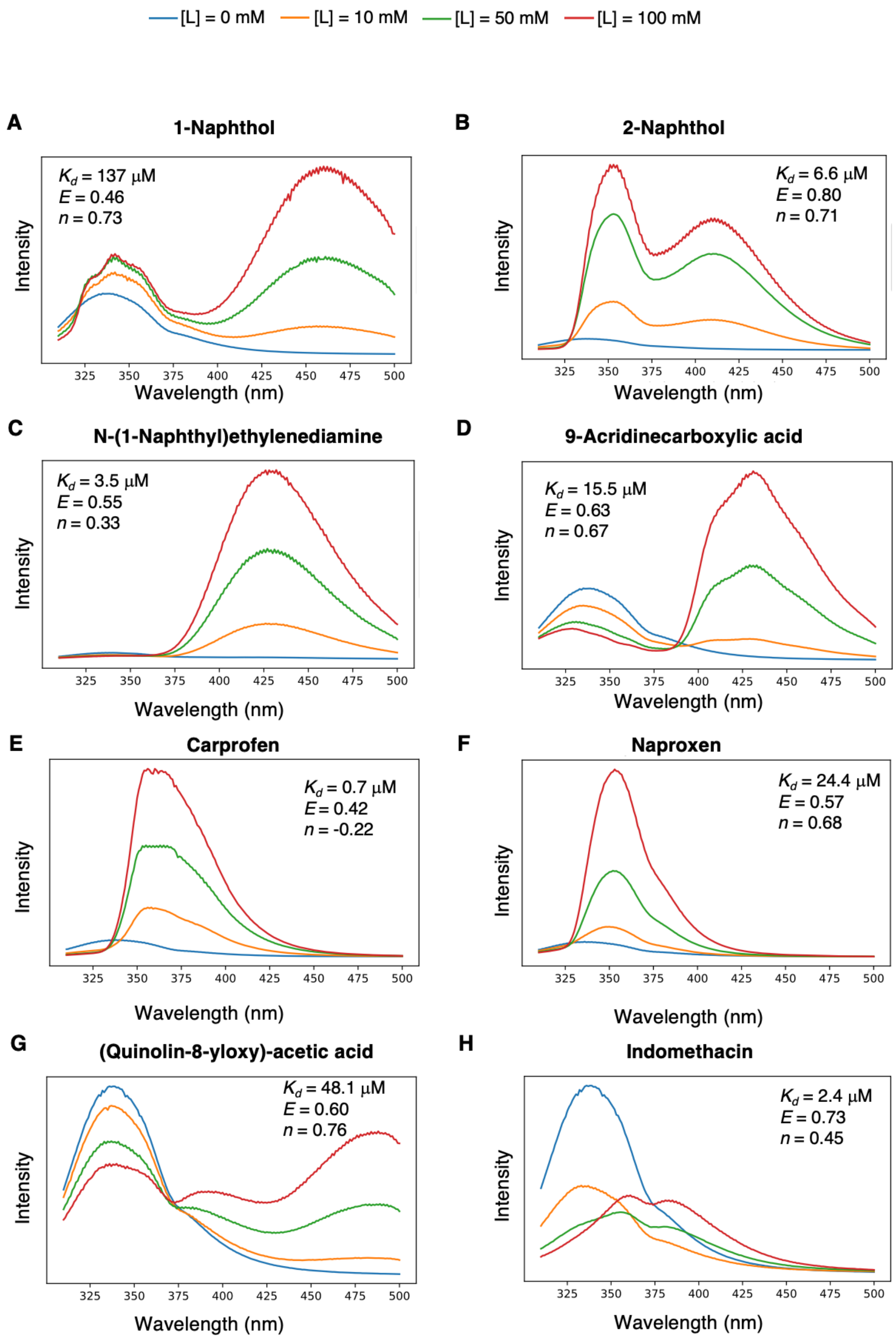

Figure S36. Fluorescence emission spectra of HSA–ligand complexes recorded at [P] = 5 μM human serum albumin (HSA) in PBS 1× buffer, 25 °C, with increasing ligand concentrations ([L] = 0, 10, 50, and 100 μM). For each ligand, the fitted dissociation constant (K_d), experimental FRET efficiency (E_{exp}), and Hill coefficient (n) are given in the inset. Ligands: 1-naphthol, 2-naphthol, N-(1-naphthyl)ethylenediamine, 9-acridinecarboxylic acid, (S)-carprofen, (S)-naproxen, (quinolin-8-yloxy)-acetic acid and indomethacin. Emission range: 310–500 nm upon excitation at 295 nm.

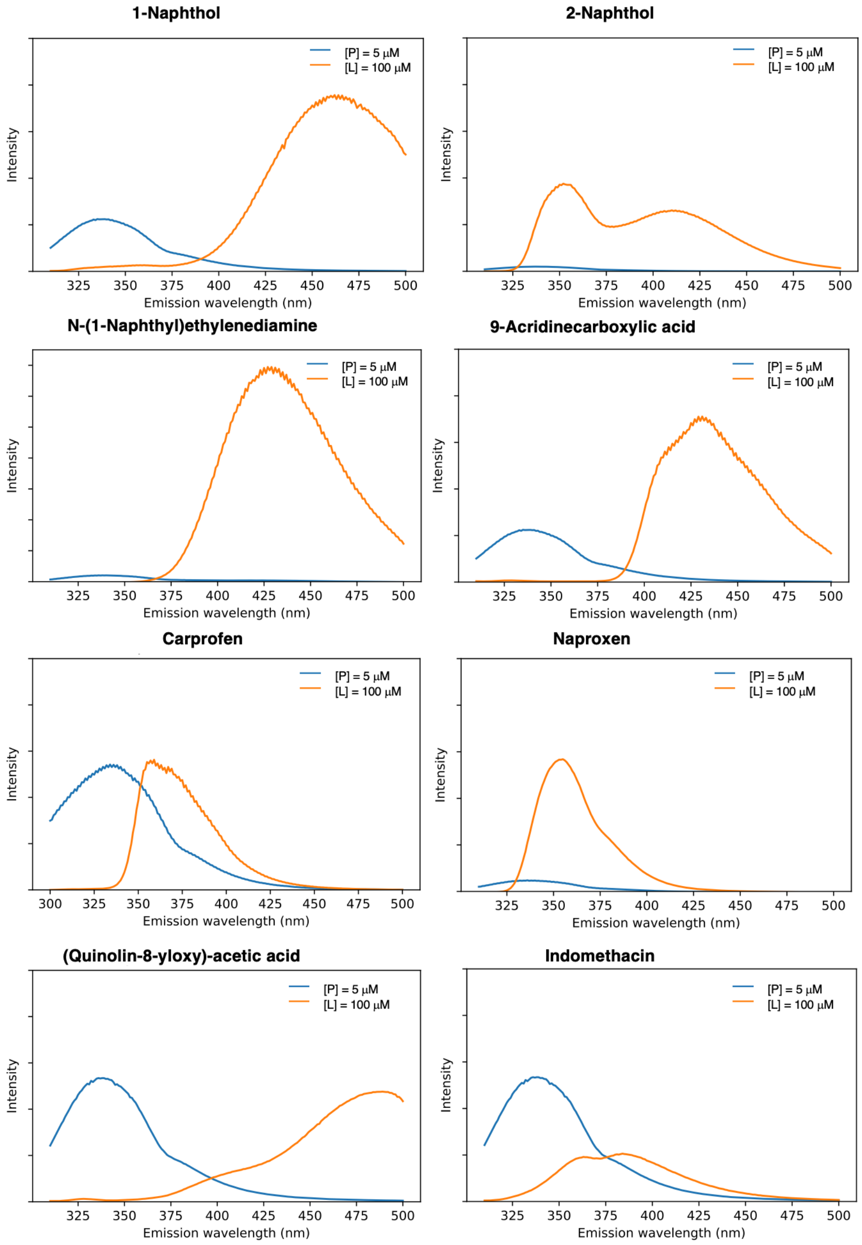

Figure S37. Fluorescence emission spectra of individual ligands (100 µM) and HSA (5 µM) in PBS 1× buffer, 25 °C. Each panel shows: ligand alone and HSA alone. Ligands: 1-naphthol, 2-naphthol, N-(1-naphthyl)ethylenediamine, 9-acridinecarboxylic acid, (*S*)-carprofen, (*S*)-naproxen, (quinolin-8-yloxy)-acetic acid and indomethacin. Excitation wavelength = 295 nm (emission collected over 310–500 nm) except for (*S*)-carprofen with excitation wavelength = 280 nm (emission collected over 300–500 nm).

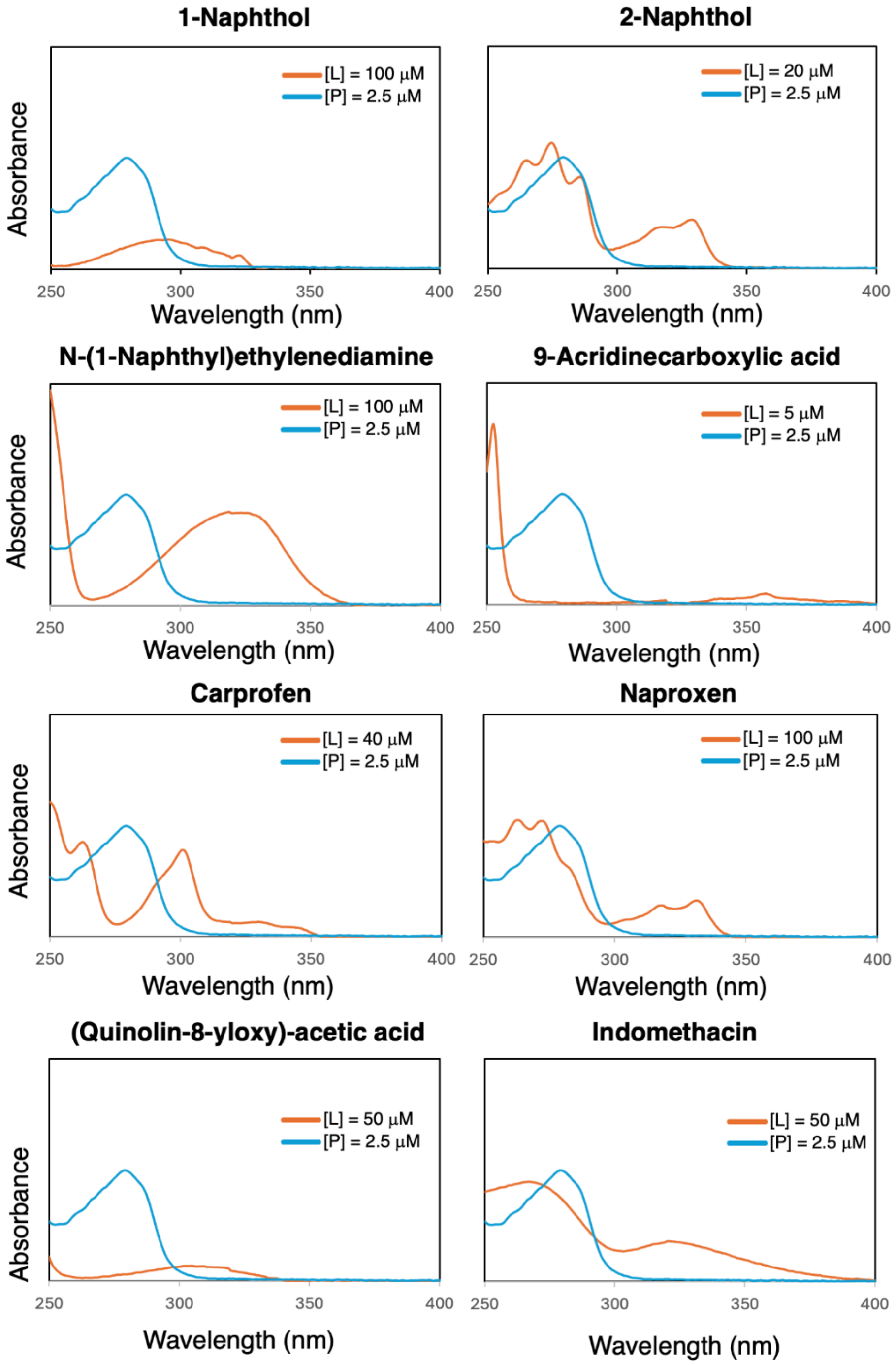

Figure S38. UV–visible absorption spectra of HSA (2.5 µM) and the ligands 1-naphthol, 2-naphthol, N-(1-naphthyl)ethylenediamine, 9-acridinecarboxylic acid, (*S*)-carprofen, (*S*)-naproxen, (quinolin-8-yloxy)-acetic acid and indomethacin in PBS 1× buffer, 25 °C. Ligand concentrations matched those in fluorescence experiments ([L] specified in each panel; typically 5–100 µM). Spectra recorded in the 250–400 nm range. These absorption profiles were used to compute donor–acceptor spectral overlap integrals (J) for the FRET analysis (see Table S2).

Table S1. Electronic transition energies and electric transition dipole moments computed for the relevant π→π* excited states of each ligand and for the L_a state of Trp from TD-B3LYP/6-31G(d) and TD-CAM-B3LYP/6-31G(d) calculations.

Molecule	State	Method	ΔE (ev)	μ^T (a.u.)
Tryptophan	S1	B3LYP	4.75	0.73
Tryptophan	S1	CAM-B3LYP	5.07	0.66
1-Naphthol	S1	B3LYP	4.26	0.83
2-Naphthol	S1	B3LYP	4.14	0.71
N-(1-Naphthyl)ethylenediamine	S3	B3LYP	4.21	10.24
9-Acridinecarboxylic acid	S3	CAM-B3LYP	3.62	10.87
9-Acridinecarboxylic acid (protonated)	S3	CAM-B3LYP	2.77	11.90
(*S*)-Carprofen	S1	CAM-B3LYP	3.71	19.06
(*S*)-Naproxen	S1	CAM-B3LYP	3.69	0.81
(Quinolin-8-yloxy)-acetic acid	S4	CAM-B3LYP	4.08	0.96
Indomethacin	S1	CAM-B3LYP	3.27	10.66

Table S2. Spectral overlaps J and Förster critical radius R_o calculated for the different ligand – Trp pairs from Eq. 1 and 2 (main text) adopting values $n^2 = 2$, $\kappa^2 = 2/3$ and a lifetime for HSA $\tau_D = 5.78$ ns.[2]

Ligand	Method	J (ev)	R_o (Å)
1-Naphthol	B3LYP	0.3989	16.7
2-Naphthol	B3LYP	0.9518	18.4
N-(1-Naphthyl)ethylenediamine	B3LYP	0.8249	20.2
9-Acridinecarboxylic acid	CAM-B3LYP	0.8762	20.2
9-Acridinecarboxylic acid (protonated)	CAM-B3LYP	0.8762	20.8
(*S*)-Carprofen	CAM-B3LYP	0.6216	23.0
(*S*)-Naproxen	CAM-B3LYP	0.9773	18.7
(Quinolin-8-yloxy)-acetic acid	CAM-B3LYP	0.6038	18.2
Indomethacin	CAM-B3LYP	0.9569	20.0

Table S3. FRET efficiencies calculated from TrESP-MMPol@MD coupling trajectories for the ligands in each binding site of HSA and efficiencies measured from fluorescence spectra (λ_{ex} = 295 nm, IFE-corrected – except for Carprofen, λ_{ex} = 280 nm, IFE-corrected). Average ligand-Trp distances sampled along MD trajectories are compared to distances estimated from the theoretical and experimental efficiencies assuming R^{-6} Förster expression. Deviations of $R(E_{\text{theo}})$ from R_{MD} arise from the limits of Förster point dipole approximation and the assumption of an isotropic distribution of donor-acceptor orientations. In bold we indicate the relevant binding modes discussed in the main text.

1-Naphthol					
	TrESP-MMPol@MD	E_{theo}	$\lvert E_{\text{exp}} - E_{\text{theo}}\rvert$	$R(E_{\text{theo}})$ (Å)	R_{MD} (Å)
	**Cleft**	**0.51 ± 0.08**	**0.05**	**16.7**	**16.8 ± 0.5**
	IB	0.18 ± 0.02	0.28	21.9	22.0 ± 0.5
	IIA-IIB	0.88 ± 0.04	0.42	11.3	11.4 ± 0.3
	IIA	1.00 ± 0.00	0.54	4.8	4.6 ± 0.1
	**IIIA**	**0.42 ± 0.06**	**0.04**	**18.1**	**18.5 ± 0.7**
	IIIB	0.01 ± 0.00	0.45	35.4	40.3 ± 0.9
	Experiment	E_{exp}		$R(E_{\text{exp}})$ (Å)	
		**0.46± 0.05**		**17.2**	
2-Naphthol					
	TrESP-MMPol@MD	E_{theo}	$\lvert E_{\text{exp}} - E_{\text{theo}}\rvert$	$R(E_{\text{theo}})$ (Å)	R_{MD} (Å)
	Cleft	0.53 ± 0.04	0.27	18.1	17.4 ± 0.5
	IB	0.19 ± 0.04	0.61	24.3	23.5 ± 1.2
	IIA-IIB	0.97 ± 0.01	0.17	10.1	11.6 ± 0.3
	**IIA**	**1.00 ± 0.00**	**0.20**	**5.2**	**5.4 ± 0.2**
	IIIA	0.56 ± 0.10	0.24	17.8	17.1 ± 1.4
	IIIB	0.04 ± 0.00	0.76	32.0	35.2 ± 0.5
	Experiment	E_{exp}		$R(E_{\text{exp}})$ (Å)	
		**0.80 ± 0.03**		**14.6**	
N-(1-Naphthyl)ethylenediamine					
	TrESP-MMPol@MD	E_{theo}	$\lvert E_{\text{exp}} - E_{\text{theo}}\rvert$	$R(E_{\text{theo}})$ (Å)	R_{MD} (Å)
	**Cleft**	**0.64 ± 0.07**	**0.09**	**18.3**	**17.4 ± 0.3**
	IB	0.26 ± 0.04	0.29	24.7	22.7 ± 0.4
	IIA-IIB	0.91 ± 0.05	0.36	12.4	12.0 ± 0.3
	IIA	1.00 ± 0.00	0.45	4.9	5.7 ± 0.1
	**IIIA**	**0.43 ± 0.08**	**0.12**	**21.7**	**20.3 ± 0.9**
	IIIB	0.06 ± 0.03	0.49	35.2	35.3 ± 0.6
	Experiment	E_{exp}		$R(E_{\text{exp}})$ (Å)	
		**0.55 ± 0.02**		**19.6**	
9-Acridinecarboxylic acid					
	TrESP-MMPol@MD	E_{theo}	$\lvert E_{\text{exp}} - E_{\text{theo}}\rvert$	$R(E_{\text{theo}})$ (Å)	R_{MD} (Å)
	Cleft	0.72 ± 0.05	0.09	17.0	18.1 ± 0.5
	IB	0.13 ± 0.03	0.50	29.5	26.3 ± 0.7
	IIA-IIB	0.97 ± 0.01	0.34	9.9	10.6 ± 0.3
	IIA	1.00 ± 0.00	0.37	4.9	5.0 ± 0.5
	**IIIA**	**0.73 ± 0.04**	**0.10**	**17.0**	**17.7 ± 0.6**
	IIIB	0.03 ± 0.00	0.60	37.0	35.5 ± 0.5
	Experiment	E_{exp}		$R(E_{\text{exp}})$ (Å)	
		**0.63 ± 0.02**		**18.5**	

9-Acridinecarboxylic acid (protonated)					
	TrESP-MMPol@MD	E_{theo}	$\|E_{exp} - E_{theo}\|$	$R(E_{theo})$ (Å)	R_{MD} (Å)
	**Cleft**	**0.75 ± 0.09**	**0.12**	**16.5**	**17.8 ± 0.7**
	IB	0.34 ± 0.05	0.29	24.0	31.0 ± 1.0
	IIA-IIB	0.98 ± 0.01	0.35	8.9	10.5 ± 0.4
	IIA	1.00 ± 0.00	0.37	6.3	4.1 ± 0.1
	IIIA	0.42 ± 0.07	0.21	22.6	19.8 ± 0.9
	IIIB	0.09 ± 0.03	0.54	33.4	39.6 ± 1.7
	Experiment	E_{exp}		$R(E_{exp})$ (Å)	
		**0.63 ± 0.02**		**19.1**	
Carprofen					
	TrESP-MMPol@MD	E_{theo}	$\|E_{exp} - E_{theo}\|$	$R(E_{theo})$ (Å)	R_{MD} (Å)
	Cleft	0.86 ± 0.02	0.05	16.9	17.3 ± 0.2
	IB	0.51 ± 0.05	0.30	23.3	23.7 ± 0.5
	**IIA-IIB**	**0.94 ± 0.01**	**0.13**	**14.0**	**10.7 ± 0.2**
	IIA	1.00 ± 0.00	0.19	6.7	5.2 ± 0.2
	**IIIA**	**0.85 ± 0.02**	**0.04**	**17.1**	**18.1 ± 0.2**
	IIIB	0.06 ± 0.01	0.75	39.2	34.3 ± 0.7
	Experiment	E_{exp}		$R(E_{exp})$ (Å)	
		**0.81 ± 0.03**		**18.1**	
Naproxen					
	TrESP-MMPol@MD	E_{theo}	$\|E_{exp} - E_{theo}\|$	$R(E_{theo})$ (Å)	R_{MD} (Å)
	Cleft	0.63 ± 0.05	0.06	17.1	16.7 ± 0.4
	IB	0.26 ± 0.05	0.31	23.4	23.7 ± 0.4
	**IIA-IIB**	**0.88 ± 0.03**	**0.31**	**12.8**	**10.5 ± 0.2**
	IIA	1.00 ± 0.00	0.43	6.3	5.0 ± 0.1
	**IIIA**	**0.41 ± 0.09**	**0.16**	**21.1**	**22.7 ± 1.4**
	IIIB	0.04 ± 0.01	0.53	33.5	33.6 ± 0.2
	Experiment	E_{exp}		$R(E_{exp})$ (Å)	
		**0.57 ± 0.01**		**17.8**	
(Quinolin-8-yloxy)-acetic acid					
	TrESP-MMPol@MD	E_{theo}	$\|E_{exp} - E_{theo}\|$	$R(E_{theo})$ (Å)	R_{MD} (Å)
	**Cleft**	**0.65 ± 0.06**	**0.05**	**16.4**	**16.5 ± 0.4**
	IB	0.22 ± 0.05	0.38	23.2	22.1 ± 0.4
	IIA-IIB	0.92 ± 0.01	0.32	11.8	11.4 ± 0.2
	IIA	1.00 ± 0.00	0.40	5.3	5.0 ± 0.7
	IIIA	0.33 ± 0.08	0.27	21.6	19.3 ± 0.5
	IIIB	0.03 ± 0.00	0.57	34.0	34.7 ± 0.6
	Experiment	E_{exp}		$R(E_{exp})$ (Å)	
		**0.60 ± 0.01**		**17.1**	
Indomethacin					
	TrESP-MMPol@MD	E_{theo}	$\|E_{exp} - E_{theo}\|$	$R(E_{theo})$ (Å)	R_{MD} (Å)
	Cleft	0.79 ± 0.04	0.06	15.6	17.7 ± 0.6
	**IB**	**0.25 ± 0.04**	**0.48**	**25.2**	**25.4 ± 0.9**
	IIA-IIB	0.91 ± 0.03	0.18	12.0	11.2 ± 0.6
	**IIA**	**1.00 ± 0.00**	**0.27**	**4.8**	**3.9 ± 0.1**
	IIIA	0.59 ± 0.08	0.14	19.3	21.4 ± 0.9
	IIIB	0.07 ± 0.02	0.66	32.5	35.3 ± 0.7
	Experiment	E_{exp}		$R(E_{exp})$ (Å)	
		**0.73 ± 0.03**		**17.2**	

Table S4. Summary of statistical errors between average ligand-Trp distances sampled along MD trajectories (R_{MD} in Table S3) and distances estimated from TrESP-MMPol@MD theoretical efficiencies assuming R^{-6} Förster expression ($R(E_{\mathrm{theo}})$ in Table S3). Deviations of $R(E_{\mathrm{theo}})$ from R_{MD} arise from the limits of Förster point dipole approximation and the assumption of an isotropic distribution of donor-acceptor orientations. Minimum error, maximum error, mean signed error (MSE) and mean unsigned error (MUE) in Å.

$R(E_{\mathrm{theo}})$ - R_{MD} per ligand (all binding sites)				
	Min	Max	MSE	MUE
1-Naphthol	-4.9	0.2	-0.9	1.0
2-Naphthol	-3.2	0.8	-0.5	1.2
N-(1-Naphthyl)ethylenediamine	-0.8	2.0	0.6	0.9
9-Acridinecarboxylic acid	-1.1	3.2	0.4	1.2
9-Acridinecarboxylic acid (protonated)	-7.0	2.8	-1.9	3.5
Carprofen	-1.0	4.9	1.3	1.9
Naproxen	-1.6	2.3	0.3	1.0
(Quinolin-8-yloxy)-acetic acid	-0.7	2.3	0.5	0.8
Indomethacin	-2.8	0.9	-0.9	1.5
$R(E_{\mathrm{theo}})$ - R_{MD} per binding site (all ligands)				
	Min	Max	MSE	MUE
Cleft	-2.1	0.9	-0.3	0.8
IB	-7.0	3.2	-0.1	1.7
IIA-IIB	-1.6	3.3	0.4	1.2
IIA	-0.8	2.2	0.6	0.8
IIIA	-2.1	2.8	0.2	1.4
IIIB	-6.2	4.9	-1.3	2.7
**All sites**	**-7.0**	**4.9**	**-0.1**	**1.4**

Table S5. Summary of statistical errors in FRET efficiencies for ligands in the binding sites of HSA obtained from PDA@MD ($\kappa^2 = 2/3$, isotropic orientation factor) and PDA@MD (κ^2 values calculated from MD) models with respect to TrESP-MMPol@MD values. Minimum error, maximum error, mean signed error (MSE) and mean unsigned error (MUE).

E(PDA@MD, $\kappa^2 = 2/3$) - E(TrESP-MMPol@MD) per ligand (all binding sites)	Min	Max	MSE	MUE
1-Naphthol	-0.04	0.02	0.00	0.01
2-Naphthol	-0.03	0.06	0.02	0.04
N-(1-Naphthyl)ethylenediamine	-0.02	0.08	0.04	0.05
9-Acridinecarboxylic acid	-0.06	0.06	0.00	0.03
9-Acridinecarboxylic acid (protonated)	-0.24	0.16	-0.03	0.09
Carprofen	-0.05	0.05	0.00	0.03
Naproxen	-0.11	0.09	-0.01	0.05
(Quinolin-8-yloxy)-acetic acid	-0.01	0.09	0.02	0.03
Indomethacin	-0.15	0.05	-0.04	0.06
**E(PDA@MD, $\kappa^2 = 2/3$) - E(TrESP-MMPol@MD) per binding site (all ligands)**				
	Min	Max	MSE	MUE
Cleft	-0.09	0.07	-0.01	0.04
IB	-0.24	0.08	-0.02	0.07
IIA-IIB	-0.03	0.09	0.03	0.04
IIA	0.00	0.00	0.00	0.00
IIIA	-0.15	0.16	0.00	0.08
IIIB	-0.07	0.03	-0.01	0.02
All sites	-0.24	0.16	0.00	0.04
**E(PDA@MD) - E(TrESP-MMPol@MD) per ligand (all binding sites)**				
	Min	Max	MSE	MUE
1-Naphthol	-0.13	0.00	-0.06	0.06
2-Naphthol	-0.17	0.00	-0.07	0.07
N-(1-Naphthyl)ethylenediamine	-0.18	0.00	-0.08	0.08
9-Acridinecarboxylic acid	-0.18	0.00	-0.08	0.08
9-Acridinecarboxylic acid (protonated)	-0.23	0.00	-0.11	0.11
Carprofen	-0.23	0.04	-0.06	0.09
Naproxen	-0.22	0.02	-0.08	0.08
(Quinolin-8-yloxy)-acetic acid	-0.10	-0.01	-0.06	0.06
Indomethacin	-0.17	0.00	-0.08	0.08
**E(PDA@MD) - E(TrESP-MMPol@MD) per binding site (all ligands)**				
	Min	Max	MSE	MUE
Cleft	-0.23	-0.05	-0.16	0.16
IB	-0.23	0.04	-0.07	0.08
IIA-IIB	-0.17	0.04	-0.05	0.06
IIA	-0.01	0.00	0.00	0.00
IIIA	-0.23	-0.09	-0.15	0.15
IIIB	-0.06	0.00	-0.02	0.02
All sites	-0.23	0.04	-0.07	0.08

Table S6. Comparison of FRET efficiencies and binding parameters for HSA-ligand complexes measured from fluorescence spectra with excitation wavelengths 280 nm and 295 nm including or not inner-filter effects (IFE) corrections based on the absorbance of the ligands at the excitation and emission wavelengths ($I_{DA,cor} = I_{DA}10^{(Abs_{ex}+Abs_{em})/2}$).[3]

	Experiment	E_{exp}	K_d / μM	n
1-Naphthol	λ_{ex} = 280 nm, no correction	0.83± 0.01	104.1	0.92
	λ_{ex} = 295 nm, no correction	0.79± 0.03	104.6	0.91
	λ_{ex} = 280 nm, IFE-corrected	0.54± 0.03	127.6	0.78
	λ_{ex} = 295 nm, IFE-corrected	**0.46± 0.05**	**137.0**	**0.73**
2-Naphthol	λ_{ex} = 280 nm, no correction	0.87± 0.03	77.0	0.95
	λ_{ex} = 295 nm, no correction	0.82± 0.03	6.9	0.75
	λ_{ex} = 280 nm, IFE-corrected	0.71± 0.02	64.5	0.85
	λ_{ex} = 295 nm, IFE-corrected	**0.80 ± 0.03**	**6.6**	**0.71**
N-(1-Naphthyl)ethylenediamine	λ_{ex} = 280 nm, no correction	0.57± 0.03	5.6	0.45
	λ_{ex} = 295 nm, no correction	0.67± 0.03	5.0	0.53
	λ_{ex} = 280 nm, IFE-corrected	0.45± 0.01	3.9	0.26
	λ_{ex} = 295 nm, IFE-corrected	**0.55 ± 0.02**	**3.5**	**0.33**
9-Acridinecarboxylic acid	λ_{ex} = 280 nm, no correction	0.75± 0.02	20.9	0.80
	λ_{ex} = 295 nm, no correction	0.72± 0.02	18.3	0.76
	λ_{ex} = 280 nm, IFE-corrected	0.64± 0.02	17.5	0.70
	λ_{ex} = 295 nm, IFE-corrected	**0.63 ± 0.02**	**15.5**	**0.67**
Carprofen	λ_{ex} = 280 nm, no correction	0.89± 0.02	3.0	0.74
	λ_{ex} = 295 nm, no correction	0.71± 0.03	2.8	0.46
	λ_{ex} = 280 nm, IFE-corrected	**0.81 ± 0.03**	**1.8**	**0.48**
	λ_{ex} = 295 nm, IFE-corrected	0.42± 0.04	0.68	-0.22
Naproxen	λ_{ex} = 280 nm, no correction	0.78± 0.02	18.5	0.81
	λ_{ex} = 295 nm, no correction	0.76± 0.01	30.3	0.85
	λ_{ex} = 280 nm, IFE-corrected	0.37± 0.00	8.2	0.33
	λ_{ex} = 295 nm, IFE-corrected	**0.57 ± 0.01**	**24.4**	**0.68**
(Quinolin-8-yloxy)-acetic acid	λ_{ex} = 280 nm, no correction	0.80± 0.01	75.9	0.90
	λ_{ex} = 295 nm, no correction	0.76± 0.01	54.8	0.86
	λ_{ex} = 280 nm, IFE-corrected	0.68± 0.01	74.5	0.83
	λ_{ex} = 295 nm, IFE-corrected	**0.60 ± 0.01**	**48.1**	**0.76**
Indomethacin	λ_{ex} = 280 nm, no correction	1.00± 0.02	7.8	1.08
	λ_{ex} = 295 nm, no correction	0.86± 0.03	4.1	0.72
	λ_{ex} = 280 nm, IFE-corrected	0.84± 0.03	3.7	0.69
	λ_{ex} = 295 nm, IFE-corrected	**0.73 ± 0.03**	**2.4**	**0.45**

REFERENCES

[1] J. Ghuman, P. A. Zunszain, I. Petitpas, A. A. Bhattacharya, M. Otagiri, S. Curry, "Structural Basis of the Drug-binding Specificity of Human Serum Albumin" *J. Mol. Biol.* **2005**, *353*, 38–52.

[2] I. Vayá, P. Bonancía, M. C. Jiménez, D. Markovitsi, T. Gustavsson, M. A. Miranda, "Excited state interactions between flurbiprofen and tryptophan in drug–protein complexes and in model dyads. Fluorescence studies from the femtosecond to the nanosecond time domains" *Phys. Chem. Chem. Phys.* **2013**, *15*, 4727.
